# Melatonin Induction of APP Intracellular Domain 50 SUMOylation Alleviates AD through Enhanced Transcriptional Activation and Aβ Degradation

**DOI:** 10.1016/j.ymthe.2020.09.003

**Published:** 2020-09-05

**Authors:** Yen-Chen Liu, Wei-Lun Hsu, Yun-Li Ma, Eminy H.Y. Lee

**Affiliations:** 1 Graduate Institute of Life Sciences, National Defense Medical Center, Taipei 114, Taiwan; 2 Institute of Biomedical Sciences, Academia Sinica, Taipei 115, Taiwan

**Keywords:** APP intracellular domain, PIAS1, SUMOylation, Fe65, CREB, p65, neprilysin, transthyretin, amyloid-beta degradation, melatonin

## Abstract

The amyloid precursor protein (APP) intracellular domain (AICD) is implicated in the pathogenesis of Alzheimer’s disease (AD), but post-translational modification of AICD has rarely been studied and its role in AD is unknown. In this study, we examined the role and molecular mechanism of AICD SUMOylation in the pathogenesis of AD. We found that AICD is SUMO-modified by the SUMO E3 ligase protein inhibitor of activated STAT1 (PIAS1) in the hippocampus at Lys-43 predominantly, and that knockdown of PIAS1 decreases endogenous AICD SUMOylation. AICD SUMOylation increases AICD association with its binding protein Fe65 and increases AICD nuclear translocation. Furthermore, AICD SUMOylation increases AICD association with cyclic AMP-responsive element binding protein (CREB) and p65 and their DNA binding for transcriptional activation of neprilysin (NEP) and transthyretin (TTR), two major Aβ-degrading enzymes, respectively. Consequently, AICD SUMOylation decreases the Aβ level, Aβ oligomerization, and amyloid plaque deposits. It also rescues spatial memory deficits in APP/PS1 mice. Conversely, blockade of AICD SUMOylation at Lys-43 produces the opposite effects. Melatonin is identified as an endogenous stimulus that induces AICD SUMOylation. It also decreases the Aβ level and rescues reduction of PIAS1, NEP, and TTR expression in APP/PS1 mice. In this study, we demonstrate that AICD SUMOylation functions as a novel endogenous defense mechanism to combat AD.

## Graphical Abstract

**Figure:**
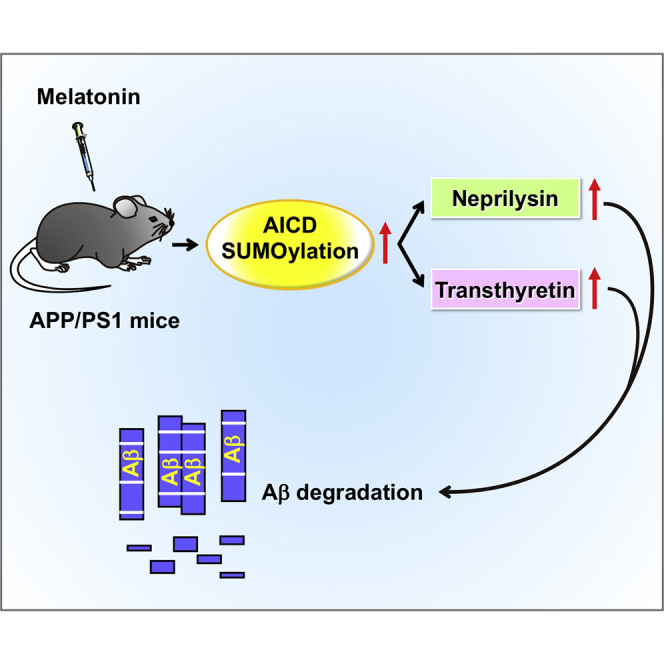

## Teaser

Lee and colleagues have found that AICD SUMOylation enhances AICD nuclear translocation and transcriptional activation of neprilysin and transthyretin for amyloid-beta degradation. AICD SUMOylation also decreases amyloid plaques and rescues spatial memory deficits in APP/PS1 mice. Melatonin is an endogenous stimulus that induces AICD SUMOylation to combat Alzheimer’s disease.

## Introduction

The amyloid precursor protein (APP) intracellular domain (AICD) is known to regulate apoptosis, cytoskeletal dynamics, cell cycle re-entry, DNA repair, nuclear signaling, and transcriptional regulation, all of which are involved in Alzheimer’s disease (AD).^1^ AICD is generated through both the amyloidogenic pathway and nonamyloidogenic pathway. In the amyloidogenic pathway, which mainly occurs in the endosome, AICD is generated via sequential, proteolytic cleavages of APP by β-secretase (BACE1) and γ-secretase.^2^ AICD then interacts with other proteins, including Fe65 and Tip60, and translocates to the nucleus for nuclear signaling and regulation of gene transcription.^3^^,^^4^ In the nonamyloidogenic pathway, which mainly acts at the cell membrane, AICD is generated via sequential, proteolytic cleavages of APP by α-secretase and γ-secretase.^1^^,^^5^ Regardless of the pathway by which AICD is generated, it is unstable and easily degraded through cleavage by γ-secretase.^6^ Similar to the Notch intracellular domain, nuclear AICD is suggested to function as a transcriptional regulator.^7^^,^^8^ Previous studies have shown that AICD directly binds to the promoter of neprilysin (NEP), an Aβ-degrading enzyme, and regulates NEP expression.^9^^,^^10^ Transthyretin (TTR) was originally recognized as a transport protein for thyroxine and retinol. Additional studies revealed that Aβ is a protease substrate of TTR.^11^ TTR was found to bind to Aβ and protect against Aβ toxicity by proteolytic cleavage of Aβ and inhibition of Aβ aggregation.^12^^,^^13^ When γ-secretase activity was inhibited in cells overexpressing APP695 (in which AICD production is presumably reduced), the *TTR* mRNA level was found to be decreased,^14^ suggesting that AICD regulates *TTR* mRNA expression.

APP is subject to several posttranslational modifications. APP was found to be phosphorylated at seven residues. Among these residues, the Thr-668 phosphorylation level was found to be significantly increased in the hippocampi of AD patients.^15^ APP was also found to be ubiquitinated at several residues (Lys-649–Lys-651 and Lys-688), with APP ubiquitination regulating Aβ generation and APP maturation and degradation.^16^^,^^17^ In addition to phosphorylation and ubiquitination, neddylation also occurs at multiple lysine residues of APP, with Nedd8 conjugation to the APP C-terminal fragment impairing the interaction of AICD with Fe65 and inhibiting AICD-mediated transcriptional activation.^18^ Furthermore, APP was found to be SUMO-modified by the SUMO E2 ligase Ubc9 at Lys-587 and Lys-595, and APP SUMOylation decreased the level of Aβ aggregates in cells transfected with AD-associated mutant APP.^19^

Protein SUMOylation plays important roles in the regulation of various cellular functions.^20^^,^^21^ In the context of AD, we recently showed that enhanced SUMOylation of histone deacetylase 1 (HDAC1) and Elk-1 promotes neuronal survival and protects against Aβ toxicity in APP/PS1 mice.^22^^,^^23^ Although a previous report indicated SUMOylation of APP, it is not known whether the AICD protein can be SUMO-modified and what the functional significance of such AICD SUMOylation would be. In this study, we sought to examine the role and molecular mechanism of AICD SUMOylation in the pathogenesis of AD by adopting APP/PS1 mice as a mouse model of AD. Our results reveal that AICD is SUMO-modified by the protein inhibitor of activated STAT1 (PIAS1), a SUMO E3 ligase, in the hippocampus endogenously and that AICD SUMOylation decreases the level of Aβ and amyloid plaque by facilitating the clearance of Aβ. AICD SUMOylation also rescues spatial learning and memory deficits in APP/PS1 mice. In addition, melatonin is identified as an endogenous stimulus that induces AICD SUMOylation. In this study, we have identified a novel posttranslational regulation of AICD and a novel defense mechanism protecting against AD through AICD SUMOylation.

## Results

### AICD50 Is SUMO-Modified by PIAS1 at Lys-43 in Cells

**Figure 1:**
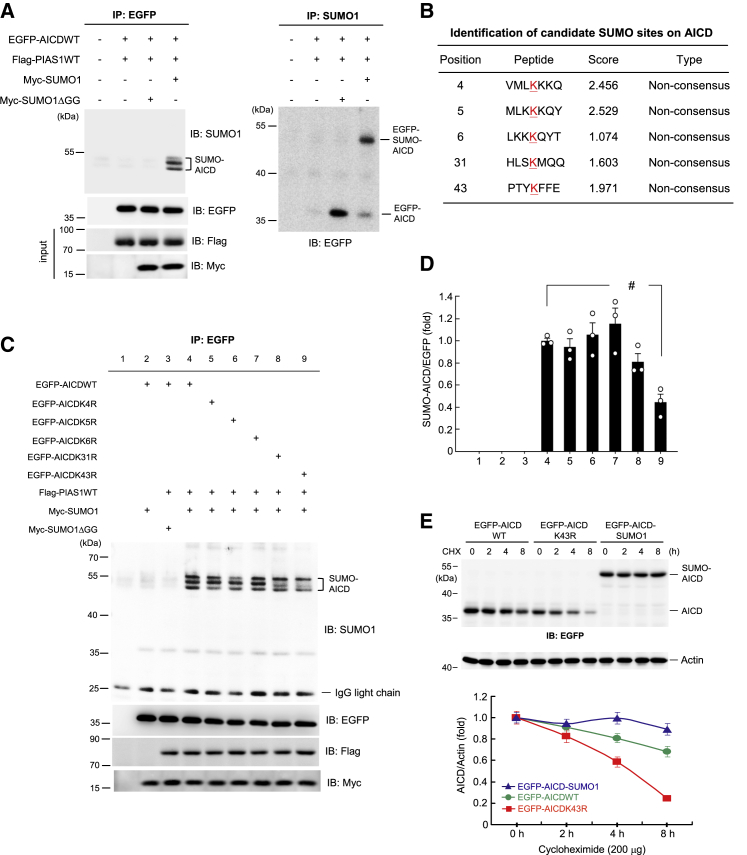
Identification of Candidate SUMO Sites on AICD (A) EGFP-AICD plasmid, FLAG-PIAS1 plasmid, and Myc-SUMO1 (or SUMO1ΔGG) plasmid were co-transfected into HEK293T cells. Cell lysate was immunoprecipitated with anti-EGFP antibody and immunoblotted with anti-SUMO1 antibody. The SUMO-AICD bands were observed. Cell lysate was also immunoblotted with anti-EGFP, anti-FLAG, and anti-Myc antibodies to confirm the transfection and expression of various plasmids (left panel). Cell lysates were also immunoprecipitated with anti-SUMO1 antibody and immunoblotted with anti-EGFP antibody (right panel). (B) SUMO2.0 software prediction of candidate SUMO acceptors on AICD. The underlined letter “K” indicates the candidate SUMO sites. (C) EGFP-tagged AICDWT plasmid or individual lysine mutant plasmids, FLAG-PIAS1 plasmid, and Myc-SUMO1 (or SUMO1ΔGG) plasmid were co-transfected into HEK293T cells. Cell lysate was immunoprecipitated with anti-EGFP antibody and immunoblotted with anti-SUMO1 antibody. The SUMO-AICD bands under each condition are shown. Cell lysate was also immunoblotted with anti-EGFP, anti-FLAG, and anti-Myc antibodies to confirm the transfection and expression of various plasmids. (D) Quantified results of (C) (F_8,18_ = 45.81, q = 7.61, p < 0.001 comparing lane 4 and lane 9). (E) Different EGFP-tagged AICD plasmids were transfected into HEK293T cells (200 ng per well). Cycloheximide (200 μg/mL) was added to the cell 24 h after plasmid transfection for different time periods (0, 2, 4, and 8 h). Cell lysates were prepared for western blotting of AICD expression using anti-EGFP antibody. CHX, cycloheximide. The quantified results of EGFP-AICD expression for each group are also shown. Experiments are in three repeats for (A) and (C), and four repeats for (E). Data are expressed as individual values and mean ± SEM. ^#^p < 0.001.

As mentioned in the Materials and Methods, in the present study we examined AICD50 protein (abbreviated as AICD hereinafter). We first examined whether AICD could be SUMO-modified by PIAS1 in cells. Different combinations of the EGFP-, FLAG- and Myc-tagged plasmids were transfected into HEK293T cells, and the cell lysate was immunoprecipitated with anti-EGFP antibody and immunoblotted with anti-SUMO1 antibody. The results revealed that AICD was SUMO-modified in cells probably at more than one residue, but that this effect was completely blocked when Myc-SUMO1ΔGG plasmid, instead of Myc-SUMO1 plasmid, was transfected to block SUMO conjugation (Figure 1A, left panel). The same cell lysates were also immunoprecipitated with anti-SUMO1 antibody and immunoblotted with anti-EGFP antibody. The results showed that the AICD-SUMO1 band was observed only when the Myc-SUMO1 plasmid was transfected (Figure 1A, right panel). Because SUMO substrate proteins often contain the ψ-K-X-E motif, where ψ stands for a hydrophobic amino acid, we next performed a bioinformatics analysis using SUMO2.0 software.^24^ The results indicated that there are five lysine residues on the AICD protein, but there is no consensus SUMO-substrate motif on it (Figure 1B). Because even proteins that do not contain the ψ-K-X-E motif can still be SUMO-modified and produce biological functions (e.g., cyclic AMP-responsive element binding protein [CREB]),^25^ we generated individual AICD lysine residue mutants and transfected EGFP-AICD wild-type (WT) plasmid or individual EGFP-AICD mutant plasmids together with FLAG-PIAS1WT plasmid and Myc-SUMO1 plasmid (or Myc-SUMO1ΔGG plasmid) into HEK293T cells. Co-immunoprecipitation (coIP) experiments were carried out as described above. The results confirmed that AICD could be SUMO-modified by PIAS1. Multiple SUMO-AICD bands were observed but AICD was predominantly SUMOylated at Lys-43 (Figure 1C). The quantified AICD SUMOylation level at each mutation site is shown in Figure 1D. Similar results were obtained when the cell lysate was directly immunoblotted with anti-AICD antibody (Figure S1). Because Lys-43 is the major SUMO residue on AICD, we focused on this residue in the present study. To understand the cellular effect of AICD SUMOylation, we transfected EGFP-AICDWT, EGFP-AICDK43R, and EGFP-AICD-SUMO1 fusion plasmids into HEK293T cells with cycloheximide added to the cells for different time periods. Western blotting was carried out using anti-EGFP antibody. The results revealed that AICD SUMOylation stabilizes AICD whereas the SUMO mutant AICD degrades more rapidly (Figure 1E).

### AICD Is SUMO-Modified by PIAS1 Endogenously and Co-localizes with PIAS1 in the Hippocampus

**Figure 2:**
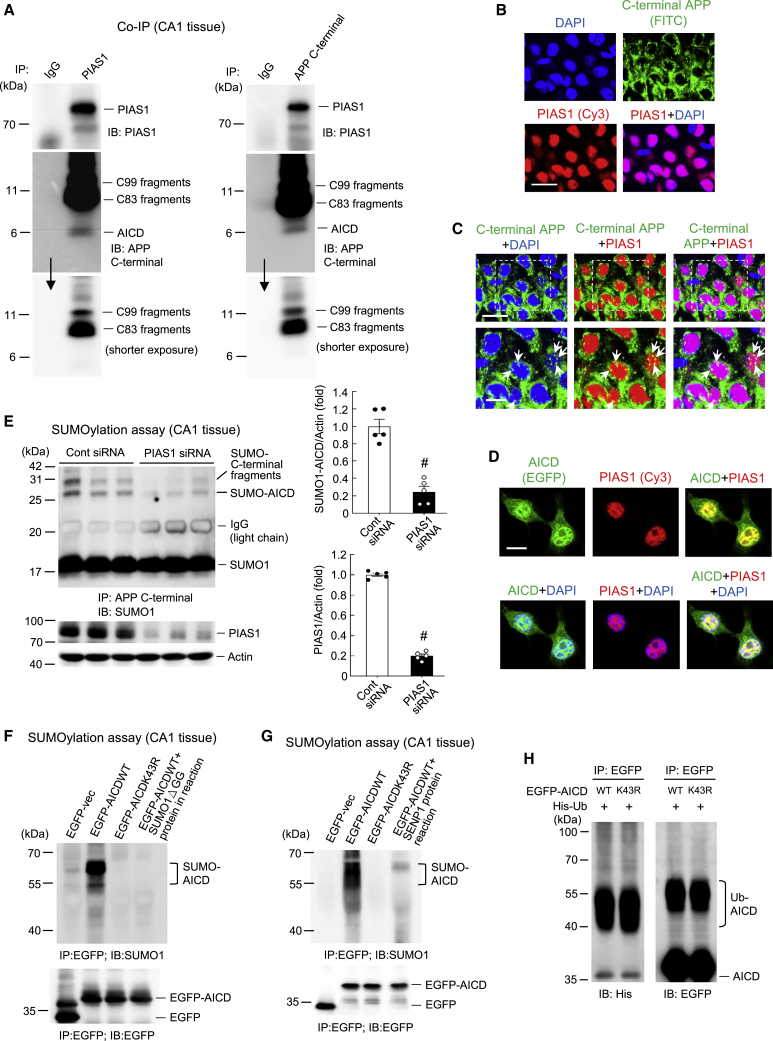
AICD Is SUMO-Modified by PIAS1 Endogenously and Is Co-localized with PIAS1 in Hippocampal Neurons (A) Rat CA1 tissue lysate was immunoprecipitated with anti-PIAS1 antibody and immunoblotted with anti-APP C-terminal antibody and anti-PIAS1 antibody (left) and vice versa (right). Experiments are in two repeats. (B) Immunohistochemistry showing the distribution of PIAS1 and C-terminal APP in hippocampal neurons (n = 3). Scale bar represents 20 μm. (C) Immunohistochemistry at a higher magnification showing co-localization of PIAS1 and C-terminal APP in the nucleus of hippocampal neurons (indicated by arrows). Scale bars represent 20 μm for the upper panel and 10 μm for the lower panel. Magnification of the dotted square area is shown in the lower panel. (D) EGFP-AICD plasmid was transfected into HEK293T cells. Cy3-conjugated secondary antibody against the PIAS1 antibody was used for visualization of PIAS1. Immunofluorescence staining showing the co-localization of AICD (green) and PIAS1 (red) in the nucleus only (n = 3). Scale bar represents 10 μm. (E) PIAS1 siRNA (10 pmol) or control siRNA was transfected into the rat CA1 area, and endogenous AICD SUMOylation as well as C-terminal fragment SUMOylation were determined 48 h later by a SUMOylation assay. PIAS1 expression was determined by western blot (n = 5). The quantified results of AICD SUMOylation and PIAS1 expression are shown in the right panel (t_1,8_ = 7.35 for AICD SUMOylation and t_1,8_ = 32.91 for PIAS1, both p < 0.001). Data are expressed as individual values and mean ± SEM. (F) EGFP-AICDWT (with or without the SUMO1ΔGG mutant protein added to the reaction) or EGFP-AICDK43R plasmid was transfected into the rat CA1 area, and a SUMOylation assay was carried out 48 h later. The SUMO-AICD bands in the EGFP-AICDWT group are shown. coIP with anti-EGFP antibody was conducted to confirm the expression of the plasmids. (G) The same plasmids were transfected into another batch of rats except that recombinant SENP1, instead of mutant SUMO1, protein was added to the reaction for the last group. A SUMOylation assay was carried out 48 h later. The SUMO-AICD bands in the EGFP-AICDWT group are shown. coIP with anti-EGFP antibody was conducted to confirm the expression of the plasmids. (H) EGFP-AICDWT or EGFP-AICDK43R plasmid was co-transfected with histidine (His)-ubiquitin plasmid to HEK293T cells. Cell lysate was immunoprecipitated with anti-EGFP antibody and immunoblotted with anti-His and anti-EGFP antibody. Ubiquitinated AICD is shown. Ub, ubiquitin. Experiments are in two repeats for (G) and three repeats for (F) and (H). ^#^p < 0.001.

After showing that AICD is SUMO-modified by PIAS1 in cells, we next examined whether AICD could be SUMO-modified by PIAS1 endogenously in the brain. We first examined the relationship between AICD and PIAS1 in the hippocampus by carrying out coIP experiments. Because commercially available AICD antibodies also recognize full-length APP, we used the APP C-terminal antibody for this experiment. Rat hippocampal CA1 tissue was immunoprecipitated with anti-PIAS1 antibody and immunoblotted with anti-APP C-terminal antibody (as well as anti-PIAS1 antibody) or vice versa. The results revealed that PIAS1 is associated with the C99 fragment, C83 fragment, and AICD in the hippocampus endogenously (Figure 2A). Next, we examined whether PIAS1 and AICD are present in the same neurons in the rat hippocampus. Brain sections containing the CA1 region were subjected to immunohistochemical staining. Antibody against PIAS1, Cy3-conjugated secondary antibody, antibody against the APP C-terminal, and FITC-conjugated secondary antibody were used. DAPI was added to the reaction for nuclear staining. The results revealed that PIAS1 (red) was present only in the nucleus. APP C-terminal staining was mainly observed in the cytosol and the surrounding area of the nucleus (green) (Figure 2B). Further visualization at a higher magnification indicated that APP C-terminal staining was also present in the nucleus and that the APP C-terminal co-localized with PIAS1 in the same neurons (yellow; indicated by arrows) (Figure 2C, lower panel). Although the APP C-terminal antibody would be expected to also stain the C83 fragment and C99 fragment in addition to AICD, only AICD nuclear translocation was observed.^3^ Thus, the staining we observed in the nucleus is presumably the AICD protein. To further examine the co-localization of AICD with PIAS1, HEK293T cells were transfected with the EGFP-AICD plasmid. PIAS1 immunofluorescence was visualized using Cy3-conjugated secondary antibody against the PIAS1 antibody. The results revealed that overexpressed AICD co-localized with endogenous PIAS1 only in the nucleus (Figure 2D).

The above results showed that PIAS1 is associated with AICD and co-localizes with AICD in the same hippocampal neurons. Next, we examined whether AICD is SUMO-modified by PIAS1 in the hippocampus endogenously. Control small interfering RNA (siRNA) or PIAS1 siRNA (10 pmol) was transfected into the rat CA1 area and AICD SUMOylation was determined 48 h later. The results revealed that PIAS1 siRNA transfection significantly decreased the levels of both endogenous AICD SUMOylation (lower band) and C-terminal fragment SUMOylation (upper band) (Figure 2E). PIAS1 siRNA transfection also markedly decreased the level of PIAS1 expression (Figure 2E, lower panel). The quantified results of AICD SUMOylation and PIAS1 expression are shown in Figure 2E (right panel).

To further explore AICD SUMOylation by PIAS1 in the hippocampus, we transfected EGFP-AICDWT plasmid and EGFP-AICDK43R plasmid into the rat CA1 area and carried out an *in vitro* SUMOylation assay. This approach was taken because mutation of AICD at Lys-43 caused the greatest decrease in AICD SUMOylation in cells (Figures 1C and 1D). Cell lysate was immunoprecipitated with anti-EGFP antibody and immunoblotted with anti-SUMO1 antibody. The results indicated that AICD was apparently SUMOylated by PIAS1, but that AICD SUMOylation was completely blocked when EGFP-AICDK43R was transfected. To confirm the specificity of the AICD SUMOylation reaction, in another group we added the SUMO1ΔGG mutant protein to the reaction because this mutant prevents the SUMO molecule from forming a covalent bond with the lysine residue on AICD. The results revealed that AICD SUMOylation was abolished when EGFP-AICDWT plasmid was transfected and the SUMO1ΔGG mutant protein was added to the SUMOylation reaction mixture at the same time (Figure 2F). To further examine AICD SUMOylation by PIAS1, another group of rats received the same plasmid transfections as described above except that, instead of the SUMO1ΔGG protein, the recombinant SENP1 protein was added to the reaction. This group was included because the SENP1 enzyme removes the SUMO molecule from the lysine residue of a SUMOylated protein. The results revealed that addition of SENP1 similarly abolished AICD SUMOylation (Figure 2G).

Because ubiquitination of APP was found to take place at Lys-649–Lys-651 and Lys-688,^16^^,^^17^ we next examined whether Lys-43 of AICD can be ubiquitinated and, if so, whether this ubiquitination affects AICD SUMOylation. EGFP-AICDWT or EGFP-AICDK43R plasmid was co-transfected with His-ubiquitin plasmid into HEK293T cells and cell lysate was immunoprecipitated with anti-EGFP antibody and immunoblotted with anti-His antibody and anti-EGFP antibody. The results revealed that AICD ubiquitination was not affected by EGFP-AICDK43R transfection regardless of whether it was immunoblotted with anti-His antibody or anti-EGFP antibody (Figure 2H).

### AICD SUMOylation Increases Its Association with Fe65 and Decreases Its Association with HDAC1

**Figure 3:**
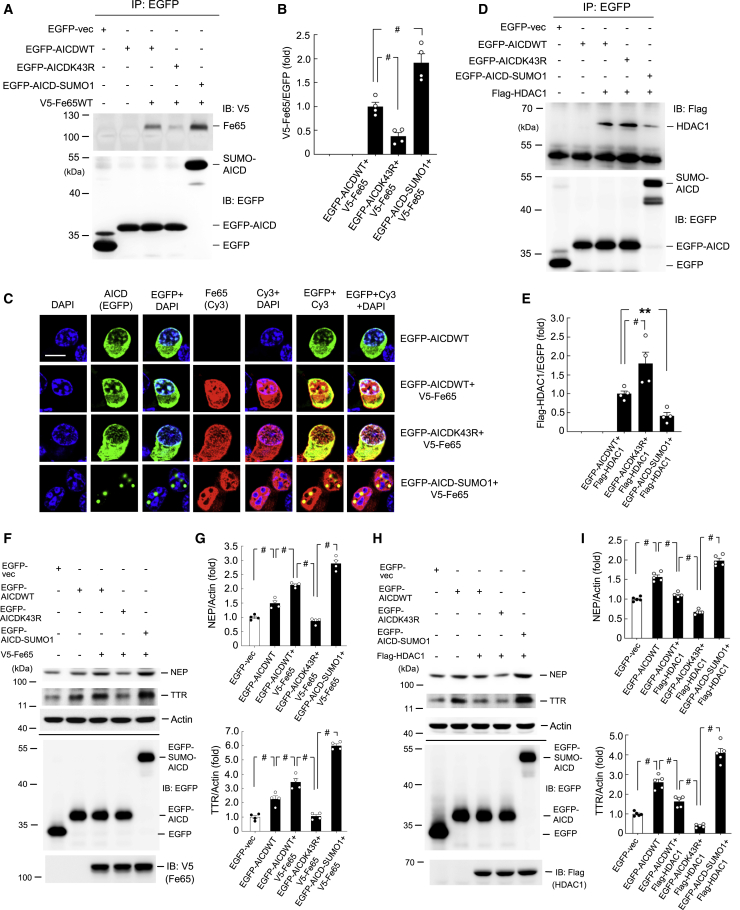
AICD SUMOylation Increases Its Association with Fe65 and Decreases Its Association with HDAC1 (A) Different EGFP-tagged AICD plasmids and V5-Fe65 plasmid were co-transfected into HEK293T cells. Cell lysate was immunoprecipitated with anti-EGFP antibody and immunoblotted with anti-V5 antibody. CoIP with anti-EGFP antibody was conducted to confirm the expression of the AICD plasmids. (B) Quantified results of (A) (F_4,15_ = 66.54, p < 0.001; q = 6.26, p < 0.001 comparing the AICDK43R+Fe65 group with the AICDWT+Fe65 group; and q = 9.2, p < 0.001 comparing the AICD-SUMO1+Fe65 group with the AICDWT+Fe65 group). (C) Different EGFP-tagged AICD plasmids and V5-Fe65 plasmid were co-transfected into Neuro2A cells and the distributions of AICD and Fe65 and their co-localization with DAPI were examined by immunofluorescence staining using FITC- and Cy3-conjugated secondary antibodies, respectively. Scale bar represents 10 μm. Results are from three different batches of cells. (D) Different EGFP-tagged AICD plasmids and FLAG-HDAC1 plasmid were co-transfected into HEK293T cells. Cell lysate was immunoprecipitated with anti-EGFP antibody and immunoblotted with anti-FLAG antibody. coIP with anti-EGFP antibody was conducted to confirm the expression of various plasmids. (E) Quantified results of (D) (F_4,15_ = 28.88, p < 0.001; q = 5.62, p = 0.001 comparing the AICDK43R+HDAC1 group with the AICDWT+HDAC1 group, and q = 4.09, p = 0.01 comparing the AICD-SUMO1+HDAC1 group with the AICDWT+HDAC1 group). (F) Different EGFP-tagged AICD plasmids and V5-Fe65 plasmid were co-transfected into Neuro2A cells and the expression level of NEP and TTR was determined by western blot. Immunoprecipitation and immunoblotting with anti-EGFP antibody was used to confirm the expression of various EGFP-tagged plasmid transfections. Immunoblotting with anti-V5 antibody was used to confirm the expression of V5-Fe65 transfection. (G) Quantified results of (F) (F_4,15_ = 128.56 for NEP and F_4,15_ = 135.15 for TTR, both p < 0.001). (H) The same plasmids were transfected into Neuro2A cells as described in (F) except that the V5-Fe65 plasmid was replaced with the FLAG-HDAC1 plasmid and immunoblotting with the anti-V5 antibody was replaced with the anti-FLAG antibody to confirm the expression of FLAG-HDAC1 transfection. (I) Quantified results of (H) (F_4,20_ = 141.48 for NEP and F_4,20_ = 199.41 for TTR, both p < 0.001). Experiments are in four repeats for (A), (D), and (F), and five repeats for (H). Data are expressed as individual values and mean ± SEM ∗∗p < 0.01, ^#^p < 0.001.

As mentioned above, AICD is stabilized by Fe65 and translocates to the nucleus, and it forms a complex with Fe65 and Tip60 for transcriptional activation.^3^^,^^4^ However, the relationship between AICD and Fe65 upon AICD SUMOylation is not known. To address this issue, different EGFP-AICD plasmids and V5-Fe65 plasmid were transfected into HEK293T cells. The cell lysates were immunoprecipitated with anti-EGFP antibody and immunoblotted with anti-V5 antibody. The results revealed that AICD is associated with Fe65, but that this association is diminished by EGFP-AICDK43R mutant transfection and is enhanced by EGFP-AICD-SUMO1 fusion plasmid transfection (Figures 3A and 3B). Cell lysates were also immunoprecipitated and immunoblotted with anti-EGFP antibody to confirm the transfection and expression of various EGFP-AICD plasmids (Figure 3A, lower panel).

To further examine the relationship between AICD SUMOylation and Fe65, different EGFP-AICD plasmids and V5-Fe65 plasmid (conjugated with Cy3 secondary antibody) were co-transfected into Neuro2A cells and immunofluorescence staining against EGFP and Cy3 was visualized. DAPI was added to the reaction for nuclear staining. As shown in Figure 3C, when EGFP-AICDWT plasmid was transfected, AICD was observed in the nucleus (EGFP), and it was co-localized with DAPI staining (EGFP+DAPI). When EGFP-AICDWT plasmid and V5-Fe65 plasmid were co-transfected, Fe65 was also present in the nucleus (Cy3), and it was mostly co-localized with DAPI staining (Cy3+DAPI). The merged image further indicated that Fe65 was mostly co-localized with AICD in the nucleus with DAPI staining (EGFP+Cy3+DAPI). In addition, Fe65 and AICD were also co-localized in part of the cytosol area (EGFP+Cy3). Alternatively, co-transfection of EGFP-AICDK43R and V5-Fe65 plasmids prevented nuclear translocation of AICD (EGFP). The presence of Fe65 in the nucleus (Cy3) was also reduced compared with the EGFP-AICDWT+V5-Fe65 transfection. Co-localization of AICD and Fe65 with DAPI (EGFP+Cy3+DAPI) was similarly diminished in the nucleus; instead, co-localization of AICD and Fe65 was observed in the cytosol area. This distribution pattern changed dramatically when the SUMO form of AICD plasmid (EGFP-AICD-SUMO1) was co-transfected with V5-Fe65 plasmid. Both AICD and Fe65 were consistently present in the nucleus, but the morphology of AICD staining was changed (EGFP), with AICD not co-localized with DAPI staining (EGFP+DAPI). Furthermore, AICD showed good co-localization with Fe65, and together they formed nuclear puncta (EGFP+Cy3), but they did not co-localize with DAPI staining (EGFP+Cy3+DAPI). Instead, these nuclear puncta were located in the perichromatin region close to the area stained with DAPI. In addition, Fe65 staining was exclusively observed in the nucleus when the SUMO form of AICD was transfected into the cell. This is probably because when AICD is SUMOylated, Fe65 preferentially binds AICD in the nucleus and is unable to translocate to the cytosol area. This result is congruent with the previous finding that AICD forms a complex with Fe65 and Tip60 in the nucleus for transcriptional activation,^3^^,^^4^ and our finding that SUMOylated AICD shows a stronger interaction with Fe65 than does WT AICD (Figure 3A).

A previous study showed that binding of HDAC1 to the *NEP* promoter and *TTR* promoter was decreased in neuroblastoma cells expressing the APP695 isoform.^14^ This is probably because more AICD is generated by β- and γ-secretase cleavage of the APP695 protein and because AICD competes with HDAC1 for binding to the *NEP* and *TTR* promoters.^14^ In this experiment, we examined whether AICD is associated with HDAC1 and whether this association is altered by AICD SUMOylation. Different EGFP-tagged AICD plasmids and FLAG-HDAC1 plasmid were transfected into HEK293T cells. The cell lysates were immunoprecipitated with anti-EGFP antibody and immunoblotted with anti-FLAG antibody. The results revealed that AICD interacts with HDAC1. This interaction is increased by EGFP-AICDK43R mutant transfection and is diminished by EGFP-AICD-SUMO1 fusion plasmid transfection (Figures 3D and 3E). Cell lysates were also immunoprecipitated and immunoblotted with anti-EGFP antibody to confirm the transfection and expression of various EGFP-tagged plasmids (Figure 3D, lower panel).

After showing the association between AICD and Fe65, we next examined how the interaction between AICD and Fe65 affects AICD-mediated transcriptional regulation of NEP and TTR expression in the context of AICD SUMOylation. Different EGFP-tagged AICD plasmids and V5-Fe65 plasmid were co-transfected into Neuro2A cells, and the cell lysates were subjected to western blot analysis of NEP and TTR expression. The results indicated that overexpression of AICD increased the expression levels of both NEP and TTR compared with the control group, with this effect enhanced by Fe65 co-expression. However, co-transfection of EGFP-AICDK43R and V5-Fe65 reversed the effect of AICD on NEP and TTR expression, but NEP and TTR expression levels were rescued and further increased by co-transfection of EGFP-AICD-SUMO1 fusion plasmid and V5-Fe65 plasmid (Figures 3F and 3G). These results indicated that AICD and Fe65 interaction increased NEP and TTR expression and that this effect was further enhanced by AICD SUMOylation. Next, we similarly examined whether the interaction of AICD and HDAC1 affects AICD-mediated regulation of NEP and TTR expression in the context of AICD SUMOylation. Different EGFP-tagged AICD plasmids and FLAG-HDAC1 plasmid were co-transfected into Neuro2A cells and the cell lysates were subjected to western blot analysis of NEP and TTR expression. The results revealed that overexpression of AICD consistently increased the expression levels of both NEP and TTR compared with the control group, but that this effect was diminished by HDAC1 co-expression. Moreover, NEP and TTR expression levels were more substantially decreased when EGFP-AICDK43R plasmid and FLAG-HDAC1 plasmid were co-transfected. However, they were significantly rescued by co-transfection of the EGFP-AICD-SUMO1 fusion plasmid and FLAG-HDAC1 plasmid (Figures 3H and 3I). These results indicate that HDAC1 competes with AICD in AICD-mediated regulation of NEP and TTR expression, and that SUMOylated AICD produces a stronger effect than does WT AICD in these regulations.

### AICD SUMOylation Increases AICD Association with CREB and p65, and It Increases CREB Binding to the *NEP* Promoter and p65 Binding to the *TTR* Promoter

**Figure 4:**
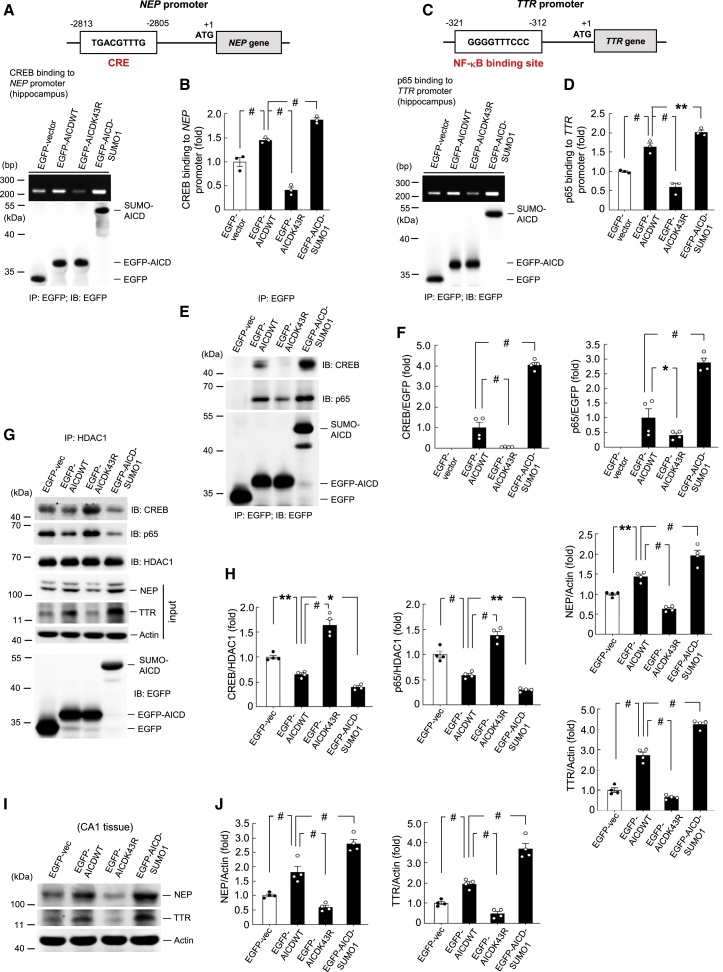
AICD SUMOylation Increases AICD Association with CREB and p65 and Increases CREB Binding to the *NEP* Promoter and p65 Binding to the *TTR* Promoter (A) The position of the CRE element on the *NEP* gene promoter is shown. Different EGFP-AICD plasmids were transfected into the rat CA1 area, and a ChIP assay for CREB binding to the *NEP* promoter in the hippocampus is shown. coIP using anti-EGFP antibody was conducted to confirm the expression of various AICD plasmids. (B) Quantified results of (A) (F_3,8_ = 106.72, p < 0.001). (C) The position of the NF-κB binding site on the *TTR* gene promoter is shown. Different EGFP-AICD plasmids were transfected into the rat CA1 area, and a ChIP assay for p65 binding to the *TTR* promoter in the hippocampus is shown. coIP using anti-EGFP antibody was conducted to confirm the expression of various AICD plasmids. (D) Quantified results of (C) (F_3,8_ = 74.87, p < 0.001). Experiments are in three repeats for (A) and (C). (E) Different EGFP-AICD plasmids were transfected into the rat CA1 area. Cell lysate was immunoprecipitated with anti-EGFP antibody and immunoblotted with anti-CREB and anti-p65 antibodies. Cell lysate was also immunoblotted with anti-EGFP antibody to confirm the transfection and expression of various AICD plasmids. (F) Quantified results of AICD association with CREB are shown in the left panel (F_3,12_ = 174.12, p < 0.001), and those for AICD association with p65 are shown in the right panel (F_3,12_ = 55.19, p < 0.001). Experiments are in four repeats for (E). (G) Different EGFP-tagged AICD plasmids were transfected into Neuro2A cells. The cell lysates were immunoprecipitated with anti-HDAC1 antibody and immunoblotted with anti-CREB as well as anti-p65 antibodies. Cells lysates were also subjected to western blot determination of NEP and TTR expression. Western blotting with anti-EGFP antibody was conducted to confirm the transfection and expression of various AICD plasmids. (H) Quantified results of (G) are shown (F_3,12_ = 80.03 for CREB/HDAC1 and F_3,12_ = 82.55 for p65/HDAC1; F_3,12_ = 66.32 for NEP and F_3,12_ = 215.51 for TTR, all p < 0.001). (I) The same cell lysates from (E) were used for the determination of NEP and TTR expression (n = 4). (J) The quantified results of NEP (F_3,12_ = 58.78, p < 0.001) and TTR (F_3,12_ = 77.29, p < 0.001) expression are shown. Data are expressed as individual values and mean ± SEM. ∗p < 0.05, ∗∗p < 0.01, ^#^p < 0.001.

The above results from immunofluorescence staining and coIP experiments indicate that when AICD is SUMOylated, the association between AICD and Fe65 is increased, presumably for the purpose of transcriptional regulation; however, whether the association between SUMOylated AICD and specific transcription factors is also increased remains unclear. To investigate this issue, we first examined the promoter sequence of the *NEP* gene and found that the *NEP* promoter contains the CRE element specific for CREB binding (Figure 4A, upper panel). We then transfected different EGFP-tagged AICD plasmids to the rat CA1 area and carried out a chromatin immunoprecipitation (ChIP) assay. The results revealed that transfection of EGFP-AICD increases CREB binding to the *NEP* promoter compared with the control group. The binding intensity was decreased by EGFP-AICDK43R transfection and enhanced by EGFP-AICD-SUMO1 transfection (Figures 4A and 4B). We also analyzed the *TTR* promoter, finding that it contains the nuclear factor κB (NF-κB) binding element specific for p65 (a subunit of NF-κB) binding (Figure 4C, upper panel). We similarly transfected different EGFP-tagged AICD plasmids to the rat CA1 area and carried out a ChIP assay. The results revealed that transfection of EGFP-AICD increases p65 binding to the *TTR* promoter compared with the control group. The binding intensity was decreased by EGFP-AICDK43R transfection and increased by EGFP-AICD-SUMO1 transfection (Figures 4C and 4D). Next, we carried out coIP experiments to examine the association between AICD and CREB as well as that between AICD and p65. Different EGFP-tagged AICD plasmids were transfected into the rat CA1 area. The cell lysates were immunoprecipitated with anti-EGFP antibody and immunoblotted with anti-CREB and anti-p65 antibody. The results revealed that AICD is associated with both CREB and p65. These associations are apparently reduced by EGFP-AICDK43R transfection and are markedly enhanced by EGFP-AICD-SUMO1 transfection (Figures 4E and 4F). Cell lysates were also immunoprecipitated and immunoblotted with anti-EGFP antibody to confirm the transfection and expression of various EGFP-tagged plasmids (Figure 4E, lower panel).

Next, we examined the contribution of HDAC1 in AICD- and AICD SUMOylation-induced NEP and TTR expression in the context of CREB- and p65-mediated transcriptional regulation. In analyzing HDAC1 and CREB interaction as well as HDAC1 and p65 interaction in response to AICD SUMOylation, different EGFP-tagged AICD plasmids were transfected into Neuro2A cells. Cell lysates were immunoprecipitated with anti-HDAC1 antibody and immunoblotted with anti-CREB, anti-p65, and anti-HDAC1 antibodies. The results revealed that the interaction between HDAC1 and CREB decreases upon EGFP-AICDWT transfection compared with the control group. It is further decreased upon EGFP-AICD-SUMO1 transfection, but it is markedly increased by EGFP-AICDK43R transfection (Figures 4G and 4H, left and middle panels). We also analyzed NEP and TTR expression from the same cell lysates in the same context. The results indicated that overexpression of EGFP-AICDWT increased both NEP and TTR expression compared with the control group. This effect was further enhanced by EGFP-AICD-SUMO1 transfection, but was significantly diminished by EGFP-AICDK43R transfection (Figures 4G and 4H, right panel). Taken together, these results revealed that AICD SUMOylation decreased the association between HDAC1 and CREB as well as between HDAC1 and p65, resulting in increased NEP and TTR expression.

Because the *NEP* promoter contains the CRE element for CREB binding and the *TTR* promoter contains the NF-κB binding site for p65 binding, and transfection of the SUMO form of AICD increases the association between AICD and CREB as well as that between AICD and p65, we next examined whether SUMOylated AICD indeed increases the expression of NEP and TTR in the hippocampus. The same cell lysates from Figure 4E were subjected to western blot analysis of NEP and TTR expression. The results showed that transfection of EGFP-AICDWT increased, whereas transfection of EGFP-AICDK43R decreased, the expression of both NEP and TTR in the hippocampus. However, transfection of EGFP-AICD-SUMO1 plasmid more substantially increased NEP and TTR expression compared with EGFP-AICDWT transfection (Figures 4I and 4J). Above we showed that AICD and SUMOylated AICD compete with HDAC1 in the regulation of NEP and TTR expression in Neuro2A cells (Figures 3H and 3I). The present results further indicate that AICD is sufficient to drive the expression of NEP and TTR in hippocampal neurons in the absence of HDAC1 and that this effect is enhanced by AICD SUMOylation.

### Lentiviral AICD-SUMO1 Transduction Decreases the Amount of Aβ and Amyloid Plaques and Rescues Spatial Memory Impairment in APP/PS1 Mice

**Figure 5:**
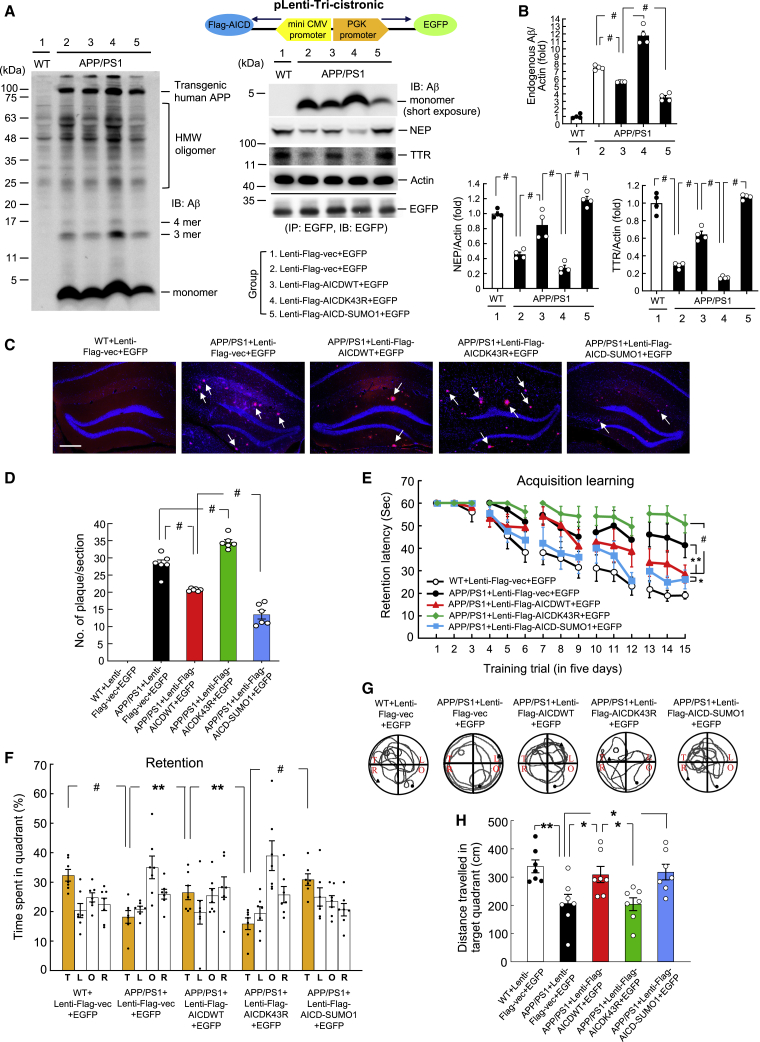
Lentiviral AICD-SUMO1 Transduction Decreases the Amount of Aβ and Amyloid Plaques and Rescues Spatial Memory Impairment in APP/PS1 Mice (A) Co-expression vectors containing different lenti-FLAG-AICDs and EGFP were transduced into the hippocampus of each APP/PS1 mouse (8–9 months old), and the amounts of Aβ and Aβ oligomers and NEP and TTR expression were examined by western blot 2 weeks later (n = 4). The construct of the lenti-FLAG-AICD and EGFP co-expression vector is shown in the upper-right panel. The EGFP bands confirm the transduction and expression of these lenti-vectors. The descriptions of the various lenti-FLAG-AICD and EGFP co-expression vectors that were transduced into each APP/PS1 mouse are shown in the lower-right panel. HMW, high molecular weight. (B) Quantified results of (A) (F_4,15_ = 183.67, p < 0.001 for endogenous Aβ, F_4,15_ = 52.69, p < 0.001 for NEP, and F_4,15_ = 161.99, p < 0.001 for TTR). The significance levels of separate sets of comparisons are shown in the figure. (C) A different batch of APP/PS1 mice received the same lenti-FLAG-AICD and EGFP co-expression vector transductions as described above, and amyloid plaque deposits (red) examined by Proteostat dye staining are shown in the hippocampus 2 weeks later. DAPI staining is shown in blue (n = 3, two tissue slices for each animal). Scale bar represents 200 μm. (D) Quantified results of (C) (F_3,20_ = 92.09, p < 0.001). The significance levels of separate sets of comparisons are shown in the figure. (E) Another batch of APP/PS1 mice (and WT mice) received the same lenti-FLAG-AICD and EGFP co-expression vector transductions as described above, and they were subjected to spatial learning 2 weeks later (n = 7). Their acquisition performance is shown (F_4,30_ = 30.66, p < 0.001). (F) Retention performance (probe trial) of time spent in the target quadrant of these animals (for target region, F_4,30_ = 12.34, p < 0.001). (G) Representative swim patterns from the probe trial test of each group. (H) Distance travelled in the target quadrant for the probe trial test of these animals (F_4,30_ = 5.78, p < 0.01). Data are expressed as individual values and mean ± SEM. For (E), data are expressed as mean ± SEM. ∗p < 0.05, ∗∗p < 0.01, ^#^p < 0.001.

The above results showed that SUMOylation of AICD increases its association with CREB and p65 and enhances the expression of NEP and TTR. Because NEP and TTR both degrade Aβ, we expected that AICD SUMOylation would decrease the amount of Aβ and amyloid plaques. To examine this issue, co-expression vectors containing different lentivirus (lenti-)FLAG-tagged AICD (or lenti-FLAG) and EGFP were transduced into the hippocampus of APP/PS1 mice (8–9 months old) and the Aβ level was determined by western blotting 2 weeks later. The construct of the lenti-FLAG-AICD and EGFP co-expression vector is shown in the upper-right panel of Figure 5A. The results indicated that Aβ was present in the hippocampus of APP/PS1 mice, but not in WT mice. Transduction of the lenti-FLAG-AICDWT vector to APP/PS1 mice reduced the amount of Aβ, but transduction of the lenti-FLAG-AICDK43R vector to APP/PS1 mice increased the amount of Aβ compared with APP/PS1 mice receiving lenti-FLAG-vector transduction. Moreover, transduction of the lenti-FLAG-AICD-SUMO1 vector to APP/PS1 mice further decreased the amount of Aβ compared with APP/PS1 mice receiving lenti-FLAG-AICDWT vector transduction (Figures 5A and 5B, upper panel). Because Aβ can be degraded by NEP and TTR, we also determined NEP and TTR levels in these animals. Western blotting results revealed that the NEP level was decreased in APP/PS1 mice compared with WT mice. This reduction was significantly rescued in APP/PS1 mice receiving lenti-FLAG-AICDWT transduction, but worsened in APP/PS1 mice receiving lenti-FLAG-AICDK43R transduction. However, NEP expression was completely rescued in APP/PS1 mice receiving lenti-FLAG-AICD-SUMO1 transduction. Similar results were also found for TTR expression (Figures 5A and 5B, lower panel). Next, we examined whether AICD SUMOylation produces a similar effect on amyloid plaque deposits. The same lenti-FLAG-AICD vectors as those used above were transduced into a different batch of APP/PS1 mice (8–9 months old), and amyloid plaques were examined using Proteostat dye staining 2 weeks later. The results obtained were similar to those for Aβ accumulation. Apparent amyloid plaque deposits were observed in APP/PS1 mice (indicated by arrows), but not in WT mice. This was reduced by lenti-FLAG-AICDWT transduction but was increased by lenti-FLAG-AICDK43R transduction. Lenti-FLAG-AICD-SUMO1 transduction further decreased the amount of amyloid plaques compared with lenti-FLAG-AICDWT transduction (Figures 5C and 5D).

Next, we examined the functional significance of AICD SUMOylation. To address this issue, we similarly transduced different lenti-FLAG-AICD vectors into APP/PS1 mice (8–9 months old) and, 2 weeks later, subjected the mice to a water maze learning test. The results revealed that APP/PS1 mice showed significant impairment of acquisition learning compared with WT mice. This impairment was partially rescued by lenti-FLAG-AICDWT transduction, but was worsened by lenti-FLAG-AICDK43R transduction. However, transduction of lenti-FLAG-AICD-SUMO1 vector to APP/PS1 mice further rescued this impairment compared with APP/PS1 mice receiving lenti-FLAG-AICDWT transduction (Figure 5E). Retention performance (probe trial) was assessed in these animals 24 h later. Results for the retention measures “time spent in the target quadrant” (Figure 5F) and “distance travelled in the target quadrant” (Figure 5H) of these animals paralleled the results of their acquisition learning performance (Figure 5E). However, the swim speeds of these animals were similar (Figure S2). Representative swim patterns from the probe trial test for each group of mice are shown in Figure 5G.

### Melatonin Increases AICD SUMOylation and Increases Expression of PIAS1, NEP, and TTR in Rats

**Figure 6:**
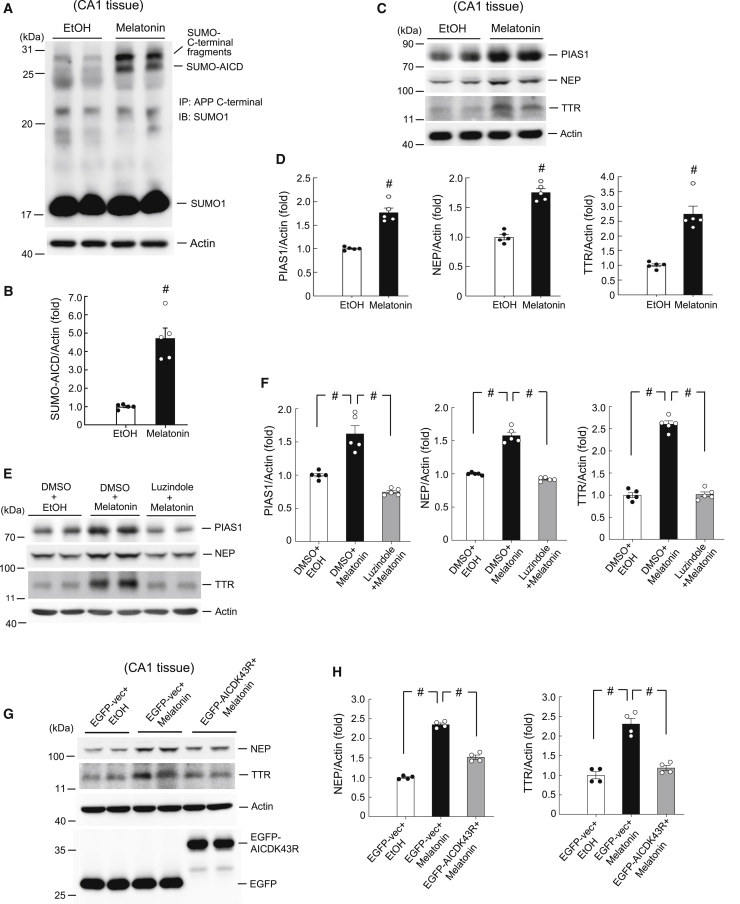
Melatonin Increases PIAS1 Expression and AICD SUMOylation and Increases the Expression of NEP and TTR in Rats (A) Rats were divided into two groups (n = 5), one receiving ethanol (20%) and the other melatonin (7 μg) injected into the CA1 area. They were sacrificed 1 h later and the AICD SUMOylation level was determined. (B) Quantified results of (A) (t_1,8_ = 6.7, p < 0.001). (C) Tissue lysates from the same animals were subjected to western blot analysis for the expression of PIAS1, NEP, and TTR. (D) Quantified results of (C) (t_1,8_ = 8.12, p < 0.001 for PIAS1, t_1,8_ = 9.77, p < 0.001 for NEP, and t_1,8_ = 6.52, p < 0.001 for TTR). (E) Another batch of rats was divided into three groups (n = 5) and received DMSO (40%)+ethanol (20%), DMSO+melatonin (7 μg), or luzindole (1 μg)+melatonin (7 μg) injections to their CA1 area. They were sacrificed 1 h after the second injection and their CA1 tissue was subjected to western blot determination of PIAS1, NEP, and TTR expression. (F) Quantified results of (E) (F_2,12_ = 38.2 for PIAS1, F_2,12_ = 141.43 for NEP, and F_2,12_ = 227.9 for TTR, all p < 0.001). (G) Rats were divided into three groups (n = 4) and received EGFP-vector transfection+ethanol (20%) injection, EGFP-vector transfection+melatonin injection (7 μg), and EGFP-AICDK43R transfection+melatonin injection (7 μg) into the CA1 area. The interval between these two injections was 24 h. They were sacrificed 1 h after melatonin (or ethanol) injection, and their CA1 tissue was subjected to western blot determination of NEP and TTR expression. (H) Quantified results of (G) (F_2,9_ = 271.43, p < 0.001 for NEP and F_2,9_ = 43.8, p < 0.001 for TTR). Data are expressed as individual values and mean ± SEM. ^#^p < 0.001. EtOH, ethanol.

In this experiment, we aimed to identify an endogenous stimulus that regulates AICD SUMOylation. Melatonin is a pineal hormone whose levels are high in individuals during puberty and decline in aged people, suggesting that it may play a role in aging-related neurodegenerative diseases.^26^ More closely related to the present study, melatonin levels have been shown to be lower in AD patients than in age-matched controls.^27^ Furthermore, the progression of AD pathology is paralleled by a decline in cerebrospinal fluid melatonin levels.^28^ Alternatively, studies of transgenic animal models of AD have suggested that melatonin alleviates the pathology of AD and increases survival.^29^^,^^30^ In this study, we examined whether melatonin exerts its protective effect against AD through enhanced SUMOylation of AICD. Rats were divided into two groups, one receiving ethanol and the other melatonin infusion into the hippocampal CA1 area. They were sacrificed 1 h after infusion and their CA1 tissue was dissected out and subjected to AICD SUMOylation determination. The results revealed that acute melatonin injection significantly increased the level of AICD SUMOylation (Figures 6A and 6B). Melatonin also apparently increased the SUMOylation of APP C-terminal fragments (Figure 6A). Given our results presented above, showing that AICD is SUMO-modified by PIAS1 and that AICD SUMOylation increases NEP and TTR expression, we speculated that melatonin infusion should also increase the expression of PIAS1, NEP, and TTR. To examine this issue, cell lysates from the same animals were subjected to western blot determination of these three proteins. The results revealed that melatonin infusion markedly increased the expression levels of PIAS1, NEP, and TTR (Figures 6C and 6D). We also examined whether melatonin receptor agonists produce the same effect. Rats were randomly divided into two groups, one receiving DMSO and the other agomelatine injection to the hippocampal CA1 area, and the expression levels of PIAS1, NEP, and TTR were determined by western blotting 1 h later. The results revealed that, similar to what was observed for melatonin, agomelatine increased the expression levels of PIAS1, NEP, and TTR (Figures S3A and S3B). Next, we investigated the mechanism mediating the acute effect of melatonin on these measures. First, we examined whether these effects are mediated through melatonin receptors. A separate batch of rats was randomly divided into three groups receiving DMSO+ethanol (EtOH), DMSO+melatonin, and luzindole+melatonin injections, respectively, into their CA1 area. The two injections were separated by 45 min. The expression levels of PIAS1, NEP, and TTR were determined by western blotting 1 h after the second injection. The results revealed that melatonin injection consistently increased the levels of PIAS1, NEP, and TTR, but that these increases were completely blocked by prior injection of the melatonin receptor antagonist luzindole (Figures 6E and 6F). We further investigated the melatonin receptor-mediated neuronal signaling suggested by these findings. Based on previous results showing that melatonin alleviates AD through enhanced nonamyloidogenic processing of APP and ADAM10 expression via activation of the mitogen-activated protein kinase (MAPK)/extracellular signal-regulated kinase (ERK) signaling pathway,^30^ we examined whether MAPK/ERK activation also mediates the effect of melatonin on PIAS1 expression. Rats were randomly divided into three groups, receiving DMSO+ethanol, DMSO+melatonin, and U0126+melatonin injections, respectively, to the CA1 area. The interval between the two injections was 30 min. Animals were sacrificed 1 h after the second injection, and their CA1 tissue was subjected to western blot determination of PIAS1, phosphorylated (p-)ERK1/2, and ERK1/2 expression. The results revealed that acute melatonin injection consistently increased PIAS1 expression and markedly increased the phosphorylation level of ERK1/2, with both of these effects being blocked by prior administration of the MAPK kinase (MEK) inhibitor U0126, which prevents the activation of ERK1/2 (Figures S4A and S4B). The ERK1/2 expression level remained unchanged. These results indicate that melatonin receptor-mediated MAPK/ERK neuronal signaling plays a critical role in PIAS1 expression and in counteracting Aβ toxicity. Lastly, we addressed the issue of whether AICD SUMOylation mediates the effect of melatonin on NEP and TTR expression. Rats were divided into three groups, receiving EGFP-vector transfection+PBS injection, EGFP-vector transfection+melatonin injection, and EGFP-AICDK43R transfection+melatonin injection, respectively, to the CA1 area. Rats were sacrificed 1 h after the second injection and their CA1 tissue was subjected to western blot determination of NEP and TTR expression. The results revealed that melatonin consistently increased the expression of NEP and TTR, but blockade of AICD SUMOylation suppressed the effect of melatonin on NEP and TTR expression (Figures 6G and 6H).

### Melatonin Rescues Reduction in AICD SUMOylation, PIAS1, NEP, and TTR Expression in APP/PS1 Mice

**Figure 7:**
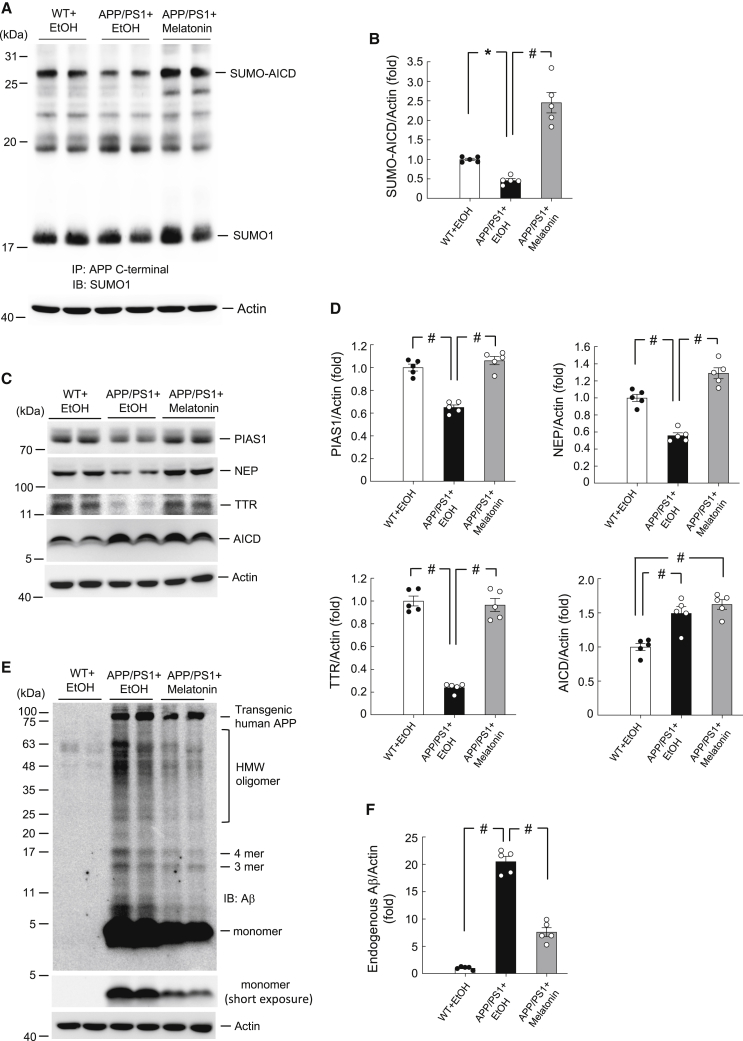
Melatonin Rescues Reduction in AICD SUMOylation, PIAS1, NEP, and TTR Expression in APP/PS1 Mice (A) Three groups of mice (n = 5) received daily ethanol (20%) or melatonin injections (0.03 ml, i.p.) for 3 weeks: wild-type mice receiving ethanol, APP/PS1 mice receiving ethanol, and APP/PS1 mice receiving melatonin. Mice were sacrificed 3 days after the last injection and their frontal cortex tissue was subjected to AICD SUMOylation determination. (B) Quantified results of (A) (F_2,12_ = 44.6, p < 0.001). (C) The hippocampal tissue from the same animals was subjected to western blot determination of PIAS1, NEP, TTR, and AICD levels. (D) Quantified results of (C) (F_2,12_ = 54.24 for PIAS1, F_2,12_ = 59.67 for NEP, F_2,12_ = 101.1 for TTR, and F_2,12_ = 18.53 for AICD, all p < 0.001). (E) The same cell lysates used in (C) were subjected to endogenous Aβ (monomer and oligomers) level determination using anti-Aβ antibody by western blot. (F) Quantified results of (E) (F_2,12_ = 188.04, p < 0.001). Data are expressed as individual values and mean ± SEM. ∗p < 0.05, ^#^p < 0.001.

In this series of experiments, we examined whether endogenous AICD SUMOylation, PIAS1, NEP, and TTR expression are lower in APP/PS1 mice than in WT mice, and whether melatonin treatment could rescue these deficits. Melatonin or ethanol was directly injected into the CA1 area of mice. For the first experiment, three groups of mice were used: WT mice receiving ethanol injection, APP/PS1 mice receiving ethanol injection, and APP/PS1 mice receiving melatonin injection. Melatonin or ethanol was administered intraperitoneally (i.p.) once per day for 3 weeks. Mice were sacrificed 3 days after the last injection, the frontal cortex tissue was subjected to AICD SUMOylation determination, and hippocampal tissue was subjected to western blot determination of PIAS1, NEP, and TTR expression, as well as Aβ and AICD levels. The results revealed that AICD SUMOylation was significantly decreased in APP/PS1 mice treated with sub-chronic ethanol compared to WT mice treated with sub-chronic ethanol, but sub-chronic melatonin injection completely rescued this deficit (Figures 7A and 7B). Similar results were found with endogenous PIAS1, NEP, and TTR expression in APP/PS1 mice and WT mice treated with sub-chronic ethanol and in APP/PS1 mice treated with sub-chronic melatonin (Figures 7C and 7D). However, the endogenous AICD expression level was increased in both APP/PS1 mice treated with sub-chronic ethanol and APP/PS1 mice treated with sub-chronic melatonin (Figures 7C and 7D).

Because the above results showed that melatonin rescues the reduced NEP and TTR expression in APP/PS1 mice and because NEP and TTR both degrade Aβ, we next asked whether melatonin treatment could reduce Aβ levels in APP/PS1 mice. The frontal cortex tissue from the same animals as described above was used to determine the endogenous Aβ level by western blotting. The results revealed that the levels of both Aβ monomer and oligomers were significantly higher in APP/PS1 mice compared with WT mice, but sub-chronic melatonin injection dramatically decreased the levels of both Aβ monomer and oligomers in APP/PS1 mice (Figures 7E and 7F).

## Discussion

In the present study of the role of AICD SUMOylation in the context of AD, we found that AICD SUMOylation functions as an endogenous protection mechanism against Aβ toxicity in APP/PS1 mice and mediates the neuroprotective effect of melatonin against AD through enhanced degradation of Aβ. In addition, we found that AICD is SUMO-modified by PIAS1 in both HEK293T cells and the brain. Multiple, although in some cases indistinct, AICD-SUMO bands were observed in cells, with AICD SUMOylation at Lys-43 giving rise to the most prominent band. This result suggests that there may be other SUMO residues on AICD in cells. Whether mutations of these residues have an additive effect on blockade of AICD SUMOylation and NEP and TTR expression requires further investigation. Alternatively, Lys-43 seems to be the predominant, if not the only, SUMO residue on AICD in the hippocampus, and mutation at Lys-43 completely abolished the biochemical, physiological, and behavioral effects of AICD SUMOylation. The difference regarding candidate AICD SUMO residues between cell lines and the brain also needs to be clarified. Our results showed that AICD is co-localized with PIAS1 and SUMO-modified by PIAS1 endogenously in hippocampal neurons. Our results further revealed that PIAS1 is also associated with the C83 and C99 fragments, in addition to AICD, and that these fragments are endogenously SUMO-modified by PIAS1 as well. This result suggests that PIAS1 SUMOylates both the cleaved AICD and the un-cleaved AICD present in the C83 and C99 fragments, but that PIAS1 SUMOylation of the cleaved AICD takes place in the nucleus because endogenous PIAS1 and AICD are co-localized only in the nucleus (Figure 2D). However, it is unlikely that the C83 and C99 fragments (excluding the AICD fragment) are direct SUMO substrates of PIAS1 because immunohistochemical studies have shown that both the C83 and C99 fragments are localized on the cell membrane.^31^ The observed association between PIAS1 and C83/C99 fragments likely arose because, when the coIP experiments were carried out, the cells were disrupted and some PIAS1 moved to the extranuclear area. As a result, PIAS1 is associated with, and SUMO modifies, AICD present on the C83 and C99 fragments in the cytosol area. However, the PIAS1-C83 fragment interaction probably does not affect the nonamyloidogenic processing of APP in the context of AICD SUMOylation because the C83 fragment is a product of α-secretase cleavage of APP; hence, the presence of the C83 fragment per se is a result of the nonamyloidogenic processing of APP, whether it interacts with PIAS1 or not. The physiological significance of the endogenous association between PIAS1 and C83/C99 fragments containing AICD warrants further exploration in future studies. Moreover, the less intense association between PIAS1 and AICD than between PIAS1 and the C83 and C99 fragments is congruent with a previous report showing that AICD is unstable and easily degraded.^6^ Furthermore, there is a stronger association between PIAS1 and the C83 fragment than between PIAS1 and the C99 fragment (Figure 2A). This is consistent with the general notion that the nonamyloidogenic pathway is the predominant pathway under physiological conditions. Alternatively, it is unlikely that PIAS1 is associated with Aβ40/42 or the P3 peptide on the C83 and C99 fragments, respectively, because no band with a molecular mass less than 6 kDa (the size of AICD) was observed (Figure 2A).

In an examination of the molecular mechanism of AICD SUMOylation, we found that the association between AICD and Fe65 is increased when AICD is SUMOylated, but that this association is reduced when AICD SUMOylation at Lys-43 is blocked. Furthermore, immunofluorescence staining revealed that SUMOylated AICD was well co-localized with Fe65 in the nucleus only, but that blockade of AICD SUMOylation prevented nuclear translocation of AICD. These findings are likely due to the fact that Lys-43 is located immediately next to the YENPTY motif on AICD, which is the binding domain for Fe65^32^ that is necessary for the stabilization and subsequent nuclear translocation of AICD.^3^^,^^4^ When AICD is SUMOylated, its association with Fe65 is increased (Figure 3A) and nuclear localization of AICD is observed. On the contrary, mutation at Lys-43 may cause a conformational change of AICD that prevents Fe65 binding to AICD and subsequent AICD nuclear translocation. This suggestion is partly supported by our finding that mutation at Lys-43 reduces the stability of AICD (Figure 1E). It is also possible that AICD SUMOylation changes the interaction between AICD and Fe65, given previous results showing that SUMOylation alters protein-protein interactions.^33^ Moreover, immunofluorescence experiments revealed that SUMOylated AICD and Fe65 co-localized as puncta in the perichromatin region of Neuro2A neurons. Because the perichromatin region has important functions including transcription,^34^ this distribution allows the AICD complex to interact with transcription factors for transcriptional regulation.

In a further study of the mechanism of AICD SUMOylation, we also demonstrated that SUMOylated AICD shows weaker interaction with HDAC1, stronger interaction with CREB and p65, enhanced binding to the *NEP* and *TTR* promoters, and increased expression of NEP and TTR. Conversely, blockade of AICD SUMOylation had opposite effects. These results provide direct evidence supporting the previous speculation that AICD competes with HDAC1 for DNA binding in cells overexpressing APP695.^14^ Moreover, our results further demonstrate that SUMOylation of AICD plays a critical role in competing with HDAC1 for interaction with CREB and p65 for transcriptional regulation of NEP and TTR expression. Similarly, in another study we found that SUMOylation of the methyl-CpG-binding protein 2 protein releases CREB from the HDAC1 co-repressor complex for upregulation of brain-derived neurotrophic factor expression.^35^ In the present study, an examination of the physiological significance of AICD SUMOylation disclosed that transduction of lenti-FLAG-AICDWT vector to the hippocampus of APP/PS1 mice decreases the amount of Aβ and amyloid plaques and rescues spatial memory deficit in APP/PS1 mice. This result is consistent with reports that AICD plays a protective role against AD.^6^^,^^9^^,^^14^ However, our results are not congruent with reports that AICD is involved in the pathology of AD. For example, AICD59 transgenic mice show tau hyperphosphorylation and impaired working memory.^36^ Furthermore, overexpression of AICD57 and AICD59 in PC12 cells was found to induce GSK-3β expression and phosphorylation and produce neurotoxicity.^37^ This discrepancy is likely due, at least in part, to the different lengths of the AICD fragments used in these studies. In the present study we used AICD50, which can translocate to the nucleus to regulate gene expression, whereas nuclear translocation of AICD57 and AICD59 has not been reported, and these AICDs may produce their effects in the cytosol area. In addition, we found that, compared with WT AICD, SUMOylated AICD exerts greater effects in reducing Aβ level and amyloid plaque accumulation and in rescuing the spatial memory deficit in APP/PS1 mice. This is probably due to enhanced expression of NEP and TTR by SUMOylated AICD, resulting in more efficient clearance of Aβ; therefore, less severe pathology and cognitive impairment were observed in APP/PS1 mice. This explanation is supported by our finding that blockade of AICD SUMOylation yields more Aβ and amyloid plaques, and it causes more severe impairment of spatial learning and memory, in APP/PS1 mice compared with APP/PS1 mice receiving lenti-FLAG-AICDWT transduction. A possible explanation for these results is that lower NEP and TTR expression leads to less degradation of Aβ, and hence to enhanced AD pathology in APP/PS1 mice. We also examined the effect of AICD SUMOylation on amyloid plaque accumulation in aged APP/PS1 mice (16 months old). The results indicated that AICD SUMOylation has a similar effect in reducing the number of amyloid plaques in aged APP/PS1 mice except that its effect is less dramatic than that observed in 8- to 9-month-old APP/PS1 mice (Figure S5). Our results are consistent with the notion that AICD is involved in nuclear signaling and transcriptional regulation in AD.^1^ They are also congruent with the literature indicating that SUMOylation plays a role in the pathogenesis of AD.^38^^,^^39^ Moreover, there is a report showing that APP can be ubiquitinated at Lys-688 (which corresponds to Lys-43 on AICD),^17^ but we found that mutation of AICD at Lys-43 did not affect AICD ubiquitination in the hippocampus (Figure 2H). This difference may be due to whether AICD ubiquitination occurs before or after AICD is cleaved from APP, but this has yet to be verified. There is also a study showing that neddylation occurs at Lys-31 and Lys-43 on AICD,^18^ but we found no apparent difference in AICD neddylation between the AICDWT group and AICDK43R group (Figure S6). This discrepancy is likely due to the fact that in the earlier study, both the Lys-31 and Lys-43 residues were mutated, whereas in our study only the Lys-43 residue was mutated. It could be that Lys-31 plays a more important role in AICD neddylation, although other possibilities exist.

**Figure 8:**
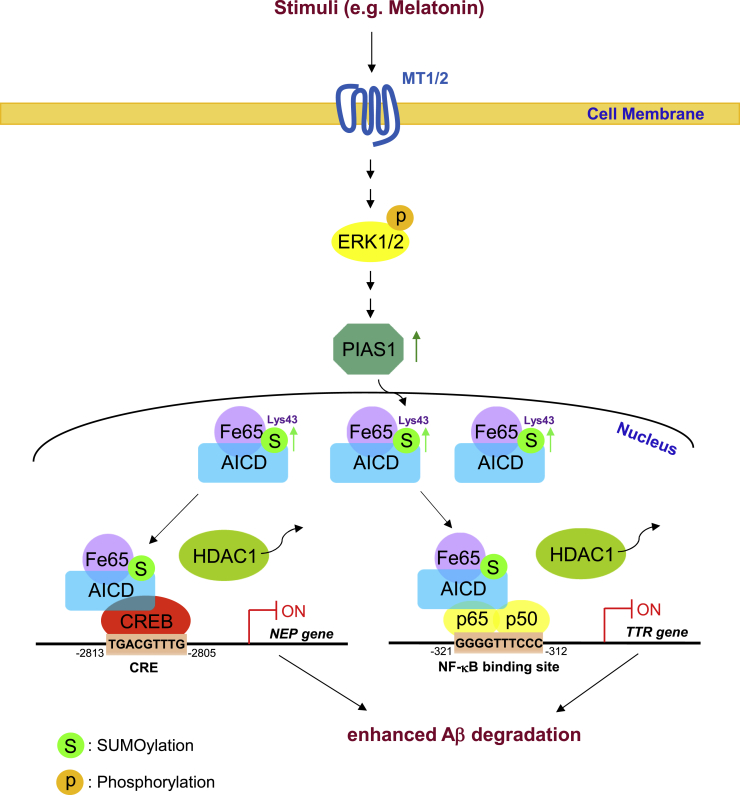
Melatonin Signaling in Protection against AD The illustration shows the relationship among AICD SUMOylation, AICD association with CREB and p65 in the AICD-Fe65 complex, its effect on CREB binding to the *NEP* gene promoter and p65 binding to the *TTR* gene promoter, as well as NEP and TTR expression for Aβ degradation. Melatonin is shown as an endogenous stimulus to induce AICD SUMOylation, and its neuronal signaling is also shown. MT1/2, melatonin receptor 1/2.

In an examination of the endogenous molecule that regulates AICD SUMOylation, we found that melatonin, a pineal gland neurohormone, increases the expression of PIAS1, enhances AICD SUMOylation, and increases the expression of NEP and TTR in the hippocampi of both rats and APP/PS1 mice. These effects would result in more efficient degradation of Aβ (Figure 8), consistent with the literature indicating that melatonin protects against AD.^29^^,^^30^ In addition, the AICD expression level is increased in both APP/PS1 mice and APP/PS1 mice treated with melatonin. This is an expected result because more AICD is generated in APP/PS1 mice due to amyloidogenic processing of APP by β-secretase and γ-secretase cleavage of APP. Although melatonin has been shown to promote nonamyloidogenic processing of APP,^40^ AICD is still generated in APP/PS1 mice treated with melatonin due to α-secretase and γ-secretase cleavage of APP. Furthermore, some evidence has suggested that APP-induced cell death is dependent on the cleavage to AICD. For example, overexpression of APP was found to induce cell death in the *Drosophila* nervous system, but expression of the truncated form of APP lacking the AICD domain failed to induce cell death.^41^ Another study showed that knockout of presenilins (PSs), part of the γ-secretase complex, reduces the expression and activity of p53, whereas overexpression of AICD in PS-deficient cells increases p53 reporter activity and p53 mRNA level.^42^ However, our results showed that AICD has a protective effect against Aβ toxicity. The difference between our results and the previous findings could be due to differences in the AICD-interacting proteins and AICD-regulated genes examined. We studied the effect of the interaction between AICD and Fe65 on the nuclear translocation of AICD, whereas the other study examined the interaction between AICD and FOXO that mediates apoptosis. Furthermore, we examined AICD-regulated NEP and TTR expression in terms of Aβ degradation, whereas the other study examined AICD-regulated p53 expression that mediates cell death.

The PIAS protein family has several members, but in the present study we concentrated on the role of PIAS1. We took this approach in part because of previous findings showing that PIAS1 expression is induced by neuronal activation,^43^ and also in light of previous work showing that PIAS1 SUMOylation of the MeCP2 protein alleviates symptoms of another neurological disorder, Rett syndrome.^35^ More closely related to the present study, we recently showed that PIAS1 can also SUMO-modify HDAC1 and Elk-1, and that PIAS1 SUMOylation of these two proteins alleviates pathological symptoms in an animal model of AD. However, PIAS1 SUMO-modifies the various proteins to rescue AD pathology via different, although somewhat overlapping, mechanisms. In the present study, we found that PIAS1 SUMOylation of AICD enhances Aβ degradation. In previous studies, however, PIAS1 SUMOylation of HDAC1 was found to reduce apoptosis,^22^ and PIAS1 SUMOylation of Elk-1 was found to promote neuronal survival in APP/PS1 mice.^23^ Furthermore, PIAS1 SUMOylation of AICD was strongly upregulated by melatonin, whereas melatonin is implicated in the prevention of AD. The present results further strengthen the importance of AICD SUMOylation as an endogenous prevention strategy against Aβ toxicity. Moreover, it is possible that other PIAS family proteins also SUMO-modify AICD. In addition, we cannot exclude the possibility that AICD could be SUMOylated by other SUMO E3 ligases, such as RanBP2.

In this study, we have demonstrated a novel posttranslational regulation of AICD with SUMO-modification and demonstrated that AICD SUMOylation is a novel defense mechanism protecting against AD pathology by facilitating the degradation of Aβ. We also identified a novel physiological role of melatonin in inducing AICD SUMOylation. These results shed light on a promising therapeutic direction to combat AD.

## Materials and Methods

### Animals

Adult male Sprague-Dawley rats (250–350 g) and C57BL/6 mice were purchased from the BioLASCO and the National Laboratory Animal Center, Taiwan, respectively. The APP/PS1 mice were purchased from Jackson Laboratory (Bar Harbor, ME, USA) (strain name B6.Cg-Tg(APPswe,PSEN1dE9)85Dbo/Mmjax, stock no. 005864). All animals were bred and maintained on a 12-h light/12-h dark cycle (light on at 8:00 am) at the Animal Facility of the Institute of Biomedical Sciences (IBMS), Academia Sinica with food and water continuously available. Experimental procedures follow the *Guide for the Care and Use of Laboratory Animals* of the National Institute of Health and were approved by the Animal Committee of IBMS, Academia Sinica.

### Plasmid DNA Construction

AICD is the product of γ-secretase cleavage of APP, and different lengths of AICD were identified. AICD50 and AICD51 are the major C-terminal fragments generated by γ-secretase.^44^ Furthermore, AICD50 is more stable than AICD51.^45^ We have therefore cloned *AICD50* for the present study. The details of plasmid construction for all genes are described in Supplemental Materials and Methods.

### Cell Culture and Plasmid Transfection

HEK293T cells and Neuro2A cells were maintained in Dulbecco’s modified Eagle’s medium containing 10% fetal bovine serum and incubated at 37°C in a humidified atmosphere with 5% CO_2_. Plasmid transfection was made by using the Lipofectamine 2000 reagent (Invitrogen, Carlsbad, CA, USA) in 6- and 12-well culture plates according to the manufacturer’s instructions. Immunoprecipitation (IP) and western blot were conducted 48 h after plasmid transfection.

### Plasmid DNA and siRNA Transfection to the Hippocampus

Rats were anesthetized with pentobarbital (40 mg/kg, i.p.) and subjected to stereotaxic surgery. EGFP-tagged AICD plasmid DNA was directly injected into the rat CA1 area at a rate of 0.1 μL/min. A total of 0.7 μL was injected into each side of the CA1 area. Plasmid DNA was prepared as described previously.^46^ Transient plasmid DNA transfection was conducted using the non-viral transfection agent polyethylenimine (PEI), and we have previously demonstrated that PEI does not produce toxicity to hippocampal neurons.^47^ Briefly, plasmid DNA was diluted in 5% glucose to a stock concentration of 2.77 μg/μL. Branched PEI of 25 kDa (Sigma) was diluted to 0.1 mM concentration in 5% glucose and added to the DNA solution. Immediately before injection, 0.1 mM PEI was added to reach a ratio of PEI nitrogen per DNA phosphate equal to 10. The mixture was subjected to vortexing for 30 s and allowed to equilibrate for 15 min. For siRNA injection, 0.7 μL of PIAS1 siRNA (10 pmol), CREB siRNA (10 pmol), or control siRNA was transfected into the rat CA1 area bilaterally also using the transfection agent PEI. The siRNA sequences are described in Supplemental Materials and Methods. The injection needle was left in place for 5 min to limit the diffusion of injected agent. Animals were sacrificed 48 h after plasmid transfection or siRNA injection and their hippocampal tissue was dissected out and subjected to coIP, western blot, and an *in vitro* SUMOylation assay.

### Lentiviral Vector Construction and Preparation

For construction of FLAG-AICD, FLAG-AICDK43R, and FLAG-AICD-SUMO1 lentiviral vectors, full-length FLAG-AICD, FLAG-AICDK43R, and FLAG-AICD-SUMO1 fusion plasmids were sub-cloned into the lentiviral vector pLenti-Tri-cistronic (ABM, Richmond, BC, Canada) by amplifying different FLAG-AICD non-viral constructs with different primes. The primer sequences and detailed procedures are described in Supplemental Materials and Methods. The EGFP sequence was cloned into the pLenti-vector, pLenti-FLAG-AICD, pLenti-FLAG-AICDK43R, and pLenti-FLAG-AICD-SUMO1 vectors to obtain a cistronic co-expressing vector. The primer sequences for EGFP and lentivirus packaging procedures are detailed in Supplemental Materials and Methods. The final concentration of the lentiviral vector used for injection to the brain is 5 × 10^8^ IU/mL.

### ChIP Assay

A ChIP assay was performed according to the protocol of the Millipore ChIP assay kit (catalog no. 17-10085). The ChIP assay was carried out in the rat hippocampus for determination of CREB binding to the *NEP* promoter and p65 binding to the *TTR* promoter upon various EGFP-tagged AICD plasmid transfections to rat CA1 area. The detailed procedures are described in Supplemental Materials and Methods.

### *In Vitro* SUMOylation Assay for the CA1 Tissue

Hippocampal CA1 tissue lysate was prepared in the same way as that prepared for western blot. For the IP experiment, the clarified lysate (0.5 mg) was immunoprecipitated with 3 μL of anti-EGFP antibody at 4°C overnight. The protein A agarose beads (30 mL, 50% slurry, GE Healthcare, IL, USA) were added to the IP reaction product to catch the immune complex at 4°C for 3 h. The immune complex on beads was washed three times with washing buffer containing 20 mM HEPES (pH 7.4), 150 mM NaCl, 1 mM EDTA, 1% IGEPAL CA-630, 1 mM DTT, 50 mM β-glycerophosphate, 50 mM NaF, 10 mg/mL PMSF, 4 mg/mL aprotinin, 4 mg/mL leupeptin, and 4 mg/mL pepstatin and subjected to *in vitro* SUMOylation reaction with the addition of the recombinant PIAS1 protein (3 μL, catalog no. BML-UW9960, Enzo Life Sciences, Farmingdale, NY, USA), E1 (1 μL), E2 (1 μL), and the SUMO1 (0.5 μL) proteins provided in the kit. An *in vitro* SUMOylation assay was performed using the SUMOlink kit according to the manufacturer’s instructions (Active Motif, CA, USA) and boiled in Laemmli sample buffer at 95°C for 10 min. The SUMOylation reaction product was subjected to 10% SDS-PAGE and transferred onto the polyvinylidene fluoride (PVDF) membrane. The membrane was immunoblotted with anti-SUMO1 antibody (1:3,000; Active Motif) or anti-EGFP antibody (1:8,000; Sigma-Aldrich, catalog no. 11814460001). For determination of endogenous AICD SUMOylation after PIAS1 siRNA transfection, the clarified lysate (0.5 mg) was immunoprecipitated with 3 μL of anti-APP C-terminal fragment antibody (BioLegend, San Diego, CA, USA, catalog no. 802801) at 4°C overnight. The protein A agarose beads (30 mL, 50% slurry, GE Healthcare, IL, USA) were added to the IP reaction product to catch the immune complex at 4°C for 3 h. The immune complex on beads was washed three times with washing buffer and recombinant E1, E2, and SUMO1 (but no PIAS1) proteins were added to the IP reaction product. The remaining procedures were the same as those described above.

### Statistical Analysis

Spatial acquisition (escape latency) data were analyzed with two-way analysis of variance (ANOVA) with repeated-measure followed by a post hoc Newman-Keuls multiple comparison test (represented by q value). Retention performance data and biochemical data were analyzed with the Student’s t test or one-way ANOVA followed by Newman-Keuls comparisons. Values of p <0.05 were considered statistically significant (∗p < 0.05, ∗∗p < 0.01, ^#^p < 0.001).

## Author Contributions

Y.-C.L. designed several experiments and wrote part of the manuscript. Y.-C.L. and W.-L.H. performed most of the experiments and analyzed the data. Y.-L.M. helped with animal surgery and behavioral experiments as well as data analysis. E.H.Y.L. designed the experiments, guided and supervised the project, solved problems, and wrote most parts of the manuscript.

## Conflicts of Interest

The authors declare no competing interests.

## Supplemental Information

Document S1. Figures S1–S7 and Supplemental Materials and Methods

Document S2. Article plus Supplemental Information
