# Supplementary material for: Melatonin Induction of APP Intracellular Domain 50 SUMOylation Alleviates AD through Enhanced Transcriptional Activation and Aβ Degradation
Source: Mol Ther. 2020 Sep 5;29(1):376–95. doi: 10.1016/j.ymthe.2020.09.003 (PMC7791018; doi:10.1016/j.ymthe.2020.09.003)
Supplement: Document S1. Figures S1–S7 and Supplemental Materials and Methods [file mmc1.pdf]

YMTHE, Volume *29*

Supplemental Information

Melatonin Induction of APP Intracellular Domain 50 SUMOylation Alleviates AD through Enhanced Transcriptional Activation and Aβ Degradation

Yen-Chen Liu, Wei-Lun Hsu, Yun-Li Ma, and Eminy H.Y. Lee

Supplementary Figure 1

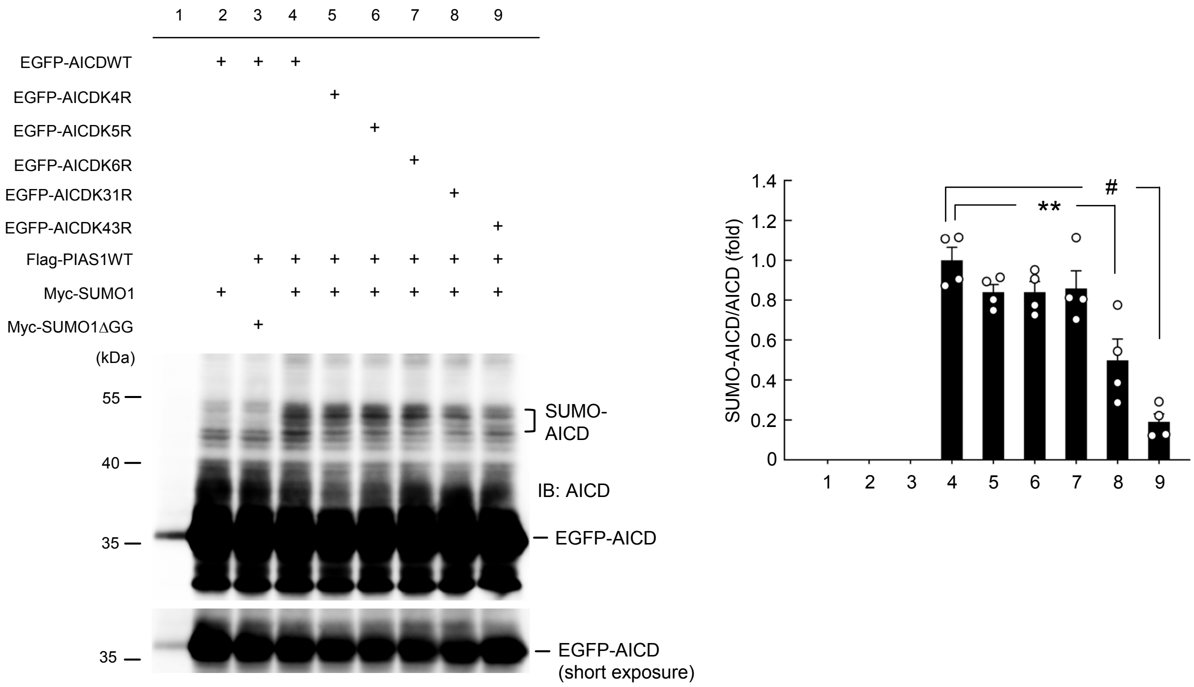

Supplementary Figure 2

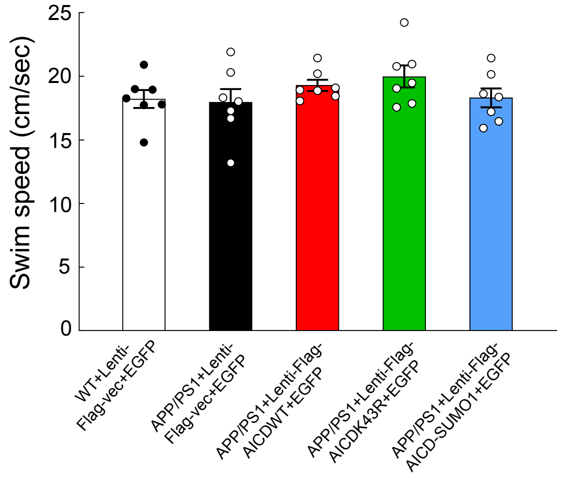

Supplementary Figure 3

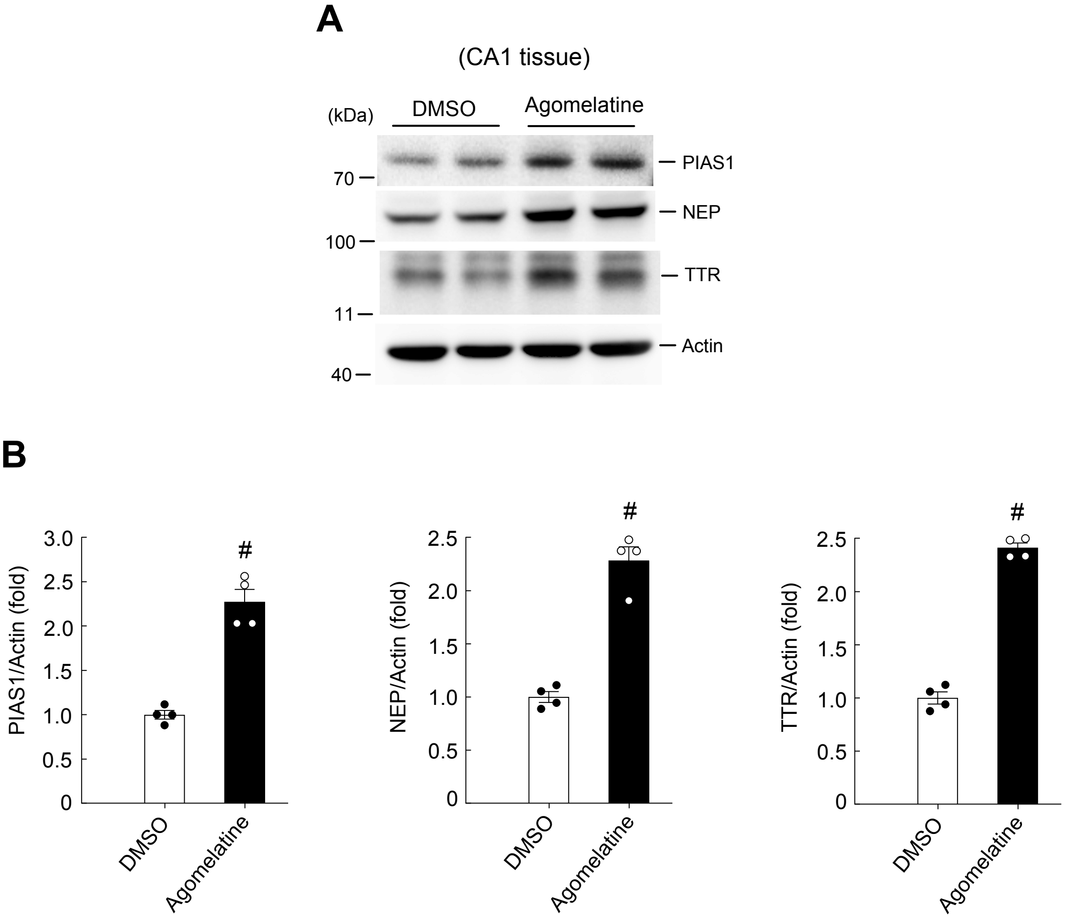

Supplementary Figure 4

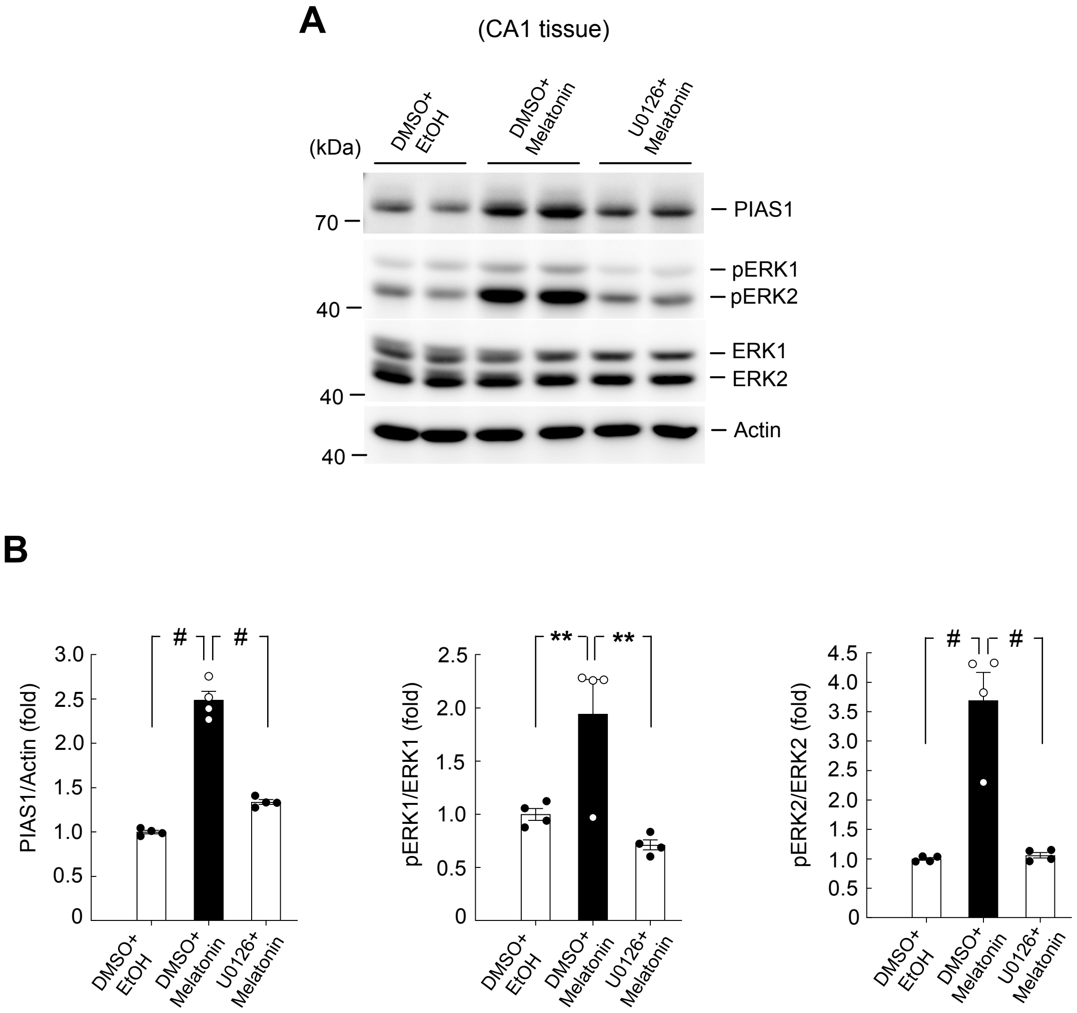

Supplementary Figure 5

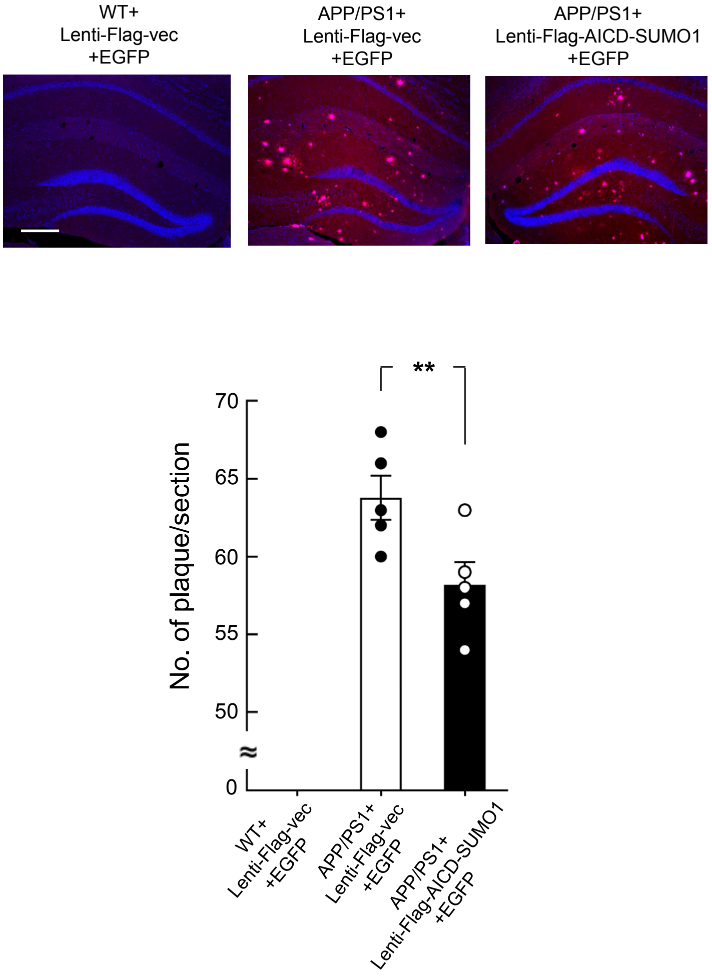

Supplementary Figure 6

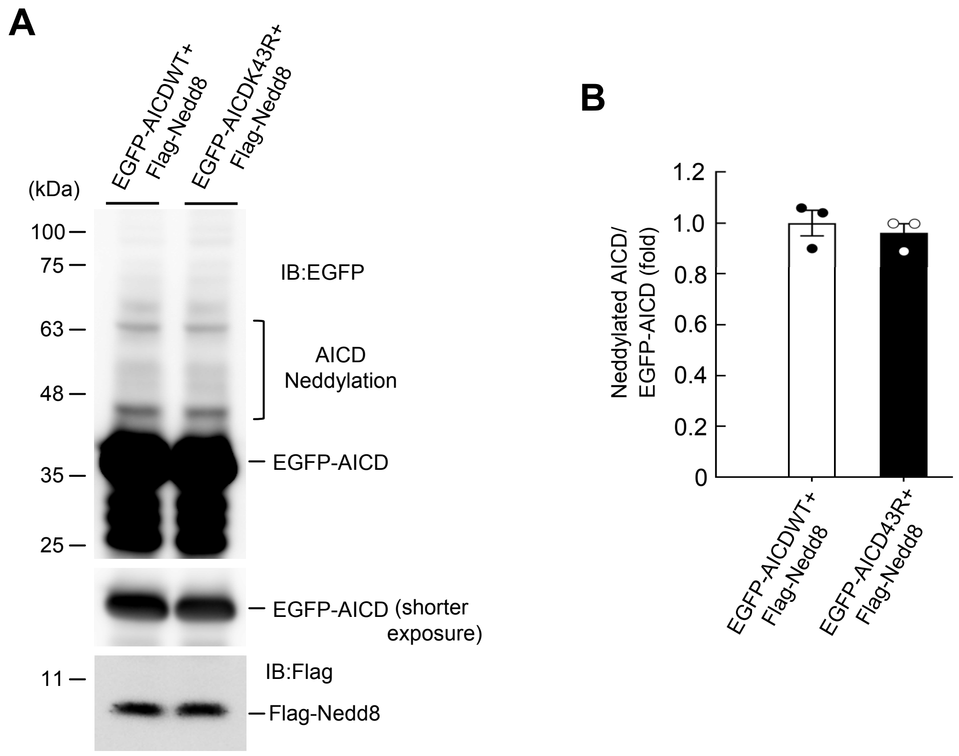

Supplementary Figure 7 All original blots

Figure 1

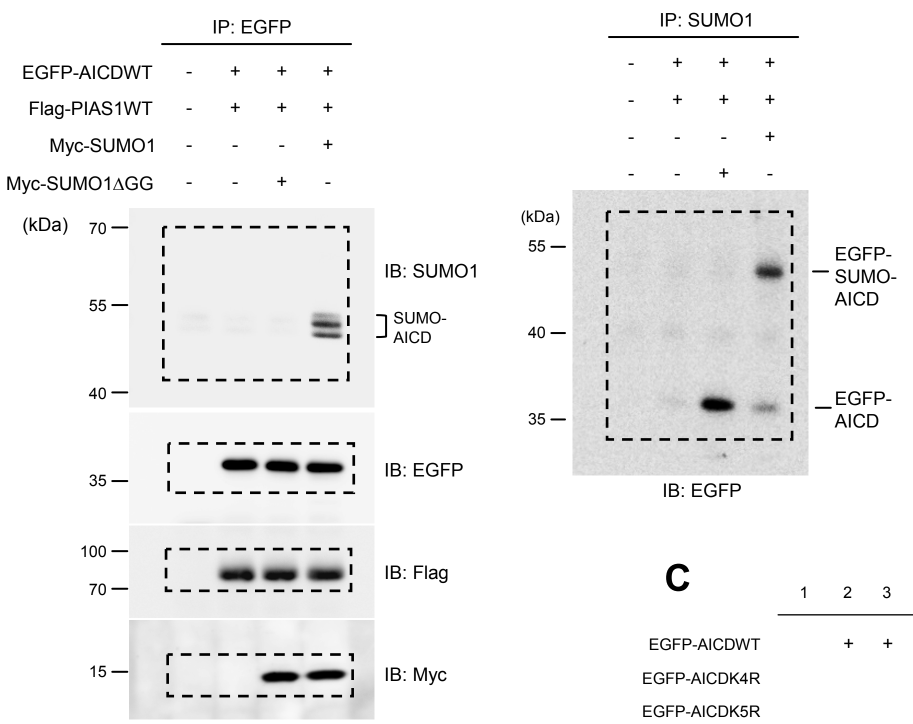

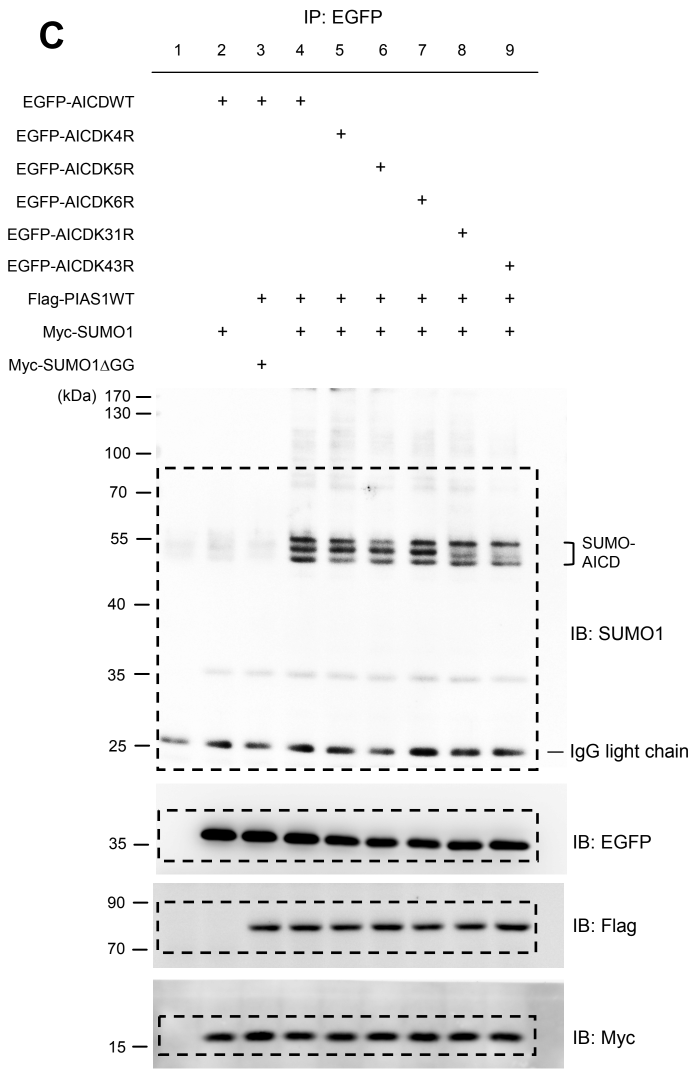

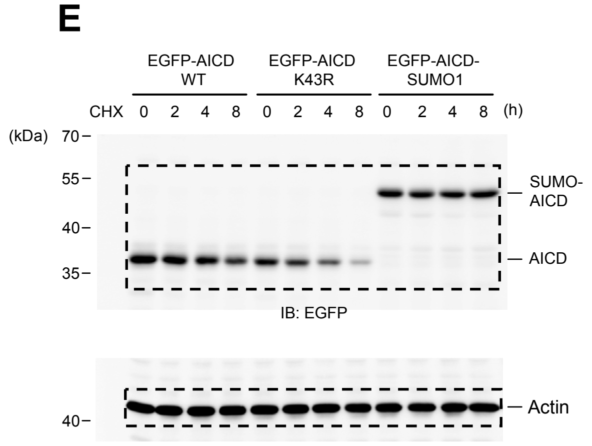

Figure 2

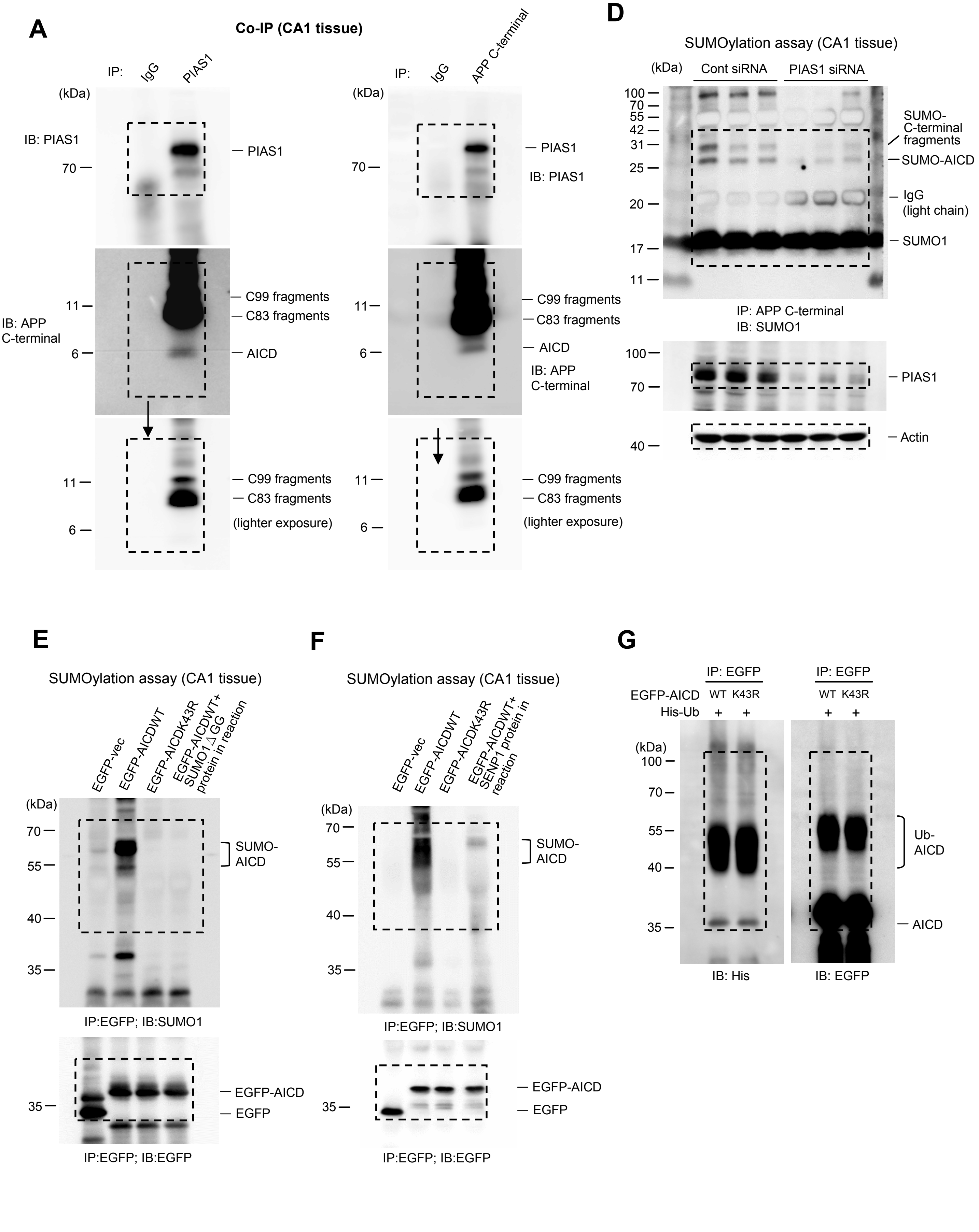
A
Co-IP (CA1 tissue)
IP: IgG PIAS1
(kDa)
IB: PIAS1
70 —
— PIAS1
IB: APP C-terminal
11 —
6 —
— C99 fragments
— C83 fragments
— AICD
11 —
6 —
— C99 fragments
— C83 fragments
(lighter exposure)
IP: IgG APP C-terminal
(kDa)
70 —
— PIAS1
IB: PIAS1
11 —
6 —
— C99 fragments
— C83 fragments
— AICD
IB: APP C-terminal
11 —
6 —
— C99 fragments
— C83 fragments
(lighter exposure)
D
SUMOylation assay (CA1 tissue)
(kDa)
Cont siRNA
PIAS1 siRNA
100 —
70 —
55 —
42 —
31 —
25 —
20 —
17 —
11 —
SUMO-C-terminal fragments
SUMO-AICD
IgG (light chain)
SUMO1
IP: APP C-terminal
IB: SUMO1
100 —
70 —
— PIAS1
40 —
— Actin
E
SUMOylation assay (CA1 tissue)
EGFP-vec
EGFP-AICDWT
EGFP-AICDK43R
EGFP-AICDWT+ SUMO1△GG protein in reaction
(kDa)
70 —
55 —
40 —
35 —
SUMO-AICD
IP:EGFP; IB:SUMO1
35 —
— EGFP-AICD
— EGFP
IP:EGFP; IB:EGFP
F
SUMOylation assay (CA1 tissue)
EGFP-vec
EGFP-AICDWT
EGFP-AICDK43R
EGFP-AICDWT+ SENP1 protein in reaction
(kDa)
70 —
55 —
40 —
35 —
SUMO-AICD
IP:EGFP; IB:SUMO1
35 —
— EGFP-AICD
— EGFP
IP:EGFP; IB:EGFP
G
IP: EGFP
IP: EGFP
EGFP-AICD WT K43R WT K43R
His-Ub + + + +
(kDa)
100 —
70 —
55 —
40 —
35 —
Ub-AICD
— AICD
IB: His
IB: EGFP

Figure 3

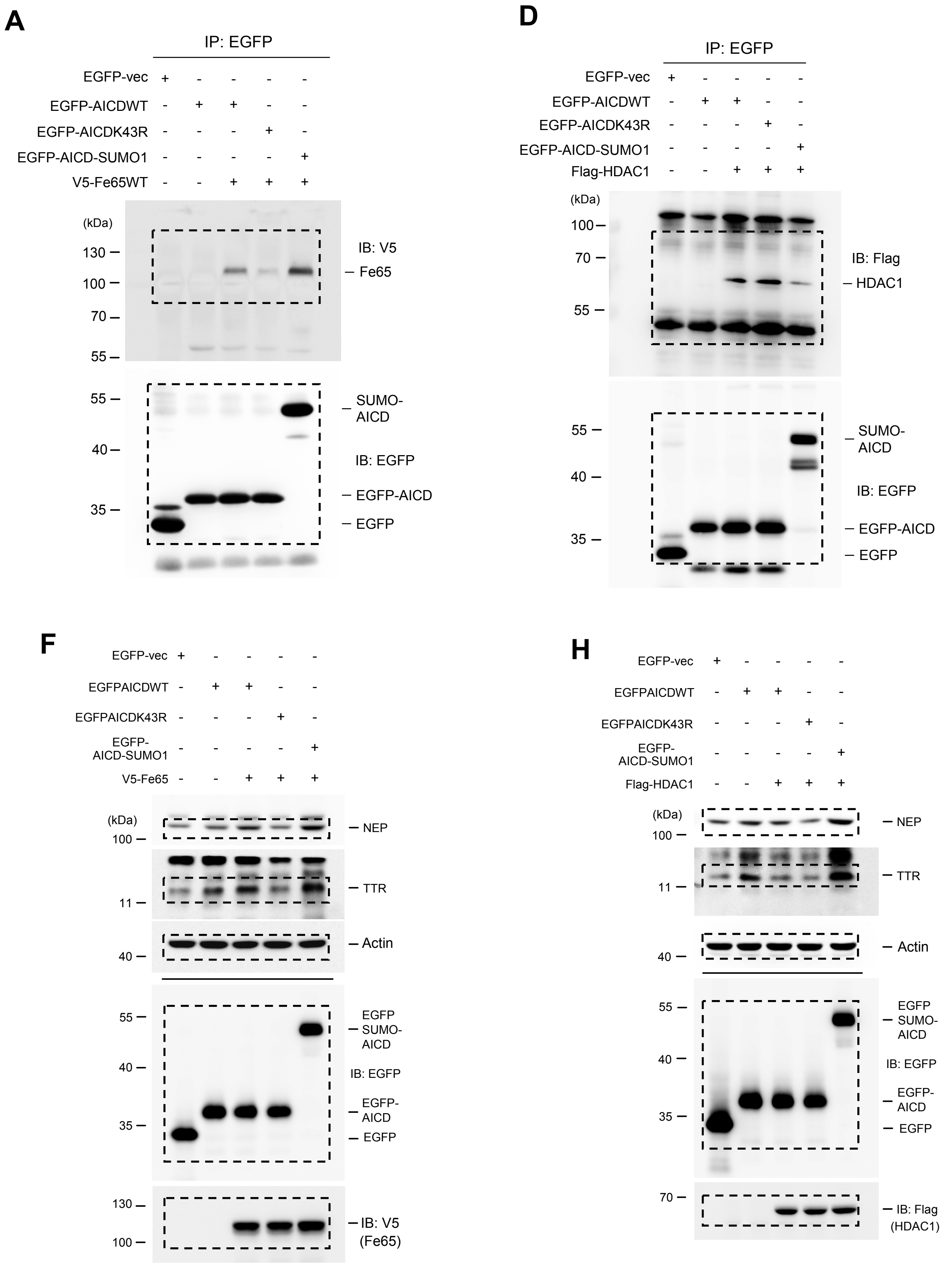

A
IP: EGFP
EGFP-vec + - - - -
EGFP-AICDWT - + + - -
EGFP-AICDK43R - - - + -
EGFP-AICD-SUMO1 - - - - +
V5-Fe65WT - - + + +
(kDa)
130
100
70
55
IB: V5
Fe65
55
40
35
SUMO-AICD
IB: EGFP
EGFP-AICD
EGFP
D
IP: EGFP
EGFP-vec + - - - -
EGFP-AICDWT - + + - -
EGFP-AICDK43R - - - + -
EGFP-AICD-SUMO1 - - - - +
Flag-HDAC1 - - + + +
(kDa)
100
70
55
IB: Flag
HDAC1
55
40
35
SUMO-AICD
IB: EGFP
EGFP-AICD
EGFP
F
EGFP-vec + - - - -
EGFPAICDWT - + + - -
EGFPAICDK43R - - - + -
EGFP-AICD-SUMO1 - - - - +
V5-Fe65 - - + + +
(kDa)
100
NEP
11
TTR
40
Actin
55
40
35
EGFP SUMO-AICD
IB: EGFP
EGFP-AICD
EGFP
130
100
IB: V5 (Fe65)
H
EGFP-vec + - - - -
EGFPAICDWT - + + - -
EGFPAICDK43R - - - + -
EGFP-AICD-SUMO1 - - - - +
Flag-HDAC1 - - + + +
(kDa)
100
NEP
11
TTR
40
Actin
55
40
35
EGFP SUMO-AICD
IB: EGFP
EGFP-AICD
EGFP
70
IB: Flag (HDAC1)

Figure 4

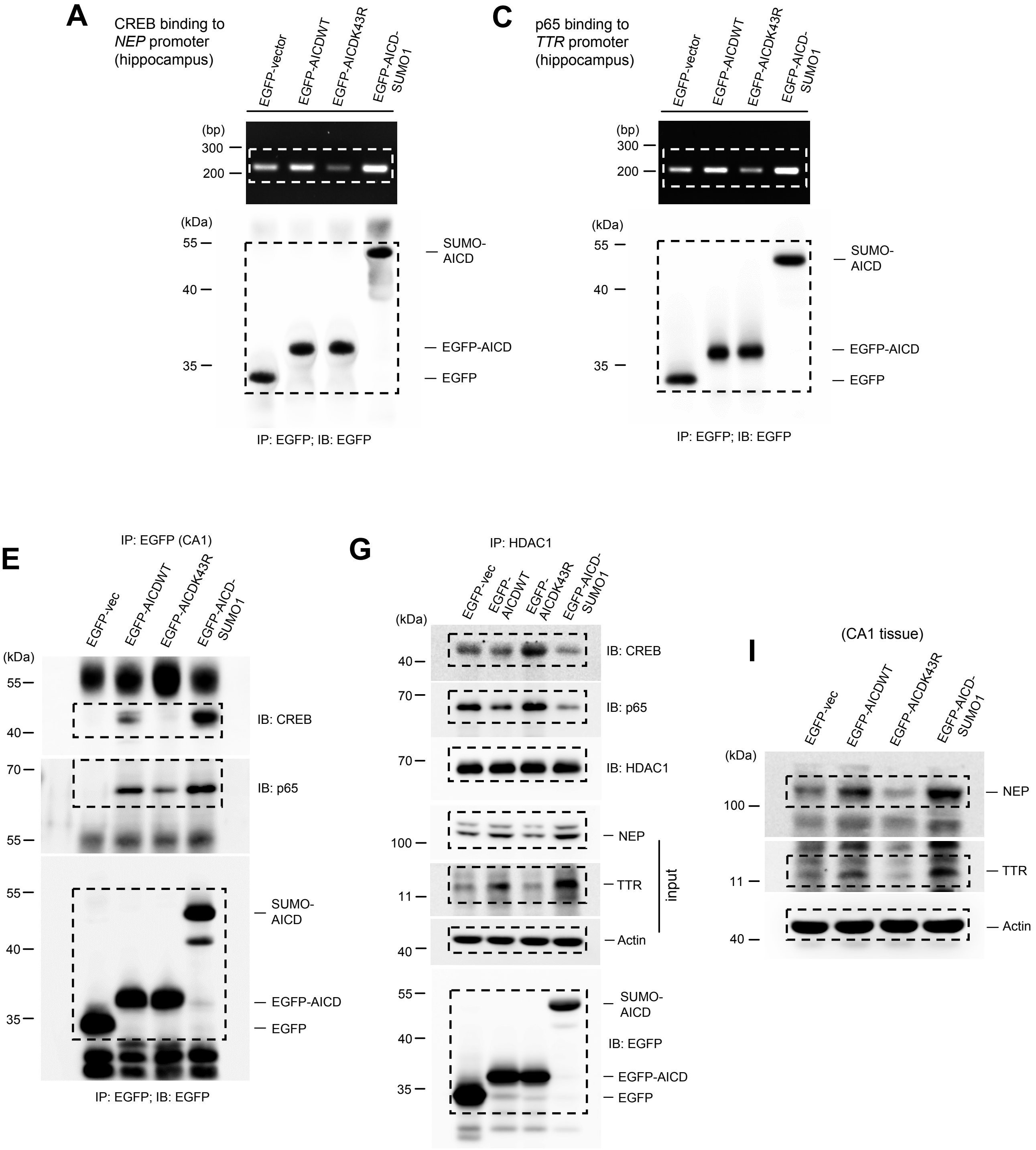

A
CREB binding to
NEP promoter
(hippocampus)
EGFP-vector
EGFP-AICDWT
EGFP-AICDK43R
EGFP-AICD-SUMO1
(bp)
300
200
(kDa)
55
40
35
SUMO-AICD
EGFP-AICD
EGFP
IP: EGFP; IB: EGFP
C
p65 binding to
TTR promoter
(hippocampus)
EGFP-vector
EGFP-AICDWT
EGFP-AICDK43R
EGFP-AICD-SUMO1
(bp)
300
200
(kDa)
55
40
35
SUMO-AICD
EGFP-AICD
EGFP
IP: EGFP; IB: EGFP
E
IP: EGFP (CA1)
EGFP-vec
EGFP-AICDWT
EGFP-AICDK43R
EGFP-AICD-SUMO1
(kDa)
55
40
70
55
IB: CREB
IB: p65
55
40
35
SUMO-AICD
EGFP-AICD
EGFP
IP: EGFP; IB: EGFP
G
IP: HDAC1
EGFP-vec
EGFP-AICDWT
EGFP-AICDK43R
EGFP-AICD-SUMO1
(kDa)
40
70
70
100
11
40
IB: CREB
IB: p65
IB: HDAC1
NEP
TTR
Actin
input
55
40
35
SUMO-AICD
IB: EGFP
EGFP-AICD
EGFP
I
(CA1 tissue)
EGFP-vec
EGFP-AICDWT
EGFP-AICDK43R
EGFP-AICD-SUMO1
(kDa)
100
11
40
NEP
TTR
Actin

Figure 5

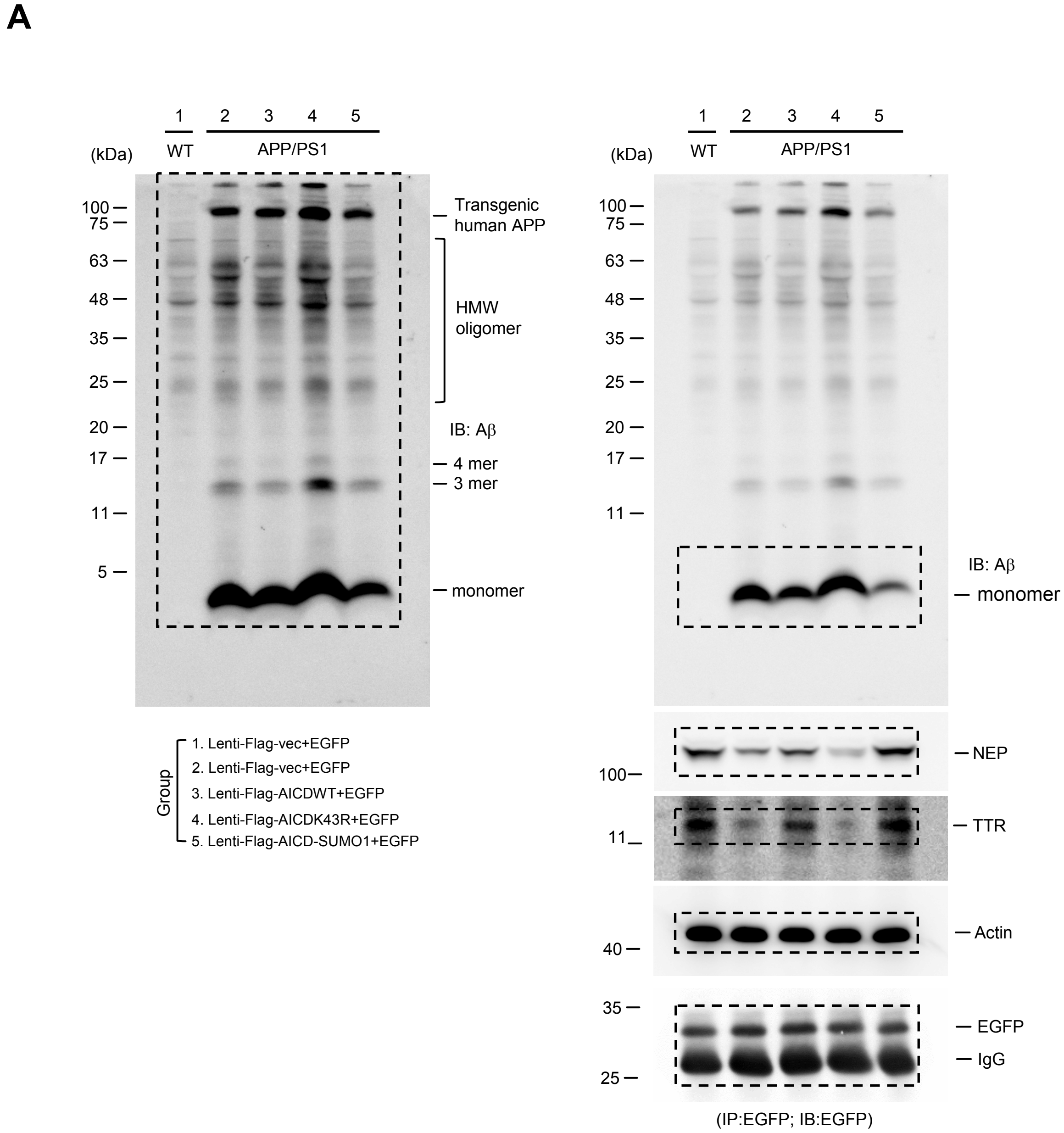
A
1 2 3 4 5
WT APP/PS1
(kDa)
100
75
63
48
35
25
20
17
11
5
Transgenic human APP
HMW oligomer
IB: Aβ
4 mer
3 mer
monomer
Group
1. Lenti-Flag-vec+EGFP
2. Lenti-Flag-vec+EGFP
3. Lenti-Flag-AICDWT+EGFP
4. Lenti-Flag-AICDK43R+EGFP
5. Lenti-Flag-AICD-SUMO1+EGFP
NEP
TTR
Actin
40
EGFP
IgG
(IP:EGFP; IB:EGFP)

Figure 6

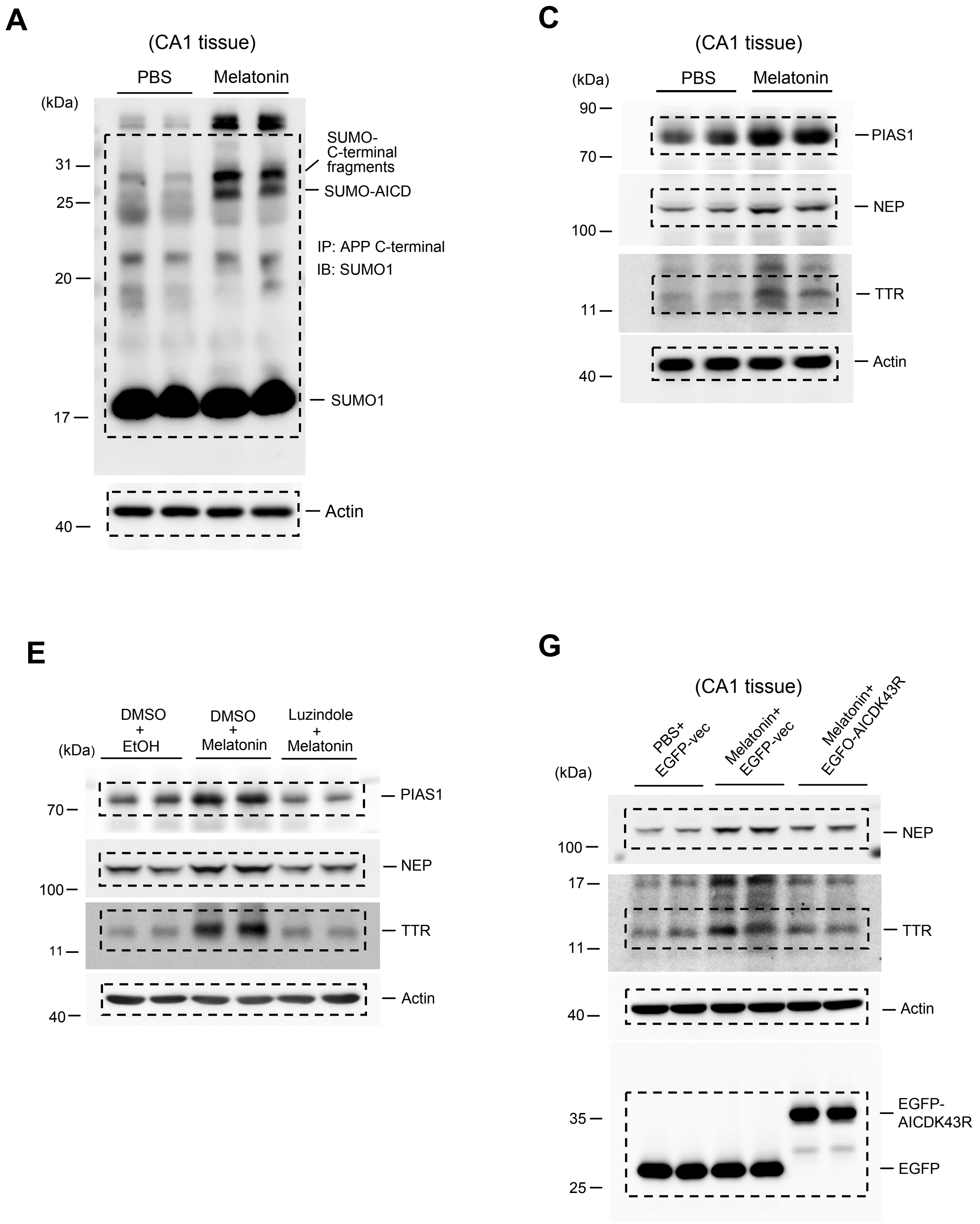

A
(CA1 tissue)
PBS
Melatonin
(kDa)
31
25
20
17
40
SUMO-
C-terminal
fragments
SUMO-AICD
IP: APP C-terminal
IB: SUMO1
SUMO1
Actin
C
(CA1 tissue)
(kDa)
PBS
Melatonin
90
70
100
11
40
PIAS1
NEP
TTR
Actin
E
DMSO
+
EtOH
DMSO
+
Melatonin
Luzindole
+
Melatonin
(kDa)
70
100
11
40
PIAS1
NEP
TTR
Actin
G
(CA1 tissue)
PBS+
EGFP-vec
Melatonin+
EGFP-vec
Melatonin+
EGFO-AICDK43R
(kDa)
100
17
11
40
35
25
NEP
TTR
Actin
EGFP-
AICDK43R
EGFP

Figure 7

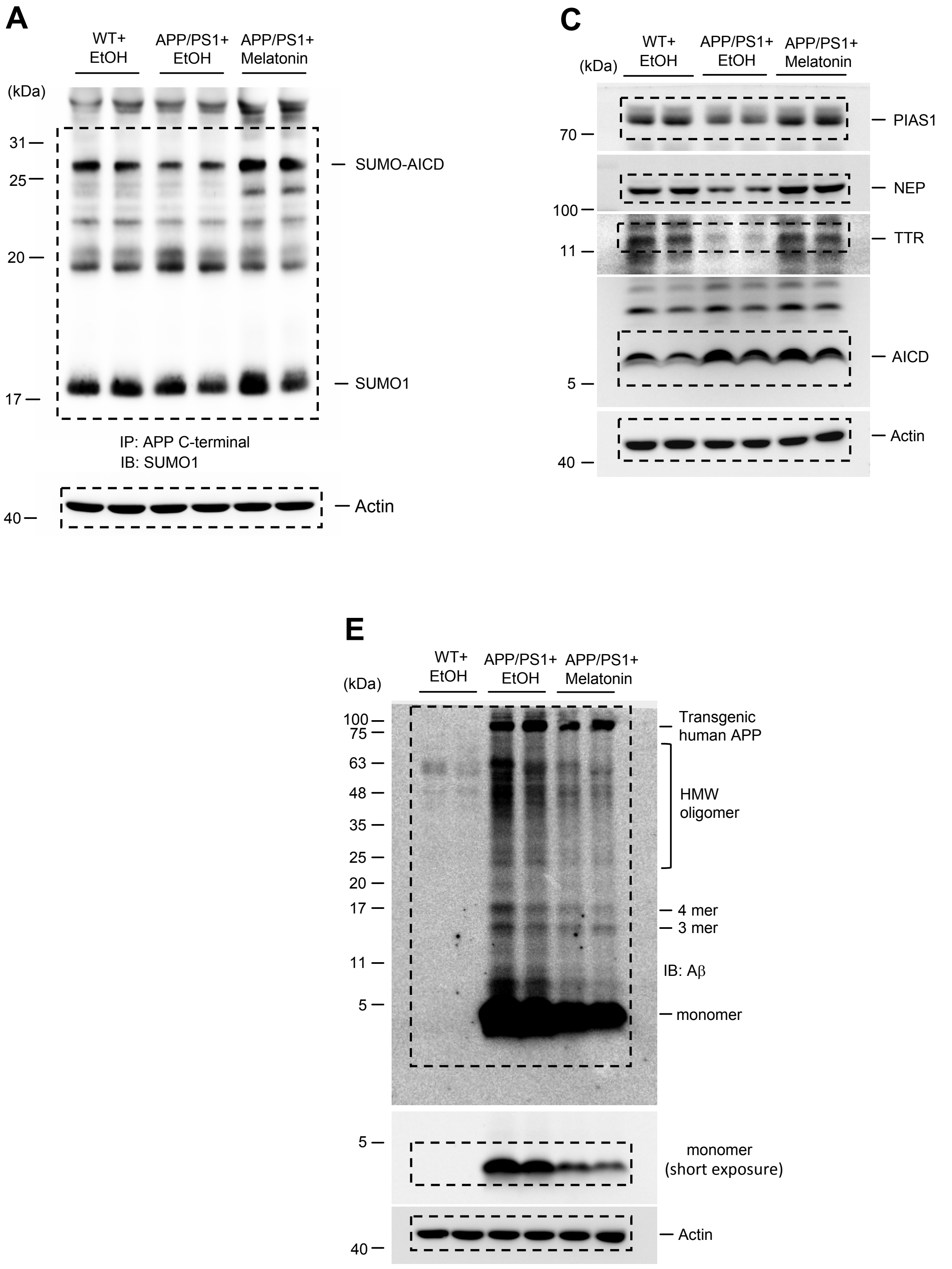
A
WT+ EtOH
APP/PS1+ EtOH
APP/PS1+ Melatonin
(kDa)
31
25
20
17
SUMO-AICD
SUMO1
IP: APP C-terminal
IB: SUMO1
40
Actin
C
WT+ EtOH
APP/PS1+ EtOH
APP/PS1+ Melatonin
(kDa)
70
100
11
5
40
PIAS1
NEP
TTR
AICD
Actin
E
WT+ EtOH
APP/PS1+ EtOH
APP/PS1+ Melatonin
(kDa)
100
75
63
48
35
25
20
17
11
5
Transgenic human APP
HMW oligomer
4 mer
3 mer
IB: Aβ
monomer
5
monomer (short exposure)
40
Actin

Supplementary Figure Legends

Figure S1 Identification of candidate SUMO sites on AICD. EGFP-tagged AICDWT plasmid or individual lysine mutant plasmids, Flag-PIAS1 plasmid, Myc-SUMO1 (or SUMO1ΔGG) plasmid were co-transfected to HEK293T cells and the cell lysate was directly immunoblotted with anti-AICD antibody. Experiments are in four repeats and results are quantified ($F_{8,27} = 55.37$, $P < 0.001$). Data are expressed as individual values and mean±SEM. ** $P < 0.01$ and $^{\#}$ $P < 0.001$.

Figure S2 Lentiviral-AICD, AICD sumo-mutant and AICD-SUMO1 fusion vector transductions do not affect swim speed of water maze learning in APP/PS1 mice. Different lenti-Flag-AICD and EGFP co-expression vector was transducted to the hippocampus of each APP/PS1 mouse (8-9 months old) and they were subjected to water maze learning two weeks later. Probe trial was conducted the next day at the end of acquisition learning. Their swim speed for the probe trial test was shown (n=7). ($F_{4,30} = 1.2$, $P > 0.05$). Data are expressed as individual values and mean±SEM.

Figure S3 Agomelatine increases PIAS1, NEP and TTR expression in rats. (A) Rats were divided to two groups (n=4) and received DMSO (37.5%) or agomelatine (3.5 μg), melatonin receptor agonist, injection to their CA1 area. Animals were

sacrificed 1 h after agomelatine injection and their CA1 tissue was subjected to western blot determination of PIAS1, NEP and TTR expression. **(B)** The quantified result of (A) ($t_{1,6}$ = 8.6 for PIAS1, $t_{1,6}$ = 9.34 for NEP and $t_{1,6}$ = 19.34 for TTR, all P < 0.001). Data are expressed as individual values and mean±SEM. $^{\#}$ P < 0.001.

Figure S4 MAPK/ERK inhibition blocks the effect of melatonin on PIAS1 expression. (A) Rats were divided to three groups (n=4) and received DMSO (40%)+EtOH (20%), DMSO+melatonin (7 μg) or U0126 (1 μg)+melatonin (7 μg) injections to their CA1 area. The two injections were separated by 30 min apart. Animals were sacrificed 1 h after melatonin (or EtOH) injection and their CA1 tissue was subjected to western blot determination of PIAS1 expression, pERK1/2 and ERK1/2 levels. **(B)** The quantified results of (A) ($F_{2,9}$ = 152.87 for PIAS1, P < 0.001; $F_{2,9}$ = 11.2 for pERK1/ERK1, P < 0.01 and $F_{2,9}$ = 30.49 for pERK2/ERK2, P < 0.001). Data are expressed as individual values and mean±SEM. ****** P < 0.01 and $^{\#}$ P < 0.001.

Figure S5 Lentiviral AICD-SUMO1 transduction decreases the amount of amyloid plaques in aged APP/PS1 mice. APP/PS1 mice of 16 months old received different lenti-Flag-AICD and EGFP co-expression vector transductions to their hippocampus. Amyloid plaque deposits (red) were examined by Proteostat dye

staining two weeks later and were quantified (n=3, 1-2 two tissue slices for each animal) ($F_{2,12}$ = 895.82, $P < 0.001$). DAPI staining is shown in blue color. Scale bar is 200 μm. Data are expressed as individual values and mean±SEM. ** $P < 0.01$.

Figure S6 Mutation of AICD at Lys-43 does not affect AICD neddylation. (A) EGFP-AICDWT or EGFP-AICDK43R plasmid was co-transfected with Flag-Nedd8 plasmid to HEK293T cells and cell lysates were immunoblotted with anti-EGFP antibody and anti-Flag antibody 48 h later. AICD neddylation from both groups is shown. The Flag-Nedd8 expression level is also shown. **(B)** Quantified result of AICD neddylation ($t_{1,4}$ = 0.62, $P > 0.05$). Results are from three independent experiments. Data are expressed as individual values and mean±SEM.

Supplementary Methods

Drugs

Melatonin was purchased from Sigma (Catalog No. M5250, St. Louis, MO). It was dissolved in 100% alcohol and further diluted with PBS to a final concentration of 10 μg/μl in 20% alcohol. The PBS solution contains 20% alcohol and was injected to animals to serve as the control group. Melatonin was prepared immediately before infusion. Agomelatine was purchased from Sigma (Catalog No. A1362) and dissolved in 100% DMSO then diluted with PBS to a final concentration of 5 μg/μl in 37.5% DMSO. Luzindole and U0126 were also purchased from Sigma (Catalog No. L2407 and Catalog No. 662005). Both drugs were dissolved in 100% DMSO and further diluted with PBS to a final concentration of 1.5 μg/μl in 40% DMSO. A volume of 0.7 μl was injected to each side of the CA1 area in rats and 0.25 μl was injected to each side of the CA1 area in mice. For intraperitoneal melatonin injection to mice, the concentration used was 10 μg/μl and 0.03 ml was injected daily.

Plasmid DNA Construction

For construction of the Flag-tagged *AICD* plasmid, full-length *AICD50* was cloned by amplifying the human *APP* cDNA (purchased from Addgene, Catalog No. 69924) with primers 5’-ATCGGGATCCGTGATGCTGAAGAAGAAAC-3’ (forward) and

5’-ATCGGATATCGTTCTGCATCTGCTCAAAG-3’ (reversed). The PCR product was sub-cloned between the *BamHI* and *EcoRV* sites of the mammalian expression vector pCMVTag2B. For construction of the Flag-tagged *PIAS1* plasmid, full-length *pias1* was cloned by amplifying the rat hippocampal *pias1* cDNA with primers 5′-ATCGGGATCCCATGGCGGACAGTGCGGAAC-3′ and 5′-ATCGGAATTCTCAGTCCAACGAGATAATG-3′. The PCR product was sub-cloned between the *BamHI* and *EcoRI* sites of the mammalian expression vector pCMV-Tag2A. For construction of the Myc-tagged *SUMO1* plasmid, full-length *sumo1* was cloned by amplifying the mouse hippocampal *sumo1* cDNA with primers 5’-GCAACCCGGGTGTCTGACCAGGAGGCAAAACCTTC-3’ (forward) and 5’-GCAAGGTACCCTAAACCGTCGAGTGACCCCCCGT-3’ (reverse). The PCR product was cloned into the mammalian expression vector pCMV-Myc. For construction of the Flag-tagged *AICD-SUMO1* fusion plasmid, the previously cloned Myc-tagged *SUMO1* plasmid was used as a template and the *sumo1* sequence was amplified with primers 5′-ATCGGTCGACATGTCTGACCAGGAGGCAA-3′ (forward) and 5′-ATCGGGGCCCCTAAACCGTCGAGTGACCC-3′ (reverse). The *SUMO1* PCR product was sub-cloned between the *SalI* and *ApaI* sites downstream of the AICD sequence from the previously cloned Flag-tagged *AICD* plasmid. A linker sequence was present on the backbone between *AICD* and *SUMO1* in order for the

AICD-SUMO1 fusion protein to be folded in the brain and the linker sequence was DIKLIDT.[23] For construction of the EGFP-tagged *AICD* plasmid and EGFP-tagged *AICD-SUMO1* fusion plasmid, the previously cloned Flag-tagged *AICD* plasmid and Flag-tagged *AICD-SUMO1*fusion plasmid were used as templates, respectively, and the *AICD* and *AICD-SUMO1* sequences were amplified with the same forward primers 5'-ATCGCGGTCCGGTGATGCTGAAGAAGAAAC-3'. The reverse primer for *AICD* was 5'-ATCGCGGACCGTTAGTTCTGCATCTGCTC-3' and that for *AICD-SUMO1* was 5'-ATCGCGGACCGCTAAACCGTCGAGTGACC-3'. The PCR product was sub-cloned into the pEGFP-C1 expression vector with *RsrII* site. Various Flag-tagged and EGFP-tagged *AICD* mutant plasmids were generated using the QuickChange Site-Directed Mutagenesis Kit (Stratagene, La Jolla, CA). For construction of the V5-tagged *Fe65* plasmid, full-length *Fe65* was cloned by amplifying the rat hippocampal *Fe65* cDNA (GeneBank: AF_333983.1) with primers 5'-ATCGGATATCATGTCTGTTCCATCATCC-3' and 5'-ATCGCCGCGGTGGGGTCTGGGATCCTAG-3'. The PCR product was sub-cloned between the *EcoRV* and *SacII* sites of the mammalian expression vector pcDNA3.1-V5-His. The *SUMO1*ΔGG mutant plasmid was generated by using site-directed mutagenesis. For construction of the His-tagged U*biquitin* plasmid, full-length *ubiquitin* was cloned by amplifying the human *ubiquitin* cDNA with

primers 5’-ATCGCCATGGATGCAGATCTTCGTGAAGAC-3’ (forward) and 5’-ATCGGGATCCTTAGACACCCCCCCTCAAGC-3’ (reverse). The PCR product was sub-cloned between the *NcoI* and *BamHI* sites of the expression vector CMV-3 x His-tag vector.

Small Interference RNA (siRNA) Transfection to the Hippocampus

The sequence for PIAS1 siRNA sense strand is 5’-UCCGGAUCAUUCUAGAGCUtt-3’ and that for PIAS1 siRNA antisense strand is 5’-AGCUCUAGAAUGAUCCGGAtt-3’. The sequence for CREB siRNA sense strand is: 5’-GCACUUAAGGACCUUUACUtt-3’ and that for CREB siRNA antisense strand is: 5’-AGUAAAGGUCCUUAAGUGCtt-3’. The Silencer Negative Control number 1 siRNA was used as the control. They were all synthesized from Ambion, Thermo Fisher Scientific (Waltham, MA).

Lentiviral Vector Construction and Preparation

For construction of Flag-AICD, Flag-AICDK43R, Flag-AICD-SUMO1 lentivitral vectors, full-length Flag-AICD, Flag-AICDK43R and Flag-AICD-SUMO fusion plasmids were sub-cloned into the lentiviral vector pLenti-Tri-cistronic (ABM, Richmond, BC, Canada) by amplifying different Flag-AICD non-viral constructs with

different primes. The forward primer is: 5'-ATCGCTCGAGGCCACCATGGATTACAAG-3'. The reverse primer for Flag-AICD and Flag-AICDK43R is 5'-ATCGCCTAGGTTAGTTCTGCATCTGCTC-3'. The reverse primer for Flag-AICD-SUMO1 is: 5'-ATCGCCTAGGCTAAACCGTCGAGTGACC-3'. These PCR products were sub-cloned between *XhoI* and *AvrII* sites of the lentiviral vector. The EGFP sequence was cloned into the pLenti-vector, pLenti-Flag-AICD, pLenti-Flag-AICDK43R and pLenti-Flag-AICD-SUMO1 vectors to obtain a cistronic co-expressing vector. The primers used for EGFP are: 5'-ATCGAGTACTGCCACCATGGTGAGCAAGGGCGAG-3' (forward) and 5'-ATCGGGTACCCTTGTACAGCTCGTCCATGCC-3' (reverse). The PCR product was sub-cloned between the *ScaI* and *KpnI* sites of the lentiviral vector.

For lentivirus packaging, HEK293LTV (Cell Biolabs, San Diego, CA) were transfected with 1.5 μg of psPAX2 (Addgene plasmid #12260), 0.5 μg of pMD2.G (Addgene plasmid #12259), and 2 μg of pLenti-EGFP, Flag-AICD-EGFP, Flag-AICDK43R-EGFP, Flag-AICD-SUMO1-EGFP plasmid using 10 μl of Lipofectamine 2000 (Invitrogen) in 6-well cell culture dish. Lentiviral particles were collected using the speedy lentivirus purification solution (ABM) according to the manufacturer's protocols. Cell culture medium containing lentiviral particles was

harvested for two to three times at 12 h interval until 36 h after transfection, and it was kept at 4 °C for the collecting period. The collected culture medium was further clarified by centrifugation at 2,500 x g for 10 min and filtrated through a 0.45 μm syringe filter. The speedy lentivirus purification solution (ABM) was added into filtrated supernatant (1:9, v/v) containing lentiviral particles and mixed thoroughly by inversion. The lentiviral supernatant was centrifuged at 5,000 x g at 4 °C for 10 min. Supernatant was then discarded and the viral pellet was re-suspended in ice cold PBS. After titration, the viral stock was stored at -80 °C in aliquots. The lentivirus titer was determined by lentivirus qPCR Titer Kit (ABM) according to the manufacturer's protocols (ABM). The final concentration of the lentiviral vector used for injection to the brain is 5 x 10^8 IU/ml.

Chromatin Immunoprecipitation (ChIP) Assay

ChIP assay was performed according to the protocol of Millipore ChIP assay kit (Catalog No. 17-10085). For plasmid DNA transfection, 0.7 μl plasmid DNA complex (1.5 μg/μl) was injected to the mouse CA1 area bilaterally 48 h before sacrifice. The hippocampal tissue containing the CA1 area were washed using ice-cold PBS and fixed with 1% formaldehyde by adding formaldehyde to the ice-cold PBS for 10 min. After adding glycine to quench the un-reacted formaldehyde, tissues were

homogenized and re-suspended in cell lysis buffer plus protease inhibitor cocktail II, then changed to nuclear lysis buffer plus protease inhibitor cocktail II for sonication. The chromatin was immunoprecipitated with 2 μl rabbit anti-CREB antibody (Cell Signaling; Catalog No. 9197) or 2 μl rabbit anti-p65 antibody (Cell Signaling; Catalog No. 8242). DNA purified from the immunoprecipitated samples was subjected to PCR reaction. The forward primer for the *neprilysin* promoter is: 5'-GACATGTATTGTCGCAGT-3' (nucleotide -2911 to -2893) and the reverse primer is: 5'-CCACAAACTTCAACTTGG-3' (nucleotide -2730 to -2713). The forward primer for the *transthyretin* promoter is: 5'- GAAATATTCTTAACTGGTC-3' (nucleotide -212 to -230) and reverse primer is: 5'-GTCAATAAACAAAAAACGA-3' (nucleotide -403 to -421). The PCR product for the *neprilysin* promoter is 199 bps in length and for the *transthyretin* promoter is 210 bps in length. The PCR products were separated by 2% agarose gel electrophoresis.

Immunoprecipitation (IP) and Western Blot

For IP of PIAS1, HDAC1, APP C-terminal fragment and EGFP, the clarified lysate (0.5 mg) was immunoprecipitated with 2 μl of anti-PIAS1 antibody (Epitomics; Catalog No. 2474-1; Burlingame, CA), 2 μl of anti-HDAC1 antibody (Cell Signaling; Catalog No. 5356S; Danvers, MA), 2 μl of anti-APP C-terminal fragment antibody

(Biolegend; Catalog No. 802801) or 2 μl of anti-GFP antibody (Sigma-Aldrich; Catalog No. 11814460001; Darmtadt, Germany) at 4 °C for overnight. Two microliters of rabbit or mouse IgG were used for the control group. The protein A or G magnetic beads (30 μl, 50% slurry, GE Healthcare, Barrington, IL) were added to the IP reaction product to catch the immune complex at 4 °C for 3 h. The immune complex on beads were washed three times with washing buffer containing 20 mM HEPES (pH 7.4), 150 mM NaCl, 1 mM EDTA, 1% IGEPAL CA-630, 1 mM DTT, 50 mM β-glycerophosphate, 50 mM NaF, 10 mg/ml PMSF, 4 μg/ml aprotinin, 4 μg/ml leupeptin and 4 μg/ml pepstatin and were subjected to 8%, 10% SDS-PAGE and 13% Tris-Tricine gel followed by transferring onto the Nitrocellulose (NC) membrane (GE Healthcare). Western blot was conducted using the following antibodies: anti-PIAS1 (1:5000; Epitomics), anti-APP C-terminal fragment antibody (1:3000; Biolegend), anti-SUMO1 (1:3000; Cell Signaling; Catalog No. 4930), anti-Flag M2 (1:8000; Sigma-Aldrich; Catalog No. F1804), anti-GFP (1:8000; Sigma-Aldrich), anti-V5 (1:8000; AbD Serotec; Catalog No. MCA2895; Kidlington, UK), anti-His (1:5000; Millipore; Catalog No. OB05, Bedford, MA), anti-CREB (1:2000; Cell Signaling; Catalog No. 9197), anti-p65 (1:3000; Cell Signaling; Catalog No. 8242), anti-neprilysin (1:500; Santa Cruz Biotechnology; Catalog No. SC-46656, Dallas, TX), anti-transthyretin (1:500; Santa Cruz Biotechnology; Catalog No. SC-377517),

anti-β-amyloid (1:1000; Biolegend; Catalog No. 803001), anti-AICD (1:2000; Biolegend; Catalog No. 811901), anti-phospho-MAPK (ERK1/2) (1:5000; Cell Signaling; Catalog No. 4376), anti-MAPK (ERK1/2) (1:5000; Cell Signaling; Catalog No. 4695) and anti-actin (1:200000; Milipore; Catalog No. MAB1501) antibodies. Aβ oligomerization was conducted and measured as that described previously. The secondary antibody used was HRP-conjugated goat-anti rabbit IgG antibody or HRP-conjugated goat-anti mouse IgG antibody (1:8000, Catalog No. 111-035-003 and 115-035-003, Jackson ImmunoResearch, West Grove, PA). The secondary antibody used for co-IP experiment was HRP-conjugated goat-anti rabbit IgG light chain (1:6000, Catalog No. NBP2-75935, Novus Biologicals, Centennial, CO) or HRP-conjugated goat-anti mouse IgG light chain antibody (1:6000, Catalog No. AP200P, Sigma-Aldrich). Membrane was developed by reacting with chemiluminescence HRP substrate (Millipore) and was exposed to the LAS-3000 image system (Fujifilm, Tokyo, Japan) for visualization of protein bands. The protein bands were quantified by using the NIH Image J Software.

Immunohistochemistry

For immunohistochemical staining of PIAS1 and APP C-terminal fragment in CA1 area of the rat brain, rats were anesthetized with pentobarbital (100 mg/kg, i.p.) and

perfused with ice-cold phosphate-buffered saline followed by 4% paraformaldehyde. Brains were removed and post fixed in 30% sucrose/4% paraformaldehyde solution for 20-48 h. Brains were then frozen, cut into 30-μm sections on a cryostat and mounted on gelatin-coated slides. Brain sections were rinsed with PBS for 10 min and antigen was retrieved with 0.1 M citric acid/0.1 M sodium citrate buffer at 95 °C for 45 min followed by PBS wash for 10 min for three times. The sections were pre-incubated in a blocking solution containing 3% BSA and 0.5% Triton X-100 in PBS for 1 h. For visualization of endogenous PIAS1 and APP C-terminal fragment in hippocampal CA1 neurons, brain sections were incubated with rabbit anti-PIAS1 antibody (Epitomics; 1:200; Catalog No. 2474-1) and mouse anti-APP C-terminal fragment antibody (Biolegend; Catalog No. 802801) at 4 °C overnight. Brain sections were then washed with PBS for 10 min for three times and incubated with goat anti-mouse antibody conjugated with FITC (1:500; Jackson ImmunoResearch Laboratories; Catalog No. 115-095-003) and goat anti-rabbit antibody conjugated with Cy3 (1:500; Jackson ImmunoResearch Laboratories; Catalog No. 111-165-003) for 1 h and then washed with PBS for 10 min for three times. For immunofluorescence detection of the nucleus, tissue sections were added with 20 μl of the DAPI Fluoromount-G mounting medium (SouthernBiotech, Birmingham, AL).

For immunofluorescence detection of amyloid plaque in the CA1 area, mice were anesthetized with pentobarbital (100 mg/kg, i.p.) followed by the same procedures as described above. For visualization of endogenous amyloid plaque in APP/PS1 mice, brain sections containing the CA1 area were incubated with ProteoStat Amyloid Plaque Detection Kit (Enzo Life Sciences; Catalog No. ENZ-51035) for 30 min and washed with PBS for 10 min for three times at room temperature. This detection kit was used because we have previously demonstrated that ProteoStat dye staining well co-localizes with anti-amyloid-beta staining in the hippocampus.[22] The brain sections were then mounted with 20 μl DAPI Fluoromount-G mounting medium (SouthernBiotech). Photomicrographs were taken using a Zeiss LSM700 confocal microscope (Carl Zeiss, Oberkochen, Germany). The number of plaques showing ProteoStat dye staining was counted by using the NIH Image J Software.

Immunofluorescence

Cultured Neuro2A cells transfected with EGFP-vector, EGFP-AICD WT, EGFP-AICD K43R, EGFP-AICD SUMO1 and V5-Fe65 plasmids on glass coverslips were washed with PBS for 5 min and fixed with 4% paraformaldehyde for 10 min at room temperature followed by PBS wash for 10 min for three times. The cells were

pre-incubated in a blocking solution containing 3% BSA and 0.1% Triton X-100 in PBS for 1 h at room temperature. Primary mouse anti-V5 antibody (1:200; AbD Serotec; Catalog No. MCA2895) was added with 3% BSA and 0.1% Triton X-100 in PBS at 4°C overnight. Cultured Neuro2A cells were then washed with PBS for 10 min for three times and incubated with goat anti-mouse antibody conjugated with Cy3 (1:500; Jackson ImmunoResearch Laboratories; Catalog No. 115-165-003) for 1 h and then washed with PBS for 10 min for three times. For immunofluorescence detection of the nucleus, Neuro2A cells on glass coverslips were added with 10 μl of the DAPI Fluoromount-G mounting medium (SouthernBiotech ; Catalog No. 0100-20, Birmingham, AL). Cultured HEK293T cells transfected with EGFP-AICDWT plasmid on glass coverslips were washed with PBS for 5 min and fixed with 4% paraformaldehyde for 10 min at room temperature followed by PBS wash for 10 min for three times. The cells were pre-incubated in a blocking solution containing 3% BSA and 0.1% Triton X-100 in PBS for 1 h at room temperature. Primary rabbit anti-PIAS1 antibody (Epitomics; 1:200; Catalog No. 2474-1) was added with 3% BSA and 0.1% Triton X-100 in PBS at 4°C overnight. HKE293T cells on glass coverslips were then washed with PBS for 10 min for three times and incubated with goat anti-rabbit antibody conjugated with Cy3 (1:500; Jackson ImmunoResearch Laboratories; Cat. No. 111-165-003) for 1 h and then washed with PBS for 10 min for

three times. The HEK293T cells on glass coverslips were then mounted with 10 μl DAPI Fluoromount-G mounting medium (SouthernBiotech). Photomicrographs were taken using a Zeiss LSM700 confocal microscope (Carl Zeiss).

Water Maze Learning

The water maze used was a plastic, circular pool, 1.2 m in diameter and 25 cm in height that was filled with water (25 ± 2 °C) to a depth of 16 cm. A circular platform of 8 cm in diameter was placed at a specific location away from the edge of the pool. The top of the platform was submerged 0.6 cm below the water surface. Water was made cloudy by adding milk powder. Distinctive, visual cues were set on the wall. For spatial learning, animals were subjected to three trials a day with one given early in the morning, one given in the early afternoon and the other one given in the late afternoon. The training procedure lased for 5 days and a total of 15 trials were given. For these trials, animals were placed at different starting positions spaced equally around the perimeter of the pool in a random order. Animals were given 60 sec to find the platform. If an animal could not find the platform within 60 sec, it was guided to the platform and was allowed to stay on the platform for 20 sec. The time that each animal took to reach the platform was recorded as the escape latency. A probe trial of 60 sec was given on day 6 to test their memory retention. Animals were placed in the

pool with the platform removed and the time they spent in each quadrant (target quadrant, left quadrant, opposite quadrant and right quadrant) as well as the total distance travelled in the target quadrant were recorded.
