# Supplementary material for: Melatonin Induction of APP Intracellular Domain 50 SUMOylation Alleviates AD through Enhanced Transcriptional Activation and Aβ Degradation
Source: Mol Ther. 2020 Sep 5;29(1):376–95. doi: 10.1016/j.ymthe.2020.09.003 (PMC7791018; doi:10.1016/j.ymthe.2020.09.003)
Supplement: Document S2. Article plus Supplemental Information [file mmc2.pdf]

Molecular Therapy
Original Article

Melatonin Induction of APP Intracellular Domain 50 SUMOylation Alleviates AD through Enhanced Transcriptional Activation and Aβ Degradation

Yen-Chen Liu,[1,2] Wei-Lun Hsu,[2] Yun-Li Ma,[2] and Eminy H.Y. Lee[1,2]

[1]Graduate Institute of Life Sciences, National Defense Medical Center, Taipei 114, Taiwan; [2]Institute of Biomedical Sciences, Academia Sinica, Taipei 115, Taiwan

The amyloid precursor protein (APP) intracellular domain (AICD) is implicated in the pathogenesis of Alzheimer's disease (AD), but post-translational modification of AICD has rarely been studied and its role in AD is unknown. In this study, we examined the role and molecular mechanism of AICD SUMOylation in the pathogenesis of AD. We found that AICD is SUMO-modified by the SUMO E3 ligase protein inhibitor of activated STAT1 (PIAS1) in the hippocampus at Lys-43 predominantly, and that knockdown of PIAS1 decreases endogenous AICD SUMOylation. AICD SUMOylation increases AICD association with its binding protein Fe65 and increases AICD nuclear translocation. Furthermore, AICD SUMOylation increases AICD association with cyclic AMP-responsive element binding protein (CREB) and p65 and their DNA binding for transcriptional activation of neprilysin (NEP) and transthyretin (TTR), two major Aβ-degrading enzymes, respectively. Consequently, AICD SUMOylation decreases the Aβ level, Aβ oligomerization, and amyloid plaque deposits. It also rescues spatial memory deficits in APP/PS1 mice. Conversely, blockade of AICD SUMOylation at Lys-43 produces the opposite effects. Melatonin is identified as an endogenous stimulus that induces AICD SUMOylation. It also decreases the Aβ level and rescues reduction of PIAS1, NEP, and TTR expression in APP/PS1 mice. In this study, we demonstrate that AICD SUMOylation functions as a novel endogenous defense mechanism to combat AD.

INTRODUCTION

The amyloid precursor protein (APP) intracellular domain (AICD) is known to regulate apoptosis, cytoskeletal dynamics, cell cycle re-entry, DNA repair, nuclear signaling, and transcriptional regulation, all of which are involved in Alzheimer's disease (AD).[1] AICD is generated through both the amyloidogenic pathway and nonamyloidogenic pathway. In the amyloidogenic pathway, which mainly occurs in the endosome, AICD is generated via sequential, proteolytic cleavages of APP by β-secretase (BACE1) and γ-secretase.[2] AICD then interacts with other proteins, including Fe65 and Tip60, and translocates to the nucleus for nuclear signaling and regulation of gene transcription.[3,4] In the nonamyloidogenic pathway, which mainly acts at the cell membrane, AICD is generated via sequential, proteolytic cleavages of APP by α-secretase and γ-secretase.[1,5] Regardless of the pathway by which AICD is generated, it is unstable and easily degraded through cleavage by γ-secretase.[6] Similar to the Notch intracellular domain, nuclear AICD is suggested to function as a transcriptional regulator.[7,8] Previous studies have shown that AICD directly binds to the promoter of neprilysin (NEP), an Aβ-degrading enzyme, and regulates NEP expression.[9,10] Transthyretin (TTR) was originally recognized as a transport protein for thyroxine and retinol. Additional studies revealed that Aβ is a protease substrate of TTR.[11] TTR was found to bind to Aβ and protect against Aβ toxicity by proteolytic cleavage of Aβ and inhibition of Aβ aggregation.[12,13] When γ-secretase activity was inhibited in cells overexpressing APP695 (in which AICD production is presumably reduced), the *TTR* mRNA level was found to be decreased,[14] suggesting that AICD regulates *TTR* mRNA expression.

APP is subject to several posttranslational modifications. APP was found to be phosphorylated at seven residues. Among these residues, the Thr-668 phosphorylation level was found to be significantly increased in the hippocampi of AD patients.[15] APP was also found to be ubiquitinated at several residues (Lys-649–Lys-651 and Lys-688), with APP ubiquitination regulating Aβ generation and APP maturation and degradation.[16,17] In addition to phosphorylation and ubiquitination, neddylation also occurs at multiple lysine residues of APP, with Nedd8 conjugation to the APP C-terminal fragment impairing the interaction of AICD with Fe65 and inhibiting AICD-mediated transcriptional activation.[18] Furthermore, APP was found to be SUMO-modified by the SUMO E2 ligase Ubc9 at Lys-587 and Lys-595, and APP SUMOylation decreased the level of Aβ aggregates in cells transfected with AD-associated mutant APP.[19]

Protein SUMOylation plays important roles in the regulation of various cellular functions.[20,21] In the context of AD, we recently showed that enhanced SUMOylation of histone deacetylase 1 (HDAC1) and Elk-1 promotes neuronal survival and protects against Aβ toxicity in APP/PS1 mice.[22,23] Although a previous report

Received 4 April 2020; accepted 1 September 2020;
https://doi.org/10.1016/j.ymthe.2020.09.003.

Correspondence: Eminy H.Y. Lee, PhD, Institute of Biomedical Sciences, Academia Sinica, Taipei 115, Taiwan.
E-mail: eminy@gate.sinica.edu.tw

 © 2020 The Author(s).
This is an open access article under the CC BY license (http://creativecommons.org/licenses/by/4.0/).

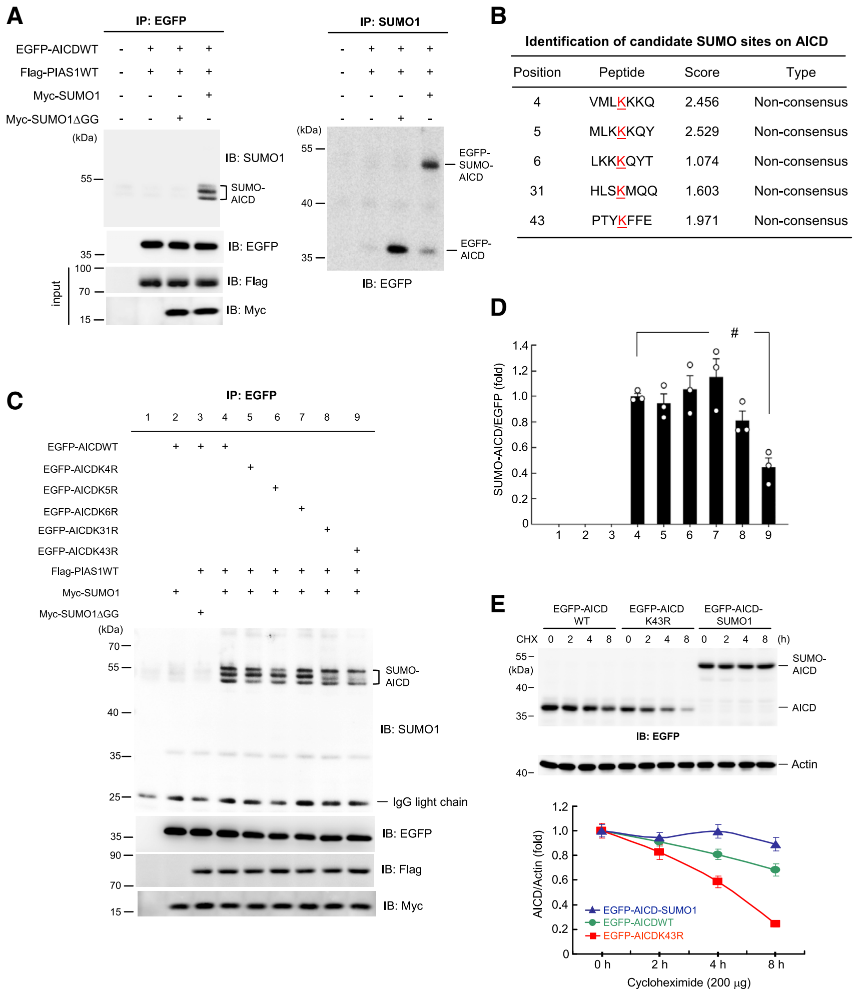

Figure 1. Identification of Candidate SUMO Sites on AICD

(A) EGFP-AICD plasmid, FLAG-PIAS1 plasmid, and Myc-SUMO1 (or SUMO1ΔGG) plasmid were co-transfected into HEK293T cells. Cell lysate was immunoprecipitated with anti-EGFP antibody and immunoblotted with anti-SUMO1 antibody. The SUMO-AICD bands were observed. Cell lysate was also immunoblotted with anti-EGFP, anti-FLAG, and anti-Myc antibodies to confirm the transfection and expression of various plasmids (left panel). Cell lysates were also immunoprecipitated with anti-SUMO1 antibody and immunoblotted with anti-EGFP antibody (right panel). (B) SUMO2.0 software prediction of candidate SUMO acceptors on AICD. The underlined letter "K" indicates the candidate SUMO sites. (C) EGFP-tagged AICDWT plasmid or individual lysine mutant plasmids, FLAG-PIAS1 plasmid, and Myc-SUMO1 (or SUMO1ΔGG) plasmid were co-transfected into HEK293T cells. Cell lysate was immunoprecipitated with anti-EGFP antibody and immunoblotted with anti-SUMO1 antibody. The SUMO-AICD bands under each condition are shown. Cell lysate was also immunoblotted with anti-EGFP, anti-FLAG, and anti-Myc antibodies to confirm the transfection and expression of various plasmids. (D) Quantified results of (C) ($F_{8,18} = 45.81$, $q = 7.61$, $p < 0.001$ comparing lane 4 and lane 9). (E) Different EGFP-tagged AICD plasmids were transfected into HEK293T cells (200 ng per well). Cycloheximide (200 μg/mL) was added to the cell 24 h after plasmid transfection for different time periods (0, 2, 4, and 8 h). Cell lysates were prepared for western blotting of AICD expression using anti-EGFP antibody. CHX, cycloheximide. The quantified results of EGFP-AICD expression for each group are also shown. Experiments are in three repeats for (A) and (C), and four repeats for (E). Data are expressed as individual values and mean ± SEM. $^{\#}p < 0.001$.

indicated SUMOylation of APP, it is not known whether the AICD protein can be SUMO-modified and what the functional significance of such AICD SUMOylation would be. In this study, we sought to examine the role and molecular mechanism of AICD SUMOylation in the pathogenesis of AD by adopting APP/PS1 mice as a mouse model of AD. Our results reveal that AICD is SUMO-modified by the protein inhibitor of activated STAT1 (PIAS1), a SUMO E3 ligase, in the hippocampus endogenously and that AICD SUMOylation decreases the level of Aβ and amyloid plaque by facilitating the clearance of Aβ. AICD SUMOylation also rescues spatial learning and memory deficits in APP/PS1 mice. In addition, melatonin is identified as an endogenous stimulus that induces AICD SUMOylation. In this study, we have identified a novel posttranslational regulation of AICD and a novel defense mechanism protecting against AD through AICD SUMOylation.

RESULTS

AICD50 Is SUMO-Modified by PIAS1 at Lys-43 in Cells

As mentioned in the Materials and Methods, in the present study we examined AICD50 protein (abbreviated as AICD hereinafter). We first examined whether AICD could be SUMO-modified by PIAS1 in cells. Different combinations of the EGFP-, FLAG- and Myc-tagged plasmids were transfected into HEK293T cells, and the cell lysate was immunoprecipitated with anti-EGFP antibody and immunoblotted with anti-SUMO1 antibody. The results revealed that AICD was SUMO-modified in cells probably at more than one residue, but that this effect was completely blocked when Myc-SUMO1ΔGG plasmid, instead of Myc-SUMO1 plasmid, was transfected to block SUMO conjugation (Figure 1A, left panel). The same cell lysates were also immunoprecipitated with anti-SUMO1 antibody and immunoblotted with anti-EGFP antibody. The results showed that the AICD-SUMO1 band was observed only when the Myc-SUMO1 plasmid was transfected (Figure 1A, right panel). Because SUMO substrate proteins often contain the ψ-K-X-E motif, where ψ stands for a hydrophobic amino acid, we next performed a bioinformatics analysis using SUMO2.0 software.[24] The results indicated that there are five lysine residues on the AICD protein, but there is no consensus SUMO-substrate motif on it (Figure 1B). Because even proteins that do not contain the ψ-K-X-E motif can still be SUMO-modified and produce biological functions (e.g., cyclic

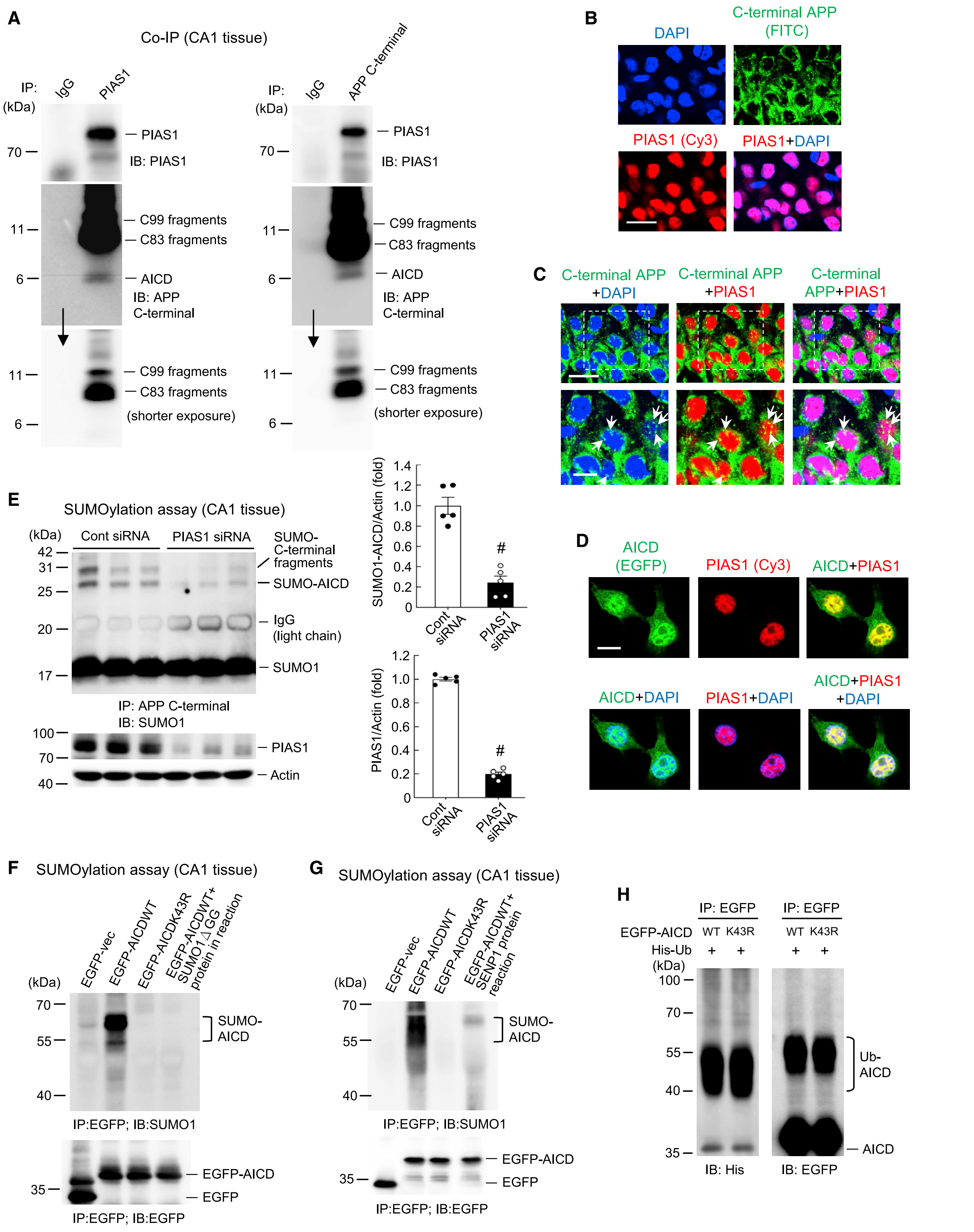

(legend on next page)

AMP-responsive element binding protein [CREB]),[25] we generated individual AICD lysine residue mutants and transfected EGFP-AICD wild-type (WT) plasmid or individual EGFP-AICD mutant plasmids together with FLAG-PIAS1WT plasmid and Myc-SUMO1 plasmid (or Myc-SUMO1ΔGG plasmid) into HEK293T cells. Co-immunoprecipitation (coIP) experiments were carried out as described above. The results confirmed that AICD could be SUMO-modified by PIAS1. Multiple SUMO-AICD bands were observed but AICD was predominantly SUMOylated at Lys-43 (Figure 1C). The quantified AICD SUMOylation level at each mutation site is shown in Figure 1D. Similar results were obtained when the cell lysate was directly immunoblotted with anti-AICD antibody (Figure S1). Because Lys-43 is the major SUMO residue on AICD, we focused on this residue in the present study. To understand the cellular effect of AICD SUMOylation, we transfected EGFP-AICDWT, EGFP-AICDK43R, and EGFP-AICD-SUMO1 fusion plasmids into HEK293T cells with cycloheximide added to the cells for different time periods. Western blotting was carried out using anti-EGFP antibody. The results revealed that AICD SUMOylation stabilizes AICD whereas the SUMO mutant AICD degrades more rapidly (Figure 1E).

AICD Is SUMO-Modified by PIAS1 Endogenously and Co-localizes with PIAS1 in the Hippocampus

After showing that AICD is SUMO-modified by PIAS1 in cells, we next examined whether AICD could be SUMO-modified by PIAS1 endogenously in the brain. We first examined the relationship between AICD and PIAS1 in the hippocampus by carrying out coIP experiments. Because commercially available AICD antibodies also recognize full-length APP, we used the APP C-terminal antibody for this experiment. Rat hippocampal CA1 tissue was immunoprecipitated with anti-PIAS1 antibody and immunoblotted with anti-APP C-terminal antibody (as well as anti-PIAS1 antibody) or vice versa. The results revealed that PIAS1 is associated with the C99 fragment, C83 fragment, and AICD in the hippocampus endogenously (Figure 2A). Next, we examined whether PIAS1 and AICD are present in the same neurons in the rat hippocampus. Brain sections containing the CA1 region were subjected to immunohistochemical staining. Antibody against PIAS1, Cy3-conjugated secondary antibody, antibody against the APP C-terminal, and FITC-conjugated secondary antibody were used. DAPI was added to the reaction for nuclear staining. The results revealed that PIAS1 (red) was present only in the nucleus. APP C-terminal staining was mainly observed in the cytosol and the surrounding area of the nucleus (green) (Figure 2B). Further visualization at a higher magnification indicated that APP C-terminal staining was also present in the nucleus and that the APP C-terminal co-localized with PIAS1 in the same neurons (yellow; indicated by arrows) (Figure 2C, lower panel). Although the APP C-terminal antibody would be expected to also stain the C83 fragment and C99 fragment in addition to AICD, only AICD nuclear translocation was observed.[3] Thus, the staining we observed in the nucleus is presumably the AICD protein. To further examine the co-localization of AICD with PIAS1, HEK293T cells were transfected with the EGFP-AICD plasmid. PIAS1 immunofluorescence was visualized using Cy3-conjugated secondary antibody against the PIAS1 antibody. The results revealed that overexpressed AICD co-localized with endogenous PIAS1 only in the nucleus (Figure 2D).

The above results showed that PIAS1 is associated with AICD and co-localizes with AICD in the same hippocampal neurons. Next, we examined whether AICD is SUMO-modified by PIAS1 in the hippocampus endogenously. Control small interfering RNA (siRNA) or PIAS1 siRNA (10 pmol) was transfected into the rat CA1 area and AICD SUMOylation was determined 48 h later. The results revealed that PIAS1 siRNA transfection significantly decreased the levels of both endogenous AICD SUMOylation (lower band) and C-terminal fragment SUMOylation (upper band) (Figure 2E). PIAS1 siRNA transfection also markedly decreased the level of PIAS1 expression (Figure 2E, lower panel). The quantified results of AICD SUMOylation and PIAS1 expression are shown in Figure 2E (right panel).

To further explore AICD SUMOylation by PIAS1 in the hippocampus, we transfected EGFP-AICDWT plasmid and EGFP-AICDK43R plasmid into the rat CA1 area and carried out an *in vitro* SUMOylation

Figure 2. AICD Is SUMO-Modified by PIAS1 Endogenously and Is Co-localized with PIAS1 in Hippocampal Neurons

(A) Rat CA1 tissue lysate was immunoprecipitated with anti-PIAS1 antibody and immunoblotted with anti-APP C-terminal antibody and anti-PIAS1 antibody (left) and vice versa (right). Experiments are in two repeats. (B) Immunohistochemistry showing the distribution of PIAS1 and C-terminal APP in hippocampal neurons (n = 3). Scale bar represents 20 μm. (C) Immunohistochemistry at a higher magnification showing co-localization of PIAS1 and C-terminal APP in the nucleus of hippocampal neurons (indicated by arrows). Scale bars represent 20 μm for the upper panel and 10 μm for the lower panel. Magnification of the dotted square area is shown in the lower panel. (D) EGFP-AICD plasmid was transfected into HEK293T cells. Cy3-conjugated secondary antibody against the PIAS1 antibody was used for visualization of PIAS1. Immunofluorescence staining showing the co-localization of AICD (green) and PIAS1 (red) in the nucleus only (n = 3). Scale bar represents 10 μm. (E) PIAS1 siRNA (10 pmol) or control siRNA was transfected into the rat CA1 area, and endogenous AICD SUMOylation as well as C-terminal fragment SUMOylation were determined 48 h later by a SUMOylation assay. PIAS1 expression was determined by western blot (n = 5). The quantified results of AICD SUMOylation and PIAS1 expression are shown in the right panel ($t_{1,8} = 7.35$ for AICD SUMOylation and $t_{1,8} = 32.91$ for PIAS1, both $p < 0.001$). Data are expressed as individual values and mean ± SEM. (F) EGFP-AICDWT (with or without the SUMO1ΔGG mutant protein added to the reaction) or EGFP-AICDK43R plasmid was transfected into the rat CA1 area, and a SUMOylation assay was carried out 48 h later. The SUMO-AICD bands in the EGFP-AICDWT group are shown. coIP with anti-EGFP antibody was conducted to confirm the expression of the plasmids. (G) The same plasmids were transfected into another batch of rats except that recombinant SENP1, instead of mutant SUMO1, protein was added to the reaction for the last group. A SUMOylation assay was carried out 48 h later. The SUMO-AICD bands in the EGFP-AICDWT group are shown. coIP with anti-EGFP antibody was conducted to confirm the expression of the plasmids. (H) EGFP-AICDWT or EGFP-AICDK43R plasmid was co-transfected with histidine (His)-ubiquitin plasmid to HEK293T cells. Cell lysate was immunoprecipitated with anti-EGFP antibody and immunoblotted with anti-His and anti-EGFP antibody. Ubiquitinated AICD is shown. Ub, ubiquitin. Experiments are in two repeats for (G) and three repeats for (F) and (H). $^{\#}p < 0.001$.

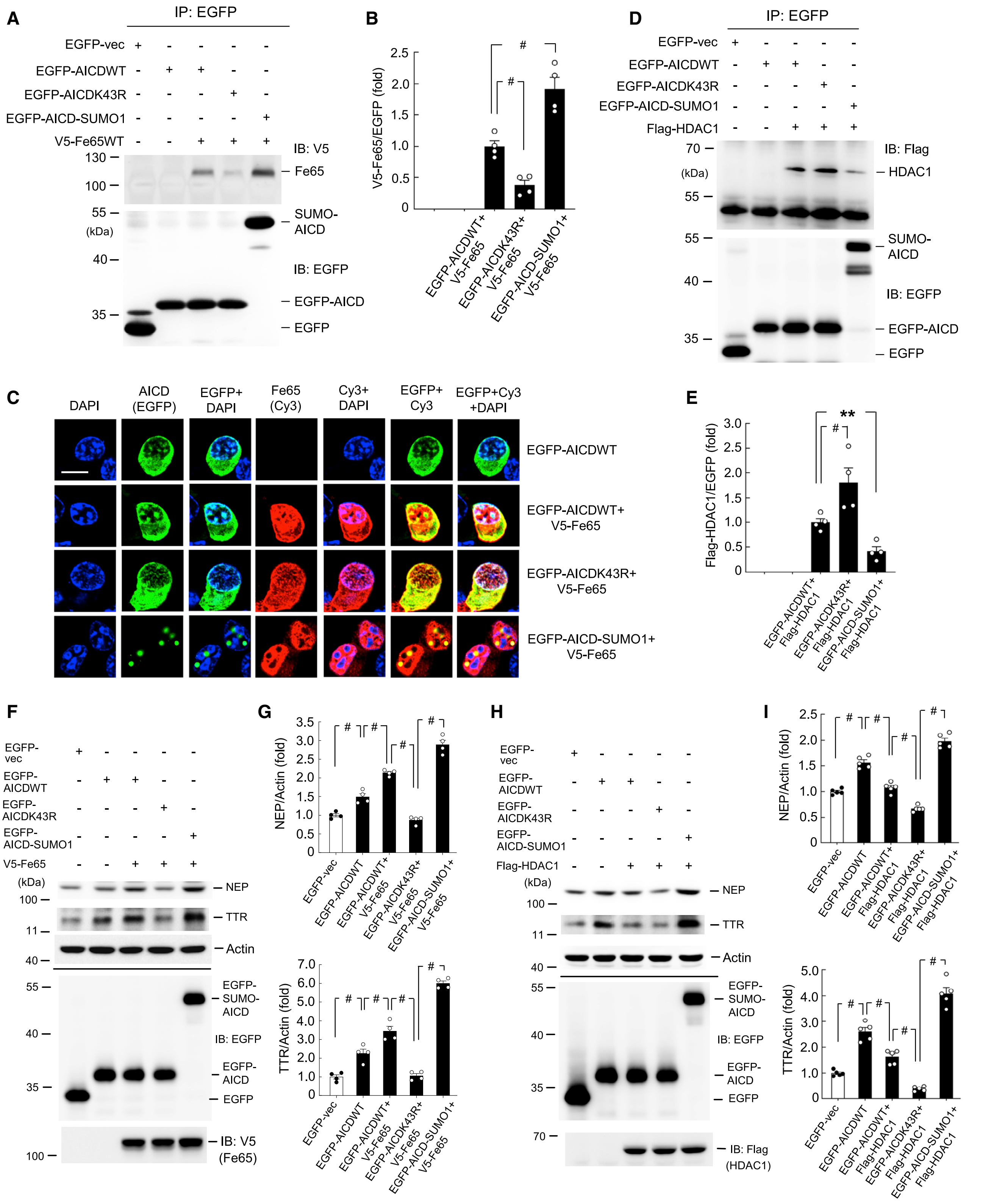

(legend on next page)

assay. This approach was taken because mutation of AICD at Lys-43 caused the greatest decrease in AICD SUMOylation in cells (Figures 1C and 1D). Cell lysate was immunoprecipitated with anti-EGFP antibody and immunoblotted with anti-SUMO1 antibody. The results indicated that AICD was apparently SUMOylated by PIAS1, but that AICD SUMOylation was completely blocked when EGFP-AICDK43R was transfected. To confirm the specificity of the AICD SUMOylation reaction, in another group we added the SUMO1ΔGG mutant protein to the reaction because this mutant prevents the SUMO molecule from forming a covalent bond with the lysine residue on AICD. The results revealed that AICD SUMOylation was abolished when EGFP-AICDWT plasmid was transfected and the SUMO1ΔGG mutant protein was added to the SUMOylation reaction mixture at the same time (Figure 2F). To further examine AICD SUMOylation by PIAS1, another group of rats received the same plasmid transfections as described above except that, instead of the SUMO1ΔGG protein, the recombinant SENP1 protein was added to the reaction. This group was included because the SENP1 enzyme removes the SUMO molecule from the lysine residue of a SUMOylated protein. The results revealed that addition of SENP1 similarly abolished AICD SUMOylation (Figure 2G).

Because ubiquitination of APP was found to take place at Lys-649–Lys-651 and Lys-688,[16,17] we next examined whether Lys-43 of AICD can be ubiquitinated and, if so, whether this ubiquitination affects AICD SUMOylation. EGFP-AICDWT or EGFP-AICDK43R plasmid was co-transfected with His-ubiquitin plasmid into HEK293T cells and cell lysate was immunoprecipitated with anti-EGFP antibody and immunoblotted with anti-His antibody and anti-EGFP antibody. The results revealed that AICD ubiquitination was not affected by EGFP-AICDK43R transfection regardless of whether it was immunoblotted with anti-His antibody or anti-EGFP antibody (Figure 2H).

AICD SUMOylation Increases Its Association with Fe65 and Decreases Its Association with HDAC1

As mentioned above, AICD is stabilized by Fe65 and translocates to the nucleus, and it forms a complex with Fe65 and Tip60 for transcriptional activation.[3,4] However, the relationship between AICD and Fe65 upon AICD SUMOylation is not known. To address this issue, different EGFP-AICD plasmids and V5-Fe65 plasmid were transfected into HEK293T cells. The cell lysates were immunoprecipitated with anti-EGFP antibody and immunoblotted with anti-V5 antibody. The results revealed that AICD is associated with Fe65, but that this association is diminished by EGFP-AICDK43R mutant transfection and is enhanced by EGFP-AICD-SUMO1 fusion plasmid transfection (Figures 3A and 3B). Cell lysates were also immunoprecipitated and immunoblotted with anti-EGFP antibody to confirm the transfection and expression of various EGFP-AICD plasmids (Figure 3A, lower panel).

To further examine the relationship between AICD SUMOylation and Fe65, different EGFP-AICD plasmids and V5-Fe65 plasmid (conjugated with Cy3 secondary antibody) were co-transfected into Neuro2A cells and immunofluorescence staining against EGFP and Cy3 was visualized. DAPI was added to the reaction for nuclear staining. As shown in Figure 3C, when EGFP-AICDWT plasmid was transfected, AICD was observed in the nucleus (EGFP), and it was co-localized with DAPI staining (EGFP+DAPI). When EGFP-AICDWT plasmid and V5-Fe65 plasmid were co-transfected, Fe65 was also present in the nucleus (Cy3), and it was mostly co-localized with DAPI staining (Cy3+DAPI). The merged image further indicated that Fe65 was mostly co-localized with AICD in the nucleus with DAPI staining (EGFP+Cy3+DAPI). In addition, Fe65 and AICD were also co-localized in part of the cytosol area (EGFP+Cy3). Alternatively, co-transfection of EGFP-AICDK43R and V5-Fe65 plasmids prevented nuclear translocation of AICD (EGFP). The presence of Fe65 in the nucleus (Cy3) was also reduced compared with the EGFP-AICDWT+V5-Fe65 transfection. Co-localization of AICD and Fe65 with DAPI (EGFP+Cy3+DAPI) was similarly diminished in the nucleus; instead, co-localization of AICD and Fe65 was observed in the cytosol area. This distribution pattern changed dramatically when the SUMO form of AICD plasmid (EGFP-AICD-SUMO1) was co-transfected with V5-Fe65 plasmid. Both AICD and Fe65 were consistently present in the nucleus, but the morphology of AICD staining was changed (EGFP), with AICD not co-localized

Figure 3. AICD SUMOylation Increases Its Association with Fe65 and Decreases Its Association with HDAC1

(A) Different EGFP-tagged AICD plasmids and V5-Fe65 plasmid were co-transfected into HEK293T cells. Cell lysate was immunoprecipitated with anti-EGFP antibody and immunoblotted with anti-V5 antibody. CoIP with anti-EGFP antibody was conducted to confirm the expression of the AICD plasmids. (B) Quantified results of (A) ($F_{4,15}$ = 66.54, $p < 0.001$; q = 6.26, $p < 0.001$ comparing the AICDK43R+Fe65 group with the AICDWT+Fe65 group; and q = 9.2, $p < 0.001$ comparing the AICD-SUMO1+Fe65 group with the AICDWT+Fe65 group). (C) Different EGFP-tagged AICD plasmids and V5-Fe65 plasmid were co-transfected into Neuro2A cells and the distributions of AICD and Fe65 and their co-localization with DAPI were examined by immunofluorescence staining using FITC- and Cy3-conjugated secondary antibodies, respectively. Scale bar represents 10 μm. Results are from three different batches of cells. (D) Different EGFP-tagged AICD plasmids and FLAG-HDAC1 plasmid were co-transfected into HEK293T cells. Cell lysate was immunoprecipitated with anti-EGFP antibody and immunoblotted with anti-FLAG antibody. coIP with anti-EGFP antibody was conducted to confirm the expression of various plasmids. (E) Quantified results of (D) ($F_{4,15}$ = 28.88, $p < 0.001$; q = 5.62, p = 0.001 comparing the AICDK43R+HDAC1 group with the AICDWT+HDAC1 group, and q = 4.09, p = 0.01 comparing the AICD-SUMO1+HDAC1 group with the AICDWT+HDAC1 group). (F) Different EGFP-tagged AICD plasmids and V5-Fe65 plasmid were co-transfected into Neuro2A cells and the expression level of NEP and TTR was determined by western blot. Immunoprecipitation and immunoblotting with anti-EGFP antibody was used to confirm the expression of various EGFP-tagged plasmid transfections. Immunoblotting with anti-V5 antibody was used to confirm the expression of V5-Fe65 transfection. (G) Quantified results of (F) ($F_{4,15}$ = 128.56 for NEP and $F_{4,15}$ = 135.15 for TTR, both $p < 0.001$). (H) The same plasmids were transfected into Neuro2A cells as described in (F) except that the V5-Fe65 plasmid was replaced with the FLAG-HDAC1 plasmid and immunoblotting with the anti-V5 antibody was replaced with the anti-FLAG antibody to confirm the expression of FLAG-HDAC1 transfection. (I) Quantified results of (H) ($F_{4,20}$ = 141.48 for NEP and $F_{4,20}$ = 199.41 for TTR, both $p < 0.001$). Experiments are in four repeats for (A), (D), and (F), and five repeats for (H). Data are expressed as individual values and mean ± SEM $^{**}p < 0.01$, $^{\#}p < 0.001$.

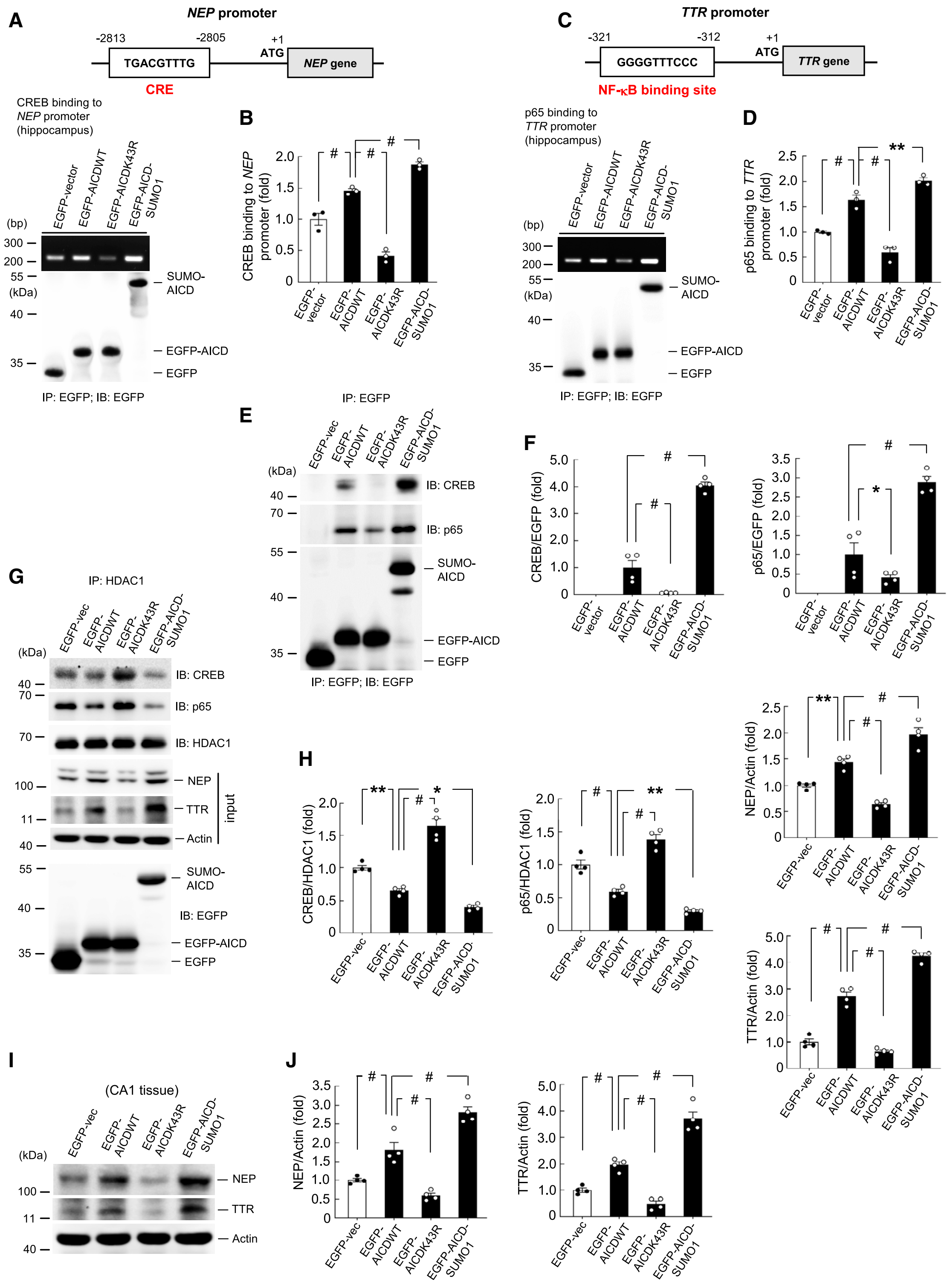

Figure 4. AICD SUMOylation Increases AICD Association with CREB and p65 and Increases CREB Binding to the *NEP* Promoter and p65 Binding to the *TTR* Promoter

(A) The position of the CRE element on the *NEP* gene promoter is shown. Different EGFP-AICD plasmids were transfected into the rat CA1 area, and a ChIP assay for CREB binding to the *NEP* promoter in the hippocampus is shown. coIP using anti-EGFP antibody was conducted to confirm the expression of various AICD plasmids. (B) Quantified

(legend continued on next page)

with DAPI staining (EGFP+DAPI). Furthermore, AICD showed good co-localization with Fe65, and together they formed nuclear puncta (EGFP+Cy3), but they did not co-localize with DAPI staining (EGFP+Cy3+DAPI). Instead, these nuclear puncta were located in the perichromatin region close to the area stained with DAPI. In addition, Fe65 staining was exclusively observed in the nucleus when the SUMO form of AICD was transfected into the cell. This is probably because when AICD is SUMOylated, Fe65 preferentially binds AICD in the nucleus and is unable to translocate to the cytosol area. This result is congruent with the previous finding that AICD forms a complex with Fe65 and Tip60 in the nucleus for transcriptional activation,[3,4] and our finding that SUMOylated AICD shows a stronger interaction with Fe65 than does WT AICD (Figure 3A).

A previous study showed that binding of HDAC1 to the *NEP* promoter and *TTR* promoter was decreased in neuroblastoma cells expressing the APP695 isoform.[14] This is probably because more AICD is generated by β- and γ-secretase cleavage of the APP695 protein and because AICD competes with HDAC1 for binding to the *NEP* and *TTR* promoters.[14] In this experiment, we examined whether AICD is associated with HDAC1 and whether this association is altered by AICD SUMOylation. Different EGFP-tagged AICD plasmids and FLAG-HDAC1 plasmid were transfected into HEK293T cells. The cell lysates were immunoprecipitated with anti-EGFP antibody and immunoblotted with anti-FLAG antibody. The results revealed that AICD interacts with HDAC1. This interaction is increased by EGFP-AICDK43R mutant transfection and is diminished by EGFP-AICD-SUMO1 fusion plasmid transfection (Figures 3D and 3E). Cell lysates were also immunoprecipitated and immunoblotted with anti-EGFP antibody to confirm the transfection and expression of various EGFP-tagged plasmids (Figure 3D, lower panel).

After showing the association between AICD and Fe65, we next examined how the interaction between AICD and Fe65 affects AICD-mediated transcriptional regulation of NEP and TTR expression in the context of AICD SUMOylation. Different EGFP-tagged AICD plasmids and V5-Fe65 plasmid were co-transfected into Neuro2A cells, and the cell lysates were subjected to western blot analysis of NEP and TTR expression. The results indicated that overexpression of AICD increased the expression levels of both NEP and TTR compared with the control group, with this effect enhanced by Fe65 co-expression. However, co-transfection of EGFP-AICDK43R and V5-Fe65 reversed the effect of AICD on NEP and TTR expression, but NEP and TTR expression levels were rescued and further increased by co-transfection of EGFP-AICD-SUMO1 fusion plasmid and V5-Fe65 plasmid (Figures 3F and 3G). These results indicated that AICD and Fe65 interaction increased NEP and TTR expression and that this effect was further enhanced by AICD SUMOylation. Next, we similarly examined whether the interaction of AICD and HDAC1 affects AICD-mediated regulation of NEP and TTR expression in the context of AICD SUMOylation. Different EGFP-tagged AICD plasmids and FLAG-HDAC1 plasmid were co-transfected into Neuro2A cells and the cell lysates were subjected to western blot analysis of NEP and TTR expression. The results revealed that overexpression of AICD consistently increased the expression levels of both NEP and TTR compared with the control group, but that this effect was diminished by HDAC1 co-expression. Moreover, NEP and TTR expression levels were more substantially decreased when EGFP-AICDK43R plasmid and FLAG-HDAC1 plasmid were co-transfected. However, they were significantly rescued by co-transfection of the EGFP-AICD-SUMO1 fusion plasmid and FLAG-HDAC1 plasmid (Figures 3H and 3I). These results indicate that HDAC1 competes with AICD in AICD-mediated regulation of NEP and TTR expression, and that SUMOylated AICD produces a stronger effect than does WT AICD in these regulations.

AICD SUMOylation Increases AICD Association with CREB and p65, and It Increases CREB Binding to the *NEP* Promoter and p65 Binding to the *TTR* Promoter

The above results from immunofluorescence staining and coIP experiments indicate that when AICD is SUMOylated, the association between AICD and Fe65 is increased, presumably for the purpose of transcriptional regulation; however, whether the association between SUMOylated AICD and specific transcription factors is also increased remains unclear. To investigate this issue, we first examined the promoter sequence of the *NEP* gene and found that the *NEP* promoter contains the CRE element specific for CREB binding (Figure 4A, upper panel). We then transfected different EGFP-tagged AICD plasmids to the rat CA1 area and carried out a chromatin immunoprecipitation (ChIP) assay. The results revealed that transfection of EGFP-AICD increases CREB binding to the *NEP* promoter compared with the control group. The binding intensity was decreased by EGFP-AICDK43R transfection and enhanced by EGFP-AICD-SUMO1 transfection (Figures 4A and 4B). We also analyzed the

results of (A) ($F_{3,8} = 106.72$, $p < 0.001$). (C) The position of the NF-κB binding site on the *TTR* gene promoter is shown. Different EGFP-AICD plasmids were transfected into the rat CA1 area, and a ChIP assay for p65 binding to the *TTR* promoter in the hippocampus is shown. coIP using anti-EGFP antibody was conducted to confirm the expression of various AICD plasmids. (D) Quantified results of (C) ($F_{3,8} = 74.87$, $p < 0.001$). Experiments are in three repeats for (A) and (C). (E) Different EGFP-AICD plasmids were transfected into the rat CA1 area. Cell lysate was immunoprecipitated with anti-EGFP antibody and immunoblotted with anti-CREB and anti-p65 antibodies. Cell lysate was also immunoblotted with anti-EGFP antibody to confirm the transfection and expression of various AICD plasmids. (F) Quantified results of AICD association with CREB are shown in the left panel ($F_{3,12} = 174.12$, $p < 0.001$), and those for AICD association with p65 are shown in the right panel ($F_{3,12} = 55.19$, $p < 0.001$). Experiments are in four repeats for (E). (G) Different EGFP-tagged AICD plasmids were transfected into Neuro2A cells. The cell lysates were immunoprecipitated with anti-HDAC1 antibody and immunoblotted with anti-CREB as well as anti-p65 antibodies. Cells lysates were also subjected to western blot determination of NEP and TTR expression. Western blotting with anti-EGFP antibody was conducted to confirm the transfection and expression of various AICD plasmids. (H) Quantified results of (G) are shown ($F_{3,12} = 80.03$ for CREB/HDAC1 and $F_{3,12} = 82.55$ for p65/HDAC1; $F_{3,12} = 66.32$ for NEP and $F_{3,12} = 215.51$ for TTR, all $p < 0.001$). (I) The same cell lysates from (E) were used for the determination of NEP and TTR expression (n = 4). (J) The quantified results of NEP ($F_{3,12} = 58.78$, $p < 0.001$) and TTR ($F_{3,12} = 77.29$, $p < 0.001$) expression are shown. Data are expressed as individual values and mean ± SEM. *$p < 0.05$, **$p < 0.01$, $^{\#}p < 0.001$.

TTR promoter, finding that it contains the nuclear factor κB (NF-κB) binding element specific for p65 (a subunit of NF-κB) binding (Figure 4C, upper panel). We similarly transfected different EGFP-tagged AICD plasmids to the rat CA1 area and carried out a ChIP assay. The results revealed that transfection of EGFP-AICD increases p65 binding to the *TTR* promoter compared with the control group. The binding intensity was decreased by EGFP-AICDK43R transfection and increased by EGFP-AICD-SUMO1 transfection (Figures 4C and 4D). Next, we carried out coIP experiments to examine the association between AICD and CREB as well as that between AICD and p65. Different EGFP-tagged AICD plasmids were transfected into the rat CA1 area. The cell lysates were immunoprecipitated with anti-EGFP antibody and immunoblotted with anti-CREB and anti-p65 antibody. The results revealed that AICD is associated with both CREB and p65. These associations are apparently reduced by EGFP-AICDK43R transfection and are markedly enhanced by EGFP-AICD-SUMO1 transfection (Figures 4E and 4F). Cell lysates were also immunoprecipitated and immunoblotted with anti-EGFP antibody to confirm the transfection and expression of various EGFP-tagged plasmids (Figure 4E, lower panel).

Next, we examined the contribution of HDAC1 in AICD- and AICD SUMOylation-induced NEP and TTR expression in the context of CREB- and p65-mediated transcriptional regulation. In analyzing HDAC1 and CREB interaction as well as HDAC1 and p65 interaction in response to AICD SUMOylation, different EGFP-tagged AICD plasmids were transfected into Neuro2A cells. Cell lysates were immunoprecipitated with anti-HDAC1 antibody and immunoblotted with anti-CREB, anti-p65, and anti-HDAC1 antibodies. The results revealed that the interaction between HDAC1 and CREB decreases upon EGFP-AICDWT transfection compared with the control group. It is further decreased upon EGFP-AICD-SUMO1 transfection, but it is markedly increased by EGFP-AICDK43R transfection (Figures 4G and 4H, left and middle panels). We also analyzed NEP and TTR expression from the same cell lysates in the same context. The results indicated that overexpression of EGFP-AICDWT increased both NEP and TTR expression compared with the control group. This effect was further enhanced by EGFP-AICD-SUMO1 transfection, but was significantly diminished by EGFP-AICDK43R transfection (Figures 4G and 4H, right panel). Taken together, these results revealed that AICD SUMOylation decreased the association between HDAC1 and CREB as well as between HDAC1 and p65, resulting in increased NEP and TTR expression.

Because the *NEP* promoter contains the CRE element for CREB binding and the *TTR* promoter contains the NF-κB binding site for p65 binding, and transfection of the SUMO form of AICD increases the association between AICD and CREB as well as that between AICD and p65, we next examined whether SUMOylated AICD indeed increases the expression of NEP and TTR in the hippocampus. The same cell lysates from Figure 4E were subjected to western blot analysis of NEP and TTR expression. The results showed that transfection of EGFP-AICDWT increased, whereas transfection of EGFP-AICDK43R decreased, the expression of both NEP and TTR in the hippocampus. However, transfection of EGFP-AICD-SUMO1 plasmid more substantially increased NEP and TTR expression compared with EGFP-AICDWT transfection (Figures 4I and 4J). Above we showed that AICD and SUMOylated AICD compete with HDAC1 in the regulation of NEP and TTR expression in Neuro2A cells (Figures 3H and 3I). The present results further indicate that AICD is sufficient to drive the expression of NEP and TTR in hippocampal neurons in the absence of HDAC1 and that this effect is enhanced by AICD SUMOylation.

Lentiviral AICD-SUMO1 Transduction Decreases the Amount of Aβ and Amyloid Plaques and Rescues Spatial Memory Impairment in APP/PS1 Mice

The above results showed that SUMOylation of AICD increases its association with CREB and p65 and enhances the expression of NEP and TTR. Because NEP and TTR both degrade Aβ, we expected that AICD SUMOylation would decrease the amount of Aβ and amyloid plaques. To examine this issue, co-expression vectors containing different lentivirus (lenti-)FLAG-tagged AICD (or lenti-FLAG) and EGFP were transduced into the hippocampus of APP/PS1 mice (8–9 months old) and the Aβ level was determined by western blotting 2 weeks later. The construct of the lenti-FLAG-AICD and EGFP co-expression vector is shown in the upper-right panel of Figure 5A. The results indicated that Aβ was present in the hippocampus of APP/PS1 mice, but not in WT mice. Transduction of the lenti-FLAG-AICDWT vector to APP/PS1 mice reduced the amount of Aβ, but transduction of the lenti-FLAG-AICDK43R vector to APP/PS1 mice increased the amount of Aβ compared with APP/PS1 mice receiving lenti-FLAG-vector transduction. Moreover, transduction of the lenti-FLAG-AICD-SUMO1 vector to APP/PS1 mice further decreased the amount of Aβ compared with APP/PS1 mice receiving lenti-FLAG-AICDWT vector transduction (Figures 5A and 5B, upper panel). Because Aβ can be degraded by NEP and TTR, we also determined NEP and TTR levels in these animals. Western blotting results revealed that the NEP level was decreased in APP/PS1 mice compared with WT mice. This reduction was significantly rescued in APP/PS1 mice receiving lenti-FLAG-AICDWT transduction, but worsened in APP/PS1 mice receiving lenti-FLAG-AICDK43R transduction. However, NEP expression was completely rescued in APP/PS1 mice receiving lenti-FLAG-AICD-SUMO1 transduction. Similar results were also found for TTR expression (Figures 5A and 5B, lower panel). Next, we examined whether AICD SUMOylation produces a similar effect on amyloid plaque deposits. The same lenti-FLAG-AICD vectors as those used above were transduced into a different batch of APP/PS1 mice (8–9 months old), and amyloid plaques were examined using Proteostat dye staining 2 weeks later. The results obtained were similar to those for Aβ accumulation. Apparent amyloid plaque deposits were observed in APP/PS1 mice (indicated by arrows), but not in WT mice. This was reduced by lenti-FLAG-AICDWT transduction but was increased by lenti-FLAG-AICDK43R transduction. Lenti-FLAG-AICD-SUMO1 transduction further decreased the amount of amyloid plaques compared with lenti-FLAG-AICDWT transduction (Figures 5C and 5D).

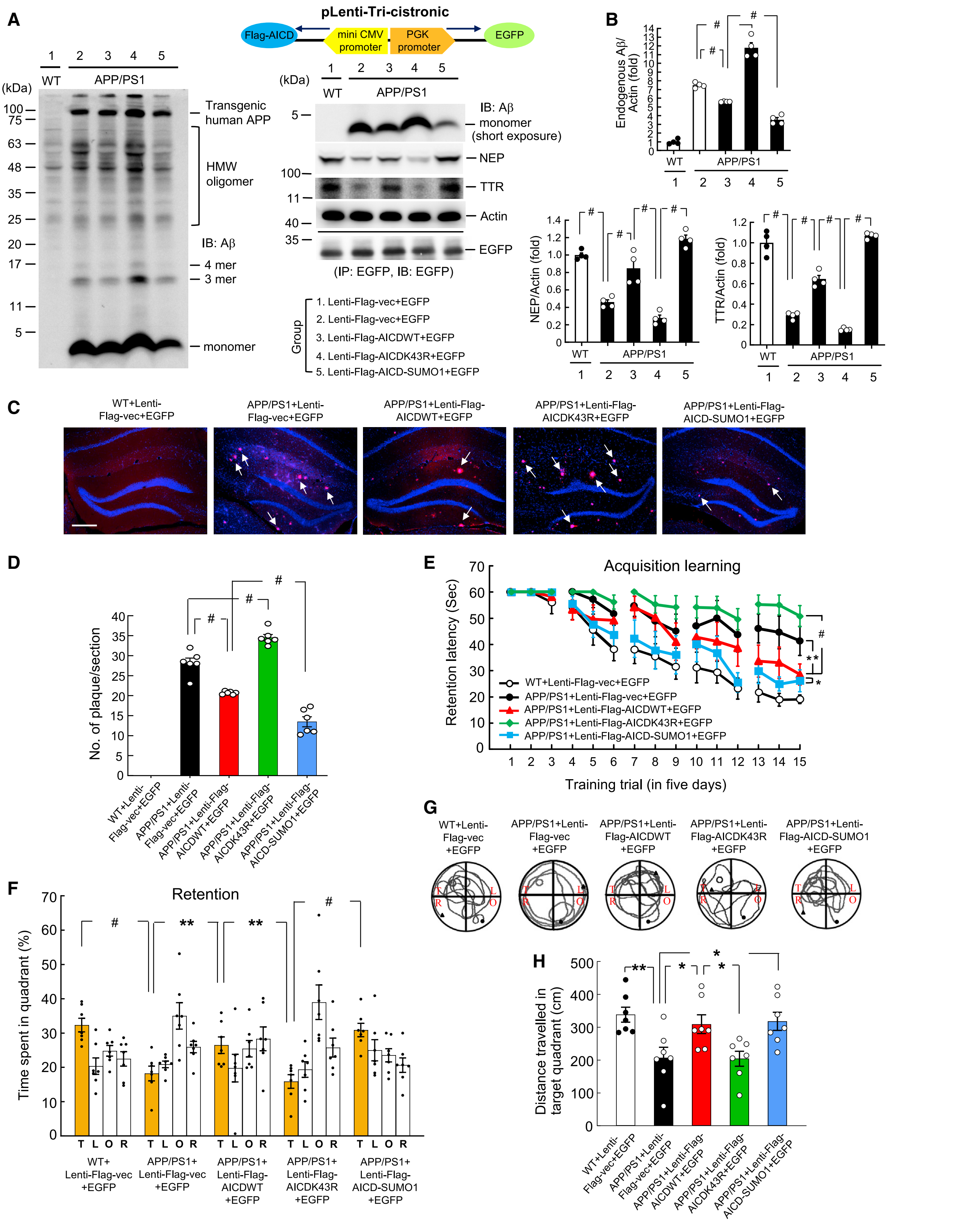

(legend on next page)

Next, we examined the functional significance of AICD SUMOylation. To address this issue, we similarly transduced different lenti-FLAG-AICD vectors into APP/PS1 mice (8–9 months old) and, 2 weeks later, subjected the mice to a water maze learning test. The results revealed that APP/PS1 mice showed significant impairment of acquisition learning compared with WT mice. This impairment was partially rescued by lenti-FLAG-AICDWT transduction, but was worsened by lenti-FLAG-AICDK43R transduction. However, transduction of lenti-FLAG-AICD-SUMO1 vector to APP/PS1 mice further rescued this impairment compared with APP/PS1 mice receiving lenti-FLAG-AICDWT transduction (Figure 5E). Retention performance (probe trial) was assessed in these animals 24 h later. Results for the retention measures "time spent in the target quadrant" (Figure 5F) and "distance travelled in the target quadrant" (Figure 5H) of these animals paralleled the results of their acquisition learning performance (Figure 5E). However, the swim speeds of these animals were similar (Figure S2). Representative swim patterns from the probe trial test for each group of mice are shown in Figure 5G.

Melatonin Increases AICD SUMOylation and Increases Expression of PIAS1, NEP, and TTR in Rats

In this experiment, we aimed to identify an endogenous stimulus that regulates AICD SUMOylation. Melatonin is a pineal hormone whose levels are high in individuals during puberty and decline in aged people, suggesting that it may play a role in aging-related neurodegenerative diseases.[26] More closely related to the present study, melatonin levels have been shown to be lower in AD patients than in age-matched controls.[27] Furthermore, the progression of AD pathology is paralleled by a decline in cerebrospinal fluid melatonin levels.[28] Alternatively, studies of transgenic animal models of AD have suggested that melatonin alleviates the pathology of AD and increases survival.[29,30] In this study, we examined whether melatonin exerts its protective effect against AD through enhanced SUMOylation of AICD. Rats were divided into two groups, one receiving ethanol and the other melatonin infusion into the hippocampal CA1 area. They were sacrificed 1 h after infusion and their CA1 tissue was dissected out and subjected to AICD SUMOylation determination. The results revealed that acute melatonin injection significantly increased the level of AICD SUMOylation (Figures 6A and 6B). Melatonin also apparently increased the SUMOylation of APP C-terminal fragments (Figure 6A). Given our results presented above, showing that AICD is SUMO-modified by PIAS1 and that AICD SUMOylation increases NEP and TTR expression, we speculated that melatonin infusion should also increase the expression of PIAS1, NEP, and TTR. To examine this issue, cell lysates from the same animals were subjected to western blot determination of these three proteins. The results revealed that melatonin infusion markedly increased the expression levels of PIAS1, NEP, and TTR (Figures 6C and 6D). We also examined whether melatonin receptor agonists produce the same effect. Rats were randomly divided into two groups, one receiving DMSO and the other agomelatine injection to the hippocampal CA1 area, and the expression levels of PIAS1, NEP, and TTR were determined by western blotting 1 h later. The results revealed that, similar to what was observed for melatonin, agomelatine increased the expression levels of PIAS1, NEP, and TTR (Figures S3A and S3B). Next, we investigated the mechanism mediating the acute effect of melatonin on these measures. First, we examined whether these effects are mediated through melatonin receptors. A separate batch of rats was randomly divided into three groups receiving DMSO+ethanol (EtOH), DMSO+melatonin, and luzindole+melatonin injections, respectively, into their CA1 area. The two injections were separated by 45 min. The expression levels of PIAS1, NEP, and TTR were determined by western blotting 1 h after the second injection. The results revealed that melatonin injection consistently increased the levels of PIAS1, NEP, and TTR, but that these increases were completely blocked by prior injection of the melatonin receptor antagonist luzindole (Figures 6E and 6F). We further investigated the melatonin receptor-mediated neuronal signaling suggested by these findings. Based on previous results showing that melatonin alleviates AD through enhanced nonamyloidogenic processing of APP and ADAM10 expression via activation of the mitogen-activated protein kinase (MAPK)/extracellular signal-regulated kinase (ERK) signaling pathway,[30] we examined whether MAPK/ERK activation also mediates the effect of melatonin on PIAS1 expression. Rats were randomly divided into three groups, receiving DMSO+ethanol, DMSO+melatonin, and U0126+melatonin injections, respectively, to the CA1 area. The interval between the two injections was 30 min. Animals were sacrificed 1 h

Figure 5. Lentiviral AICD-SUMO1 Transduction Decreases the Amount of Aβ and Amyloid Plaques and Rescues Spatial Memory Impairment in APP/PS1 Mice

(A) Co-expression vectors containing different lenti-FLAG-AICDs and EGFP were transduced into the hippocampus of each APP/PS1 mouse (8–9 months old), and the amounts of Aβ and Aβ oligomers and NEP and TTR expression were examined by western blot 2 weeks later (n = 4). The construct of the lenti-FLAG-AICD and EGFP co-expression vector is shown in the upper-right panel. The EGFP bands confirm the transduction and expression of these lenti-vectors. The descriptions of the various lenti-FLAG-AICD and EGFP co-expression vectors that were transduced into each APP/PS1 mouse are shown in the lower-right panel. HMW, high molecular weight. (B) Quantified results of (A) ($F_{4,15} = 183.67$, $p < 0.001$ for endogenous Aβ, $F_{4,15} = 52.69$, $p < 0.001$ for NEP, and $F_{4,15} = 161.99$, $p < 0.001$ for TTR). The significance levels of separate sets of comparisons are shown in the figure. (C) A different batch of APP/PS1 mice received the same lenti-FLAG-AICD and EGFP co-expression vector transductions as described above, and amyloid plaque deposits (red) examined by Proteostat dye staining are shown in the hippocampus 2 weeks later. DAPI staining is shown in blue (n = 3, two tissue slices for each animal). Scale bar represents 200 μm. (D) Quantified results of (C) ($F_{3,20} = 92.09$, $p < 0.001$). The significance levels of separate sets of comparisons are shown in the figure. (E) Another batch of APP/PS1 mice (and WT mice) received the same lenti-FLAG-AICD and EGFP co-expression vector transductions as described above, and they were subjected to spatial learning 2 weeks later (n = 7). Their acquisition performance is shown ($F_{4,30} = 30.66$, $p < 0.001$). (F) Retention performance (probe trial) of time spent in the target quadrant of these animals (for target region, $F_{4,30} = 12.34$, $p < 0.001$). (G) Representative swim patterns from the probe trial test of each group. (H) Distance travelled in the target quadrant for the probe trial test of these animals ($F_{4,30} = 5.78$, $p < 0.01$). Data are expressed as individual values and mean ± SEM. For (E), data are expressed as mean ± SEM. $^{*}p < 0.05$, $^{**}p < 0.01$, $^{\#}p < 0.001$.

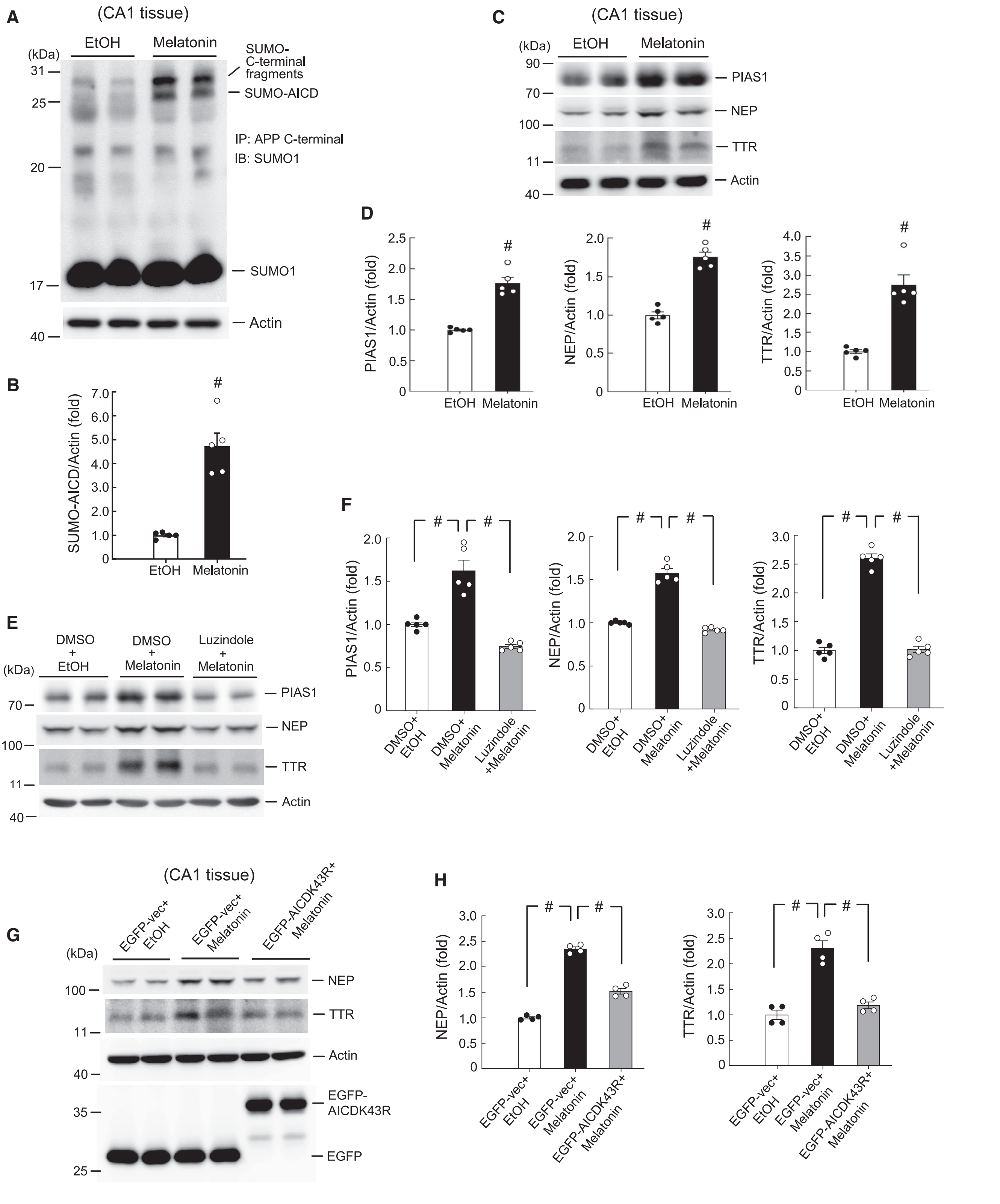

(legend on next page)

after the second injection, and their CA1 tissue was subjected to western blot determination of PIAS1, phosphorylated (p-)ERK1/2, and ERK1/2 expression. The results revealed that acute melatonin injection consistently increased PIAS1 expression and markedly increased the phosphorylation level of ERK1/2, with both of these effects being blocked by prior administration of the MAPK kinase (MEK) inhibitor U0126, which prevents the activation of ERK1/2 (Figures S4A and S4B). The ERK1/2 expression level remained unchanged. These results indicate that melatonin receptor-mediated MAPK/ERK neuronal signaling plays a critical role in PIAS1 expression and in counteracting Aβ toxicity. Lastly, we addressed the issue of whether AICD SUMOylation mediates the effect of melatonin on NEP and TTR expression. Rats were divided into three groups, receiving EGFP-vector transfection+PBS injection, EGFP-vector transfection+melatonin injection, and EGFP-AICDK43R transfection+melatonin injection, respectively, to the CA1 area. Rats were sacrificed 1 h after the second injection and their CA1 tissue was subjected to western blot determination of NEP and TTR expression. The results revealed that melatonin consistently increased the expression of NEP and TTR, but blockade of AICD SUMOylation suppressed the effect of melatonin on NEP and TTR expression (Figures 6G and 6H).

Melatonin Rescues Reduction in AICD SUMOylation, PIAS1, NEP, and TTR Expression in APP/PS1 Mice

In this series of experiments, we examined whether endogenous AICD SUMOylation, PIAS1, NEP, and TTR expression are lower in APP/PS1 mice than in WT mice, and whether melatonin treatment could rescue these deficits. Melatonin or ethanol was directly injected into the CA1 area of mice. For the first experiment, three groups of mice were used: WT mice receiving ethanol injection, APP/PS1 mice receiving ethanol injection, and APP/PS1 mice receiving melatonin injection. Melatonin or ethanol was administered intraperitoneally (i.p.) once per day for 3 weeks. Mice were sacrificed 3 days after the last injection, the frontal cortex tissue was subjected to AICD SUMOylation determination, and hippocampal tissue was subjected to western blot determination of PIAS1, NEP, and TTR expression, as well as Aβ and AICD levels. The results revealed that AICD SUMOylation was significantly decreased in APP/PS1 mice treated with sub-chronic ethanol compared to WT mice treated with sub-chronic ethanol, but sub-chronic melatonin injection completely rescued this deficit (Figures 7A and 7B). Similar results were found with endogenous PIAS1, NEP, and TTR expression in APP/PS1 mice and WT mice treated with sub-chronic ethanol and in APP/PS1 mice treated with sub-chronic melatonin (Figures 7C and 7D). However, the endogenous AICD expression level was increased in both APP/PS1 mice treated with sub-chronic ethanol and APP/PS1 mice treated with sub-chronic melatonin (Figures 7C and 7D).

Because the above results showed that melatonin rescues the reduced NEP and TTR expression in APP/PS1 mice and because NEP and TTR both degrade Aβ, we next asked whether melatonin treatment could reduce Aβ levels in APP/PS1 mice. The frontal cortex tissue from the same animals as described above was used to determine the endogenous Aβ level by western blotting. The results revealed that the levels of both Aβ monomer and oligomers were significantly higher in APP/PS1 mice compared with WT mice, but sub-chronic melatonin injection dramatically decreased the levels of both Aβ monomer and oligomers in APP/PS1 mice (Figures 7E and 7F).

DISCUSSION

In the present study of the role of AICD SUMOylation in the context of AD, we found that AICD SUMOylation functions as an endogenous protection mechanism against Aβ toxicity in APP/PS1 mice and mediates the neuroprotective effect of melatonin against AD through enhanced degradation of Aβ. In addition, we found that AICD is SUMO-modified by PIAS1 in both HEK293T cells and the brain. Multiple, although in some cases indistinct, AICD-SUMO bands were observed in cells, with AICD SUMOylation at Lys-43 giving rise to the most prominent band. This result suggests that there may be other SUMO residues on AICD in cells. Whether mutations of these residues have an additive effect on blockade of AICD SUMOylation and NEP and TTR expression requires further investigation. Alternatively, Lys-43 seems to be the predominant, if not the only, SUMO residue on AICD in the hippocampus, and mutation at Lys-43 completely abolished the biochemical, physiological, and behavioral effects of AICD SUMOylation. The difference regarding candidate AICD SUMO residues between cell lines and the brain also needs to be clarified. Our results showed that AICD is co-localized with PIAS1 and SUMO-modified by PIAS1 endogenously in hippocampal neurons. Our results further revealed that PIAS1 is also associated with the C83 and C99 fragments, in addition to AICD, and that these fragments are endogenously SUMO-modified by PIAS1 as well. This result suggests that PIAS1 SUMOylates both the cleaved AICD and the un-cleaved AICD present in the C83 and C99 fragments, but that PIAS1 SUMOylation of the cleaved AICD

Figure 6. Melatonin Increases PIAS1 Expression and AICD SUMOylation and Increases the Expression of NEP and TTR in Rats

(A) Rats were divided into two groups (n = 5), one receiving ethanol (20%) and the other melatonin (7 μg) injected into the CA1 area. They were sacrificed 1 h later and the AICD SUMOylation level was determined. (B) Quantified results of (A) ($t_{1,8} = 6.7$, $p < 0.001$). (C) Tissue lysates from the same animals were subjected to western blot analysis for the expression of PIAS1, NEP, and TTR. (D) Quantified results of (C) ($t_{1,8} = 8.12$, $p < 0.001$ for PIAS1, $t_{1,8} = 9.77$, $p < 0.001$ for NEP, and $t_{1,8} = 6.52$, $p < 0.001$ for TTR). (E) Another batch of rats was divided into three groups (n = 5) and received DMSO (40%)+ethanol (20%), DMSO+melatonin (7 μg), or luzindole (1 μg)+melatonin (7 μg) injections to their CA1 area. They were sacrificed 1 h after the second injection and their CA1 tissue was subjected to western blot determination of PIAS1, NEP, and TTR expression. (F) Quantified results of (E) ($F_{2,12} = 38.2$ for PIAS1, $F_{2,12} = 141.43$ for NEP, and $F_{2,12} = 227.9$ for TTR, all $p < 0.001$). (G) Rats were divided into three groups (n = 4) and received EGFP-vector transfection+ethanol (20%) injection, EGFP-vector transfection+melatonin injection (7 μg), and EGFP-AICDK43R transfection+melatonin injection (7 μg) into the CA1 area. The interval between these two injections was 24 h. They were sacrificed 1 h after melatonin (or ethanol) injection, and their CA1 tissue was subjected to western blot determination of NEP and TTR expression. (H) Quantified results of (G) ($F_{2,9} = 271.43$, $p < 0.001$ for NEP and $F_{2,9} = 43.8$, $p < 0.001$ for TTR). Data are expressed as individual values and mean ± SEM. $^{\#}p < 0.001$. EtOH, ethanol.

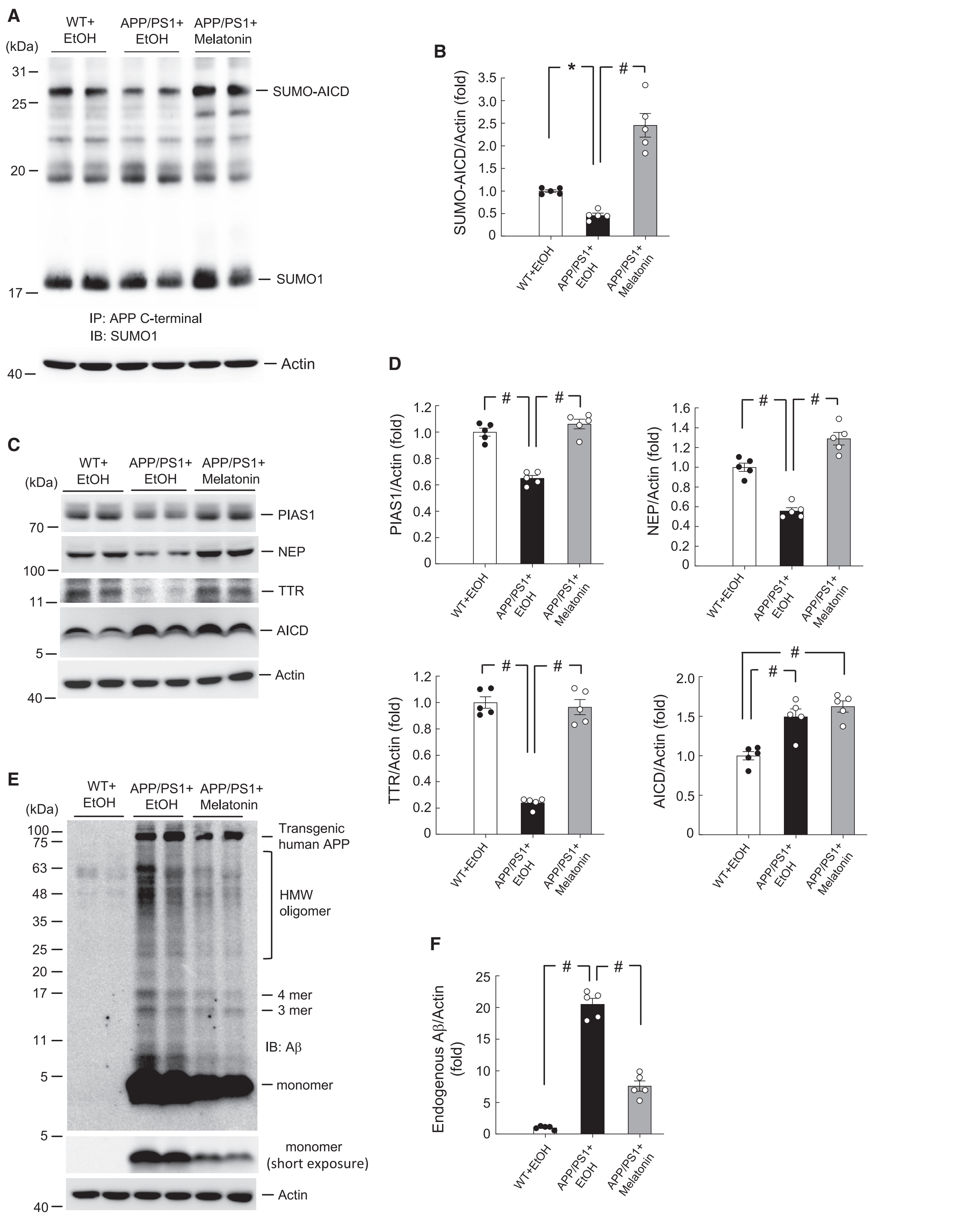

(legend on next page)

takes place in the nucleus because endogenous PIAS1 and AICD are co-localized only in the nucleus (Figure 2D). However, it is unlikely that the C83 and C99 fragments (excluding the AICD fragment) are direct SUMO substrates of PIAS1 because immunohistochemical studies have shown that both the C83 and C99 fragments are localized on the cell membrane.[31] The observed association between PIAS1 and C83/C99 fragments likely arose because, when the coIP experiments were carried out, the cells were disrupted and some PIAS1 moved to the extranuclear area. As a result, PIAS1 is associated with, and SUMO modifies, AICD present on the C83 and C99 fragments in the cytosol area. However, the PIAS1-C83 fragment interaction probably does not affect the nonamyloidogenic processing of APP in the context of AICD SUMOylation because the C83 fragment is a product of α-secretase cleavage of APP; hence, the presence of the C83 fragment per se is a result of the nonamyloidogenic processing of APP, whether it interacts with PIAS1 or not. The physiological significance of the endogenous association between PIAS1 and C83/C99 fragments containing AICD warrants further exploration in future studies. Moreover, the less intense association between PIAS1 and AICD than between PIAS1 and the C83 and C99 fragments is congruent with a previous report showing that AICD is unstable and easily degraded.[6] Furthermore, there is a stronger association between PIAS1 and the C83 fragment than between PIAS1 and the C99 fragment (Figure 2A). This is consistent with the general notion that the nonamyloidogenic pathway is the predominant pathway under physiological conditions. Alternatively, it is unlikely that PIAS1 is associated with Aβ40/42 or the P3 peptide on the C83 and C99 fragments, respectively, because no band with a molecular mass less than 6 kDa (the size of AICD) was observed (Figure 2A).

In an examination of the molecular mechanism of AICD SUMOylation, we found that the association between AICD and Fe65 is increased when AICD is SUMOylated, but that this association is reduced when AICD SUMOylation at Lys-43 is blocked. Furthermore, immunofluorescence staining revealed that SUMOylated AICD was well co-localized with Fe65 in the nucleus only, but that blockade of AICD SUMOylation prevented nuclear translocation of AICD. These findings are likely due to the fact that Lys-43 is located immediately next to the YENPTY motif on AICD, which is the binding domain for Fe65[32] that is necessary for the stabilization and subsequent nuclear translocation of AICD.[3,4] When AICD is SUMOylated, its association with Fe65 is increased (Figure 3A) and nuclear localization of AICD is observed. On the contrary, mutation at Lys-43 may cause a conformational change of AICD that prevents Fe65 binding to AICD and subsequent AICD nuclear translocation. This suggestion is partly supported by our finding that mutation at Lys-43 reduces the stability of AICD (Figure 1E). It is also possible that AICD SUMOylation changes the interaction between AICD and Fe65, given previous results showing that SUMOylation alters protein-protein interactions.[33] Moreover, immunofluorescence experiments revealed that SUMOylated AICD and Fe65 co-localized as puncta in the perichromatin region of Neuro2A neurons. Because the perichromatin region has important functions including transcription,[34] this distribution allows the AICD complex to interact with transcription factors for transcriptional regulation.

In a further study of the mechanism of AICD SUMOylation, we also demonstrated that SUMOylated AICD shows weaker interaction with HDAC1, stronger interaction with CREB and p65, enhanced binding to the *NEP* and *TTR* promoters, and increased expression of NEP and TTR. Conversely, blockade of AICD SUMOylation had opposite effects. These results provide direct evidence supporting the previous speculation that AICD competes with HDAC1 for DNA binding in cells overexpressing APP695.[14] Moreover, our results further demonstrate that SUMOylation of AICD plays a critical role in competing with HDAC1 for interaction with CREB and p65 for transcriptional regulation of NEP and TTR expression. Similarly, in another study we found that SUMOylation of the methyl-CpG-binding protein 2 protein releases CREB from the HDAC1 co-repressor complex for upregulation of brain-derived neurotrophic factor expression.[35] In the present study, an examination of the physiological significance of AICD SUMOylation disclosed that transduction of lenti-FLAG-AICDWT vector to the hippocampus of APP/PS1 mice decreases the amount of Aβ and amyloid plaques and rescues spatial memory deficit in APP/PS1 mice. This result is consistent with reports that AICD plays a protective role against AD.[6,9,14] However, our results are not congruent with reports that AICD is involved in the pathology of AD. For example, AICD59 transgenic mice show tau hyperphosphorylation and impaired working memory.[36] Furthermore, overexpression of AICD57 and AICD59 in PC12 cells was found to induce GSK-3β expression and phosphorylation and produce neurotoxicity.[37] This discrepancy is likely due, at least in part, to the different lengths of the AICD fragments used in these studies. In the present study we used AICD50, which can translocate to the nucleus to regulate gene expression, whereas nuclear translocation of AICD57 and AICD59 has not been reported, and these AICDs may produce their effects in the cytosol area. In addition, we found that, compared with WT AICD, SUMOylated AICD exerts greater effects in reducing Aβ level and amyloid plaque accumulation and in rescuing the spatial memory deficit in APP/PS1 mice. This is probably due to enhanced expression of NEP and TTR by SUMOylated AICD, resulting in

Figure 7. Melatonin Rescues Reduction in AICD SUMOylation, PIAS1, NEP, and TTR Expression in APP/PS1 Mice

(A) Three groups of mice (n = 5) received daily ethanol (20%) or melatonin injections (0.03 ml, i.p.) for 3 weeks: wild-type mice receiving ethanol, APP/PS1 mice receiving ethanol, and APP/PS1 mice receiving melatonin. Mice were sacrificed 3 days after the last injection and their frontal cortex tissue was subjected to AICD SUMOylation determination. (B) Quantified results of (A) ($F_{2,12}$ = 44.6, $p < 0.001$). (C) The hippocampal tissue from the same animals was subjected to western blot determination of PIAS1, NEP, TTR, and AICD levels. (D) Quantified results of (C) ($F_{2,12}$ = 54.24 for PIAS1, $F_{2,12}$ = 59.67 for NEP, $F_{2,12}$ = 101.1 for TTR, and $F_{2,12}$ = 18.53 for AICD, all $p < 0.001$). (E) The same cell lysates used in (C) were subjected to endogenous Aβ (monomer and oligomers) level determination using anti-Aβ antibody by western blot. (F) Quantified results of (E) ($F_{2,12}$ = 188.04, $p < 0.001$). Data are expressed as individual values and mean ± SEM. *$p < 0.05$, $^{\#}p < 0.001$.

more efficient clearance of Aβ; therefore, less severe pathology and cognitive impairment were observed in APP/PS1 mice. This explanation is supported by our finding that blockade of AICD SUMOylation yields more Aβ and amyloid plaques, and it causes more severe impairment of spatial learning and memory, in APP/PS1 mice compared with APP/PS1 mice receiving lenti-FLAG-AICDWT transduction. A possible explanation for these results is that lower NEP and TTR expression leads to less degradation of Aβ, and hence to enhanced AD pathology in APP/PS1 mice. We also examined the effect of AICD SUMOylation on amyloid plaque accumulation in aged APP/PS1 mice (16 months old). The results indicated that AICD SUMOylation has a similar effect in reducing the number of amyloid plaques in aged APP/PS1 mice except that its effect is less dramatic than that observed in 8- to 9-month-old APP/PS1 mice (Figure S5). Our results are consistent with the notion that AICD is involved in nuclear signaling and transcriptional regulation in AD.[1] They are also congruent with the literature indicating that SUMOylation plays a role in the pathogenesis of AD.[38,39] Moreover, there is a report showing that APP can be ubiquitinated at Lys-688 (which corresponds to Lys-43 on AICD),[17] but we found that mutation of AICD at Lys-43 did not affect AICD ubiquitination in the hippocampus (Figure 2H). This difference may be due to whether AICD ubiquitination occurs before or after AICD is cleaved from APP, but this has yet to be verified. There is also a study showing that neddylation occurs at Lys-31 and Lys-43 on AICD,[18] but we found no apparent difference in AICD neddylation between the AICDWT group and AICDK43R group (Figure S6). This discrepancy is likely due to the fact that in the earlier study, both the Lys-31 and Lys-43 residues were mutated, whereas in our study only the Lys-43 residue was mutated. It could be that Lys-31 plays a more important role in AICD neddylation, although other possibilities exist.

In an examination of the endogenous molecule that regulates AICD SUMOylation, we found that melatonin, a pineal gland neurohormone, increases the expression of PIAS1, enhances AICD SUMOylation, and increases the expression of NEP and TTR in the hippocampi of both rats and APP/PS1 mice. These effects would result in more efficient degradation of Aβ (Figure 8), consistent with the literature indicating that melatonin protects against AD.[29,30] In addition, the AICD expression level is increased in both APP/PS1 mice and APP/PS1 mice treated with melatonin. This is an expected result because more AICD is generated in APP/PS1 mice due to amyloidogenic processing of APP by β-secretase and γ-secretase cleavage of APP. Although melatonin has been shown to promote nonamyloidogenic processing of APP,[40] AICD is still generated in APP/PS1 mice treated with melatonin due to α-secretase and γ-secretase cleavage of APP. Furthermore, some evidence has suggested that APP-induced cell death is dependent on the cleavage to AICD. For example, overexpression of APP was found to induce cell death in the *Drosophila* nervous system, but expression of the truncated form of APP lacking the AICD domain failed to induce cell death.[41] Another study showed that knockout of presenilins (PSs), part of the γ-secretase complex, reduces the expression and activity of p53, whereas overexpression of AICD in PS-deficient cells increases p53 reporter activity and p53 mRNA level.[42] However, our results showed that AICD has a protective effect against Aβ toxicity. The difference between our results and the previous findings could be due to differences in the AICD-interacting proteins and AICD-regulated genes examined. We studied the effect of the interaction between AICD and Fe65 on the nuclear translocation of AICD, whereas the other study examined the interaction between AICD and FOXO that mediates apoptosis. Furthermore, we examined AICD-regulated NEP and TTR expression in terms of Aβ degradation, whereas the other study examined AICD-regulated p53 expression that mediates cell death.

The PIAS protein family has several members, but in the present study we concentrated on the role of PIAS1. We took this approach in part because of previous findings showing that PIAS1 expression is induced by neuronal activation,[43] and also in light of previous work showing that PIAS1 SUMOylation of the MeCP2 protein alleviates symptoms of another neurological disorder, Rett syndrome.[35] More closely related to the present study, we recently showed that PIAS1 can also SUMO-modify HDAC1 and Elk-1, and that PIAS1 SUMOylation of these two proteins alleviates pathological symptoms in an animal model of AD. However, PIAS1 SUMO-modifies the various proteins to rescue AD pathology via different, although somewhat overlapping, mechanisms. In the present study, we found that PIAS1 SUMOylation of AICD enhances Aβ degradation. In previous studies, however, PIAS1 SUMOylation of HDAC1 was found to reduce apoptosis,[22] and PIAS1 SUMOylation of Elk-1 was found to promote neuronal survival in APP/PS1 mice.[23] Furthermore, PIAS1 SUMOylation of AICD was strongly upregulated by melatonin, whereas melatonin is implicated in the prevention of AD. The present results further strengthen the importance of AICD SUMOylation as an endogenous prevention strategy against Aβ toxicity. Moreover, it is possible that other PIAS family proteins also SUMO-modify AICD. In addition, we cannot exclude the possibility that AICD could be SUMOylated by other SUMO E3 ligases, such as RanBP2.

In this study, we have demonstrated a novel posttranslational regulation of AICD with SUMO-modification and demonstrated that AICD SUMOylation is a novel defense mechanism protecting against AD pathology by facilitating the degradation of Aβ. We also identified a novel physiological role of melatonin in inducing AICD SUMOylation. These results shed light on a promising therapeutic direction to combat AD.

MATERIALS AND METHODS

Animals

Adult male Sprague-Dawley rats (250–350 g) and C57BL/6 mice were purchased from the BioLASCO and the National Laboratory Animal Center, Taiwan, respectively. The APP/PS1 mice were purchased from Jackson Laboratory (Bar Harbor, ME, USA) (strain name B6.Cg-Tg(APPswe,PSEN1dE9)85Dbo/Mmjax, stock no. 005864). All animals were bred and maintained on a 12-h light/12-h dark cycle (light on at 8:00 AM) at the Animal Facility of the Institute of Biomedical Sciences (IBMS), Academia Sinica with food and water continuously available. Experimental procedures follow the *Guide*

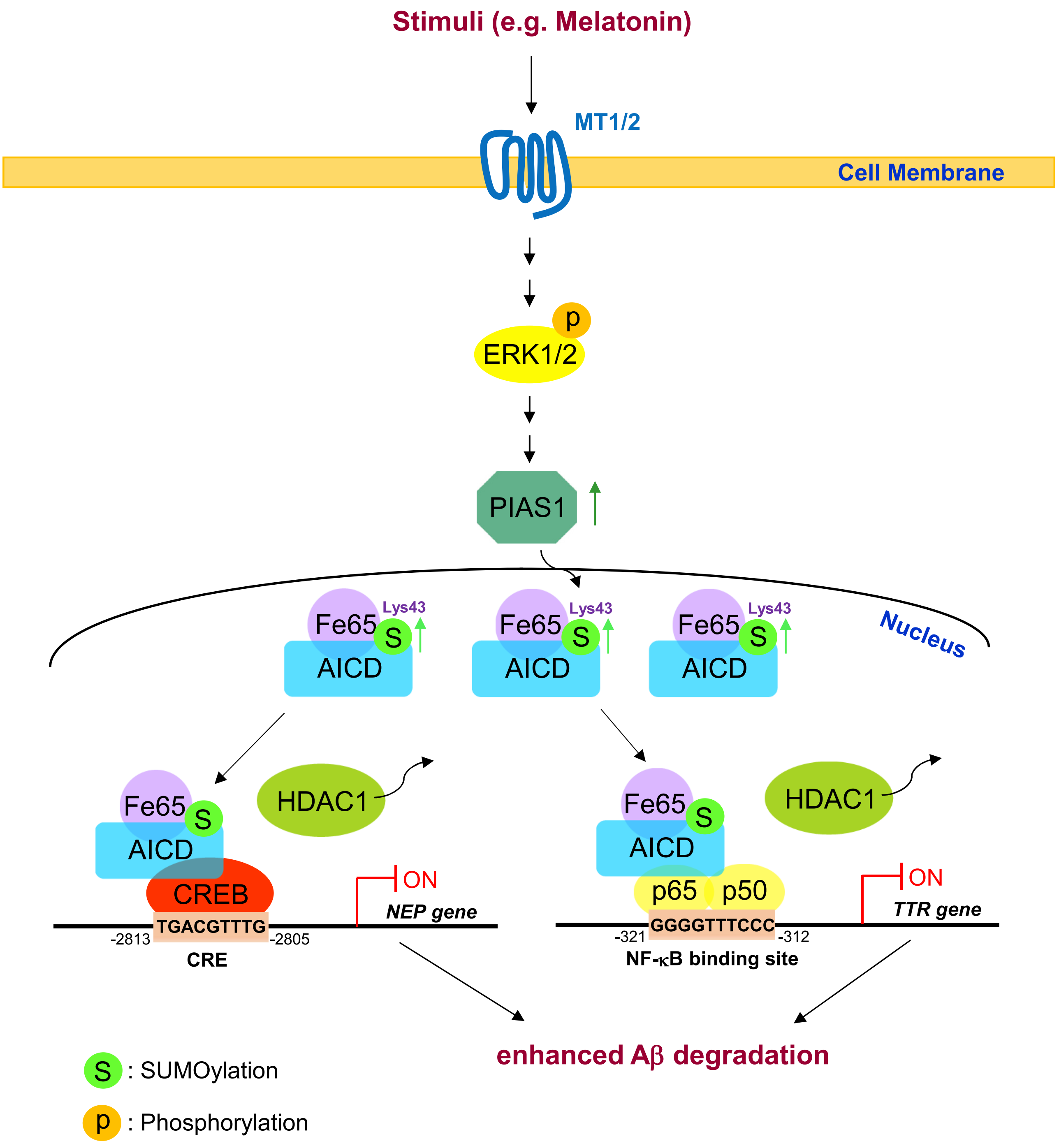

Figure 8. Melatonin Signaling in Protection against AD

The illustration shows the relationship among AICD SUMOylation, AICD association with CREB and p65 in the AICD-Fe65 complex, its effect on CREB binding to the *NEP* gene promoter and p65 binding to the *TTR* gene promoter, as well as NEP and TTR expression for Aβ degradation. Melatonin is shown as an endogenous stimulus to induce AICD SUMOylation, and its neuronal signaling is also shown. MT1/2, melatonin receptor 1/2.

for the Care and Use of Laboratory Animals of the National Institute of Health and were approved by the Animal Committee of IBMS, Academia Sinica.

Plasmid DNA Construction

AICD is the product of γ-secretase cleavage of APP, and different lengths of AICD were identified. AICD50 and AICD51 are the major

C-terminal fragments generated by γ-secretase.[44] Furthermore, AICD50 is more stable than AICD51.[45] We have therefore cloned *AICD50* for the present study. The details of plasmid construction for all genes are described in Supplemental Materials and Methods.

Cell Culture and Plasmid Transfection

HEK293T cells and Neuro2A cells were maintained in Dulbecco's modified Eagle's medium containing 10% fetal bovine serum and incubated at 37°C in a humidified atmosphere with 5% CO_2. Plasmid transfection was made by using the Lipofectamine 2000 reagent (Invitrogen, Carlsbad, CA, USA) in 6- and 12-well culture plates according to the manufacturer's instructions. Immunoprecipitation (IP) and western blot were conducted 48 h after plasmid transfection.

Plasmid DNA and siRNA Transfection to the Hippocampus

Rats were anesthetized with pentobarbital (40 mg/kg, i.p.) and subjected to stereotaxic surgery. EGFP-tagged AICD plasmid DNA was directly injected into the rat CA1 area at a rate of 0.1 μL/min. A total of 0.7 μL was injected into each side of the CA1 area. Plasmid DNA was prepared as described previously.[46] Transient plasmid DNA transfection was conducted using the non-viral transfection agent polyethylenimine (PEI), and we have previously demonstrated that PEI does not produce toxicity to hippocampal neurons.[47] Briefly, plasmid DNA was diluted in 5% glucose to a stock concentration of 2.77 μg/μL. Branched PEI of 25 kDa (Sigma) was diluted to 0.1 mM concentration in 5% glucose and added to the DNA solution. Immediately before injection, 0.1 mM PEI was added to reach a ratio of PEI nitrogen per DNA phosphate equal to 10. The mixture was subjected to vortexing for 30 s and allowed to equilibrate for 15 min. For siRNA injection, 0.7 μL of PIAS1 siRNA (10 pmol), CREB siRNA (10 pmol), or control siRNA was transfected into the rat CA1 area bilaterally also using the transfection agent PEI. The siRNA sequences are described in Supplemental Materials and Methods. The injection needle was left in place for 5 min to limit the diffusion of injected agent. Animals were sacrificed 48 h after plasmid transfection or siRNA injection and their hippocampal tissue was dissected out and subjected to coIP, western blot, and an *in vitro* SUMOylation assay.

Lentiviral Vector Construction and Preparation

For construction of FLAG-AICD, FLAG-AICDK43R, and FLAG-AICD-SUMO1 lentiviral vectors, full-length FLAG-AICD, FLAG-AICDK43R, and FLAG-AICD-SUMO1 fusion plasmids were sub-cloned into the lentiviral vector pLenti-Tri-cistronic (ABM, Richmond, BC, Canada) by amplifying different FLAG-AICD non-viral constructs with different primes. The primer sequences and detailed procedures are described in Supplemental Materials and Methods. The EGFP sequence was cloned into the pLenti-vector, pLenti-FLAG-AICD, pLenti-FLAG-AICDK43R, and pLenti-FLAG-AICD-SUMO1 vectors to obtain a cistronic co-expressing vector. The primer sequences for EGFP and lentivirus packaging procedures are detailed in Supplemental Materials and Methods. The final concentration of the lentiviral vector used for injection to the brain is 5×10^8 IU/mL.

ChIP Assay

A ChIP assay was performed according to the protocol of the Millipore ChIP assay kit (catalog no. 17-10085). The ChIP assay was carried out in the rat hippocampus for determination of CREB binding to the *NEP* promoter and p65 binding to the *TTR* promoter upon various EGFP-tagged AICD plasmid transfections to rat CA1 area. The detailed procedures are described in Supplemental Materials and Methods.

In Vitro SUMOylation Assay for the CA1 Tissue

Hippocampal CA1 tissue lysate was prepared in the same way as that prepared for western blot. For the IP experiment, the clarified lysate (0.5 mg) was immunoprecipitated with 3 μL of anti-EGFP antibody at 4°C overnight. The protein A agarose beads (30 mL, 50% slurry, GE Healthcare, IL, USA) were added to the IP reaction product to catch the immune complex at 4°C for 3 h. The immune complex on beads was washed three times with washing buffer containing 20 mM HEPES (pH 7.4), 150 mM NaCl, 1 mM EDTA, 1% IGEPAL CA-630, 1 mM DTT, 50 mM β-glycerophosphate, 50 mM NaF, 10 mg/mL PMSF, 4 mg/mL aprotinin, 4 mg/mL leupeptin, and 4 mg/mL pepstatin and subjected to *in vitro* SUMOylation reaction with the addition of the recombinant PIAS1 protein (3 μL, catalog no. BML-UW9960, Enzo Life Sciences, Farmingdale, NY, USA), E1 (1 μL), E2 (1 μL), and the SUMO1 (0.5 μL) proteins provided in the kit. An *in vitro* SUMOylation assay was performed using the SUMOlink kit according to the manufacturer's instructions (Active Motif, CA, USA) and boiled in Laemmli sample buffer at 95°C for 10 min. The SUMOylation reaction product was subjected to 10% SDS-PAGE and transferred onto the polyvinylidene fluoride (PVDF) membrane. The membrane was immunoblotted with anti-SUMO1 antibody (1:3,000; Active Motif) or anti-EGFP antibody (1:8,000; Sigma-Aldrich, catalog no. 11814460001). For determination of endogenous AICD SUMOylation after PIAS1 siRNA transfection, the clarified lysate (0.5 mg) was immunoprecipitated with 3 μL of anti-APP C-terminal fragment antibody (BioLegend, San Diego, CA, USA, catalog no. 802801) at 4°C overnight. The protein A agarose beads (30 mL, 50% slurry, GE Healthcare, IL, USA) were added to the IP reaction product to catch the immune complex at 4°C for 3 h. The immune complex on beads was washed three times with washing buffer and recombinant E1, E2, and SUMO1 (but no PIAS1) proteins were added to the IP reaction product. The remaining procedures were the same as those described above.

Statistical Analysis

Spatial acquisition (escape latency) data were analyzed with two-way analysis of variance (ANOVA) with repeated-measure followed by a post hoc Newman-Keuls multiple comparison test (represented by q value). Retention performance data and biochemical data were analyzed with the Student's t test or one-way ANOVA followed by Newman-Keuls comparisons. Values of $p < 0.05$ were considered statistically significant ($^*p < 0.05$, $^{**}p < 0.01$, $^{\#}p < 0.001$).

SUPPLEMENTAL INFORMATION

Supplemental Information can be found online at https://doi.org/10.1016/j.ymthe.2020.09.003.

AUTHOR CONTRIBUTIONS

Y.-C.L. designed several experiments and wrote part of the manuscript. Y.-C.L. and W.-L.H. performed most of the experiments and analyzed the data. Y.-L.M. helped with animal surgery and behavioral experiments as well as data analysis. E.H.Y.L. designed the experiments, guided and supervised the project, solved problems, and wrote most parts of the manuscript.

CONFLICTS OF INTEREST

The authors declare no competing interests.

ACKNOWLEDGMENTS

This work was supported by grants from the Ministry of Science and Technology (MOST108-2320-B-001-005), Academia Sinica (AS-TP-109-L07), and from IBMS (IBMS-CRC107-P04), Academia Sinica in Taiwan. Thanks are given to the Animal Core and Confocal Core Facilities of IBMS. The original blots for all western blot experiments are shown in Figure S7.

REFERENCES

1. Bukhari, H., Glotzbach, A., Kolbe, K., Leonhardt, G., Loosse, C., and Müller, T. (2017). Small things matter: Implications of APP intracellular domain AICD nuclear signaling in the progression and pathogenesis of Alzheimer's disease. Prog. Neurobiol. *156*, 189–213.
2. Wilquet, V., and De Strooper, B. (2004). Amyloid-beta precursor protein processing in neurodegeneration. Curr. Opin. Neurobiol. *14*, 582–588.
3. Kimberly, W.T., Zheng, J.B., Guénette, S.Y., and Selkoe, D.J. (2001). The intracellular domain of the β-amyloid precursor protein is stabilized by Fe65 and translocates to the nucleus in a Notch-like manner. J. Biol. Chem. *276*, 40288–40292.
4. Cao, X., and Südhof, T.C. (2001). A transcriptionally [correction of transcriptively] active complex of APP with Fe65 and histone acetyltransferase Tip60. Science *293*, 115–120.
5. Chow, V.W., Mattson, M.P., Wong, P.C., and Gleichmann, M. (2010). An overview of APP processing enzymes and products. Neuromolecular Med. *12*, 1–12.
6. Belyaev, N.D., Kellett, K.A., Beckett, C., Makova, N.Z., Revett, T.J., Nalivaeva, N.N., Hooper, N.M., and Turner, A.J. (2010). The transcriptionally active amyloid precursor protein (APP) intracellular domain is preferentially produced from the 695 isoform of APP in a β-secretase-dependent pathway. J. Biol. Chem. *285*, 41443–41454.
7. Pardossi-Piquard, R., Petit, A., Kawarai, T., Sunyach, C., Alves da Costa, C., Vincent, B., Ring, S., D'Adamio, L., Shen, J., Müller, U., et al. (2005). Presenilin-dependent transcriptional control of the Aβ-degrading enzyme neprilysin by intracellular domains of βAPP and APLP. Neuron *46*, 541–554.
8. Beckett, C., Nalivaeva, N.N., Belyaev, N.D., and Turner, A.J. (2012). Nuclear signalling by membrane protein intracellular domains: the AICD enigma. Cell. Signal. *24*, 402–409.
9. Belyaev, N.D., Nalivaeva, N.N., Makova, N.Z., and Turner, A.J. (2009). Neprilysin gene expression requires binding of the amyloid precursor protein intracellular domain to its promoter: implications for Alzheimer disease. EMBO Rep. *10*, 94–100.
10. Grimm, M.O.W., Mett, J., Stahlmann, C.P., Grösgen, S., Haupenthal, V.J., Blümel, T., Hundsdörfer, B., Zimmer, V.C., Mylonas, N.T., Tanila, H., et al. (2015). APP intracellular domain derived from amyloidogenic β- and γ-secretase cleavage regulates neprilysin expression. Front. Aging Neurosci. *7*, 77.
11. Liz, M.A., Mar, F.M., Franquinho, F., and Sousa, M.M. (2010). Aboard transthyretin: from transport to cleavage. IUBMB Life *62*, 429–435.
12. Costa, R., Ferreira-da-Silva, F., Saraiva, M.J., and Cardoso, I. (2008). Transthyretin protects against A-beta peptide toxicity by proteolytic cleavage of the peptide: a mechanism sensitive to the Kunitz protease inhibitor. PLoS ONE *3*, e2899.
13. Silva, C.S., Eira, J., Ribeiro, C.A., Oliveira, Â., Sousa, M.M., Cardoso, I., and Liz, M.A. (2017). Transthyretin neuroprotection in Alzheimer's disease is dependent on proteolysis. Neurobiol. Aging *59*, 10–14.
14. Kerridge, C., Belyaev, N.D., Nalivaeva, N.N., and Turner, A.J. (2014). The Aβ-clearance protein transthyretin, like neprilysin, is epigenetically regulated by the amyloid precursor protein intracellular domain. J. Neurochem. *130*, 419–431.
15. Lee, M.S., Kao, S.C., Lemere, C.A., Xia, W., Tseng, H.C., Zhou, Y., Neve, R., Ahlijanian, M.K., and Tsai, L.H. (2003). APP processing is regulated by cytoplasmic phosphorylation. J. Cell Biol. *163*, 83–95.
16. Morel, E., Chamoun, Z., Lasiecka, Z.M., Chan, R.B., Williamson, R.L., Vetanovetz, C., Dall'Armi, C., Simoes, S., Point Du Jour, K.S., McCabe, B.D., et al. (2013). Phosphatidylinositol-3-phosphate regulates sorting and processing of amyloid precursor protein through the endosomal system. Nat. Commun. *4*, 2250.
17. El Ayadi, A., Stieren, E.S., Barral, J.M., and Boehning, D. (2012). Ubiquilin-1 regulates amyloid precursor protein maturation and degradation by stimulating K63-linked polyubiquitination of lysine 688. Proc. Natl. Acad. Sci. USA *109*, 13416–13421.
18. Lee, M.R., Lee, D., Shin, S.K., Kim, Y.H., and Choi, C.Y. (2008). Inhibition of APP intracellular domain (AICD) transcriptional activity via covalent conjugation with Nedd8. Biochem. Biophys. Res. Commun. *366*, 976–981.
19. Zhang, Y.Q., and Sarge, K.D. (2008). Sumoylation of amyloid precursor protein negatively regulates Aβ aggregate levels. Biochem. Biophys. Res. Commun. *374*, 673–678.
20. Johnson, E.S. (2004). Protein modification by SUMO. Annu. Rev. Biochem. *73*, 355–382.
21. Hay, R.T. (2005). SUMO: a history of modification. Mol. Cell *18*, 1–12.
22. Tao, C.C., Hsu, W.L., Ma, Y.L., Cheng, S.J., and Lee, E.H.Y. (2017). Epigenetic regulation of HDAC1 SUMOylation as an endogenous neuroprotection against Aβ toxicity in a mouse model of Alzheimer's disease. Cell Death Differ. *24*, 597–614.
23. Liu, S.Y., Ma, Y.L., Hsu, W.L., Chiou, H.Y., and Lee, E.H.Y. (2019). Protein inhibitor of activated STAT1 Ser^{503} phosphorylation-mediated Elk-1 SUMOylation promotes neuronal survival in APP/PS1 mice. Br. J. Pharmacol. *176*, 1793–1810.
24. Zhao, Q., Xie, Y., Zheng, Y., Jiang, S., Liu, W., Mu, W., Liu, Z., Zhao, Y., Xue, Y., and Ren, J. (2014). GPS-SUMO: a tool for the prediction of sumoylation sites and SUMO-interaction motifs. Nucleic Acids Res. *42*, W325–W330.
25. Chen, Y.C., Hsu, W.L., Ma, Y.L., Tai, D.J., and Lee, E.H.Y. (2014). CREB SUMOylation by the E3 ligase PIAS1 enhances spatial memory. J. Neurosci. *34*, 9574–9589.
26. Reiter, R.J., Tan, D.X., Poeggeler, B., Menendez-Pelaez, A., Chen, L.D., and Saarela, S. (1994). Melatonin as a free radical scavenger: implications for aging and age-related diseases. Ann. N Y Acad. Sci. *719*, 1–12.
27. Wu, Y.H., Feenstra, M.G., Zhou, J.N., Liu, R.Y., Toranõ, J.S., Van Kan, H.J., Fischer, D.F., Ravid, R., and Swaab, D.F. (2003). Molecular changes underlying reduced pineal melatonin levels in Alzheimer disease: alterations in preclinical and clinical stages. J. Clin. Endocrinol. Metab. *88*, 5898–5906.
28. Zhou, J.N., Liu, R.Y., Kamphorst, W., Hofman, M.A., and Swaab, D.F. (2003). Early neuropathological Alzheimer's changes in aged individuals are accompanied by decreased cerebrospinal fluid melatonin levels. J. Pineal Res. *35*, 125–130.
29. Matsubara, E., Bryant-Thomas, T., Pacheco Quinto, J., Henry, T.L., Poeggeler, B., Herbert, D., Cruz-Sanchez, F., Chyan, Y.J., Smith, M.A., Perry, G., et al. (2003). Melatonin increases survival and inhibits oxidative and amyloid pathology in a transgenic model of Alzheimer's disease. J. Neurochem. *85*, 1101–1108.
30. Shukla, M., Govitrapong, P., Boontem, P., Reiter, R.J., and Satayavivad, J. (2017). Mechanisms of melatonin in alleviating Alzheimer's disease. Curr. Neuropharmacol. *15*, 1010–1031.
31. Xu, W., Weissmiller, A.M., White, J.A., 2nd, Fang, F., Wang, X., Wu, Y., Pearn, M.L., Zhao, X., Sawa, M., Chen, S., et al. (2016). Amyloid precursor protein-mediated endocytic pathway disruption induces axonal dysfunction and neurodegeneration. J. Clin. Invest. *126*, 1815–1833.
32. Bressler, S.L., Gray, M.D., Sopher, B.L., Hu, Q., Hearn, M.G., Pham, D.G., Dinulos, M.B., Fukuchi, K., Sisodia, S.S., Miller, M.A., et al. (1996). cDNA cloning and

chromosome mapping of the human Fe65 gene: interaction of the conserved cytoplasmic domains of the human β-amyloid precursor protein and its homologues with the mouse Fe65 protein. Hum. Mol. Genet. *5*, 1589–1598.

33. Song, J., Durrin, L.K., Wilkinson, T.A., Krontiris, T.G., and Chen, Y. (2004). Identification of a SUMO-binding motif that recognizes SUMO-modified proteins. Proc. Natl. Acad. Sci. USA *101*, 14373–14378.

34. Niedojadlo, J., Perret-Vivancos, C., Kalland, K.H., Cmarko, D., Cremer, T., van Driel, R., and Fakan, S. (2011). Transcribed DNA is preferentially located in the perichromatin region of mammalian cell nuclei. Exp. Cell Res. *317*, 433–444.

35. Tai, D.J.C., Liu, Y.C., Hsu, W.L., Ma, Y.L., Cheng, S.J., Liu, S.Y., and Lee, E.H.Y. (2016). MeCP2 SUMOylation rescues *Mecp2*-mutant-induced behavioural deficits in a mouse model of Rett syndrome. Nat. Commun. *7*, 10552.

36. Ghosal, K., Vogt, D.L., Liang, M., Shen, Y., Lamb, B.T., and Pimplikar, S.W. (2009). Alzheimer's disease-like pathological features in transgenic mice expressing the APP intracellular domain. Proc. Natl. Acad. Sci. USA *106*, 18367–18372.

37. Kim, H.S., Kim, E.M., Lee, J.P., Park, C.H., Kim, S., Seo, J.H., Chang, K.A., Yu, E., Jeong, S.J., Chong, Y.H., and Suh, Y.H. (2003). C-terminal fragments of amyloid precursor protein exert neurotoxicity by inducing glycogen synthase kinase-3β expression. FASEB J. *17*, 1951–1953.

38. Lee, L., Sakurai, M., Matsuzaki, S., Arancio, O., and Fraser, P. (2013). SUMO and Alzheimer's disease. Neuromolecular Med. *15*, 720–736.

39. Sarge, K.D., and Park-Sarge, O.K. (2011). SUMO and its role in human diseases. Int. Rev. Cell Mol. Biol. *288*, 167–183.

40. Shukla, M., Htoo, H.H., Wintachai, P., Hernandez, J.F., Dubois, C., Postina, R., Xu, H., Checler, F., Smith, D.R., Govitrapong, P., and Vincent, B. (2015). Melatonin stimulates the nonamyloidogenic processing of βAPP through the positive transcriptional regulation of ADAM10 and ADAM17. J. Pineal Res. *58*, 151–165.

41. Wang, X., Wang, Z., Chen, Y., Huang, X., Hu, Y., Zhang, R., Ho, M.S., and Xue, L. (2014). FoxO mediates APP-induced AICD-dependent cell death. Cell Death Dis. *5*, e1233.

42. Alves da Costa, C., Sunyach, C., Pardossi-Piquard, R., Sévalle, J., Vincent, B., Boyer, N., Kawarai, T., Girardot, N., St George-Hyslop, P., and Checler, F. (2006). Presenilin-dependent γ-secretase-mediated control of p53-associated cell death in Alzheimer's disease. J. Neurosci. *26*, 6377–6385.

43. Tai, D.J.C., Hsu, W.L., Liu, Y.C., Ma, Y.L., and Lee, E.H.Y. (2011). Novel role and mechanism of protein inhibitor of activated STAT1 in spatial learning. EMBO J. *30*, 205–220.

44. Pinnix, I., Ghiso, J.A., Pappolla, M.A., and Sambamurti, K. (2013). Major carboxyl terminal fragments generated by γ-secretase processing of the Alzheimer amyloid precursor are 50 and 51 amino acids long. Am. J. Geriatr. Psychiatry *21*, 474–483.

45. Oestereich, F., Bittner, H.J., Weise, C., Grohmann, L., Janke, L.K., Hildebrand, P.W., Multhaup, G., and Munter, L.M. (2015). Impact of amyloid precursor protein hydrophilic transmembrane residues on amyloid-beta generation. Biochemistry *54*, 2777–2784.

46. Yang, Y.C., Ma, Y.L., Chen, S.K., Wang, C.W., and Lee, E.H.Y. (2003). Focal adhesion kinase is required, but not sufficient, for the induction of long-term potentiation in dentate gyrus neurons *in vivo*. J. Neurosci. *23*, 4072–4080.

47. Chao, C.C., Ma, Y.L., and Lee, E.H.Y. (2011). Brain-derived neurotrophic factor enhances Bcl-xL expression through protein kinase casein kinase 2-activated and nuclear factor kappa B-mediated pathway in rat hippocampus. Brain Pathol. *21*, 150–162.

YMTHE, Volume *29*

Supplemental Information

Melatonin Induction of APP Intracellular Domain 50 SUMOylation Alleviates AD through Enhanced Transcriptional Activation and Aβ Degradation

Yen-Chen Liu, Wei-Lun Hsu, Yun-Li Ma, and Eminy H.Y. Lee

Supplementary Figure 1

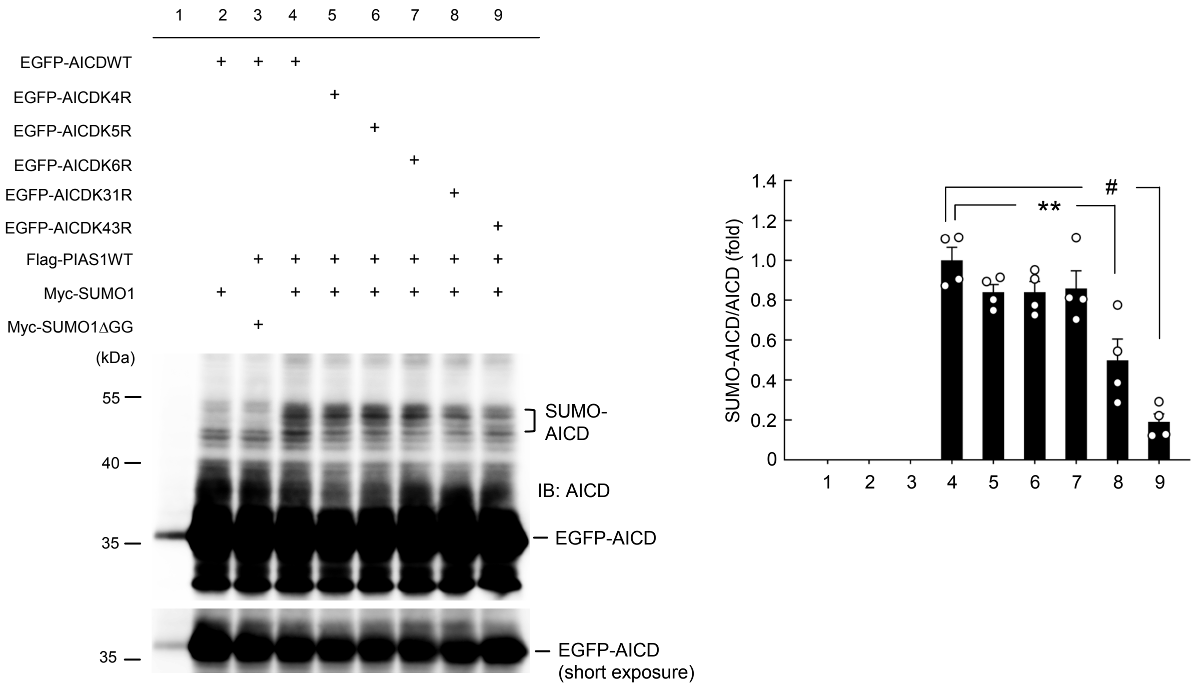

Supplementary Figure 2

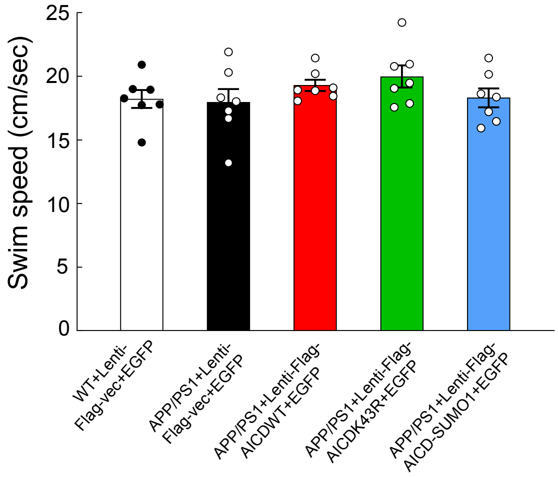

Supplementary Figure 3

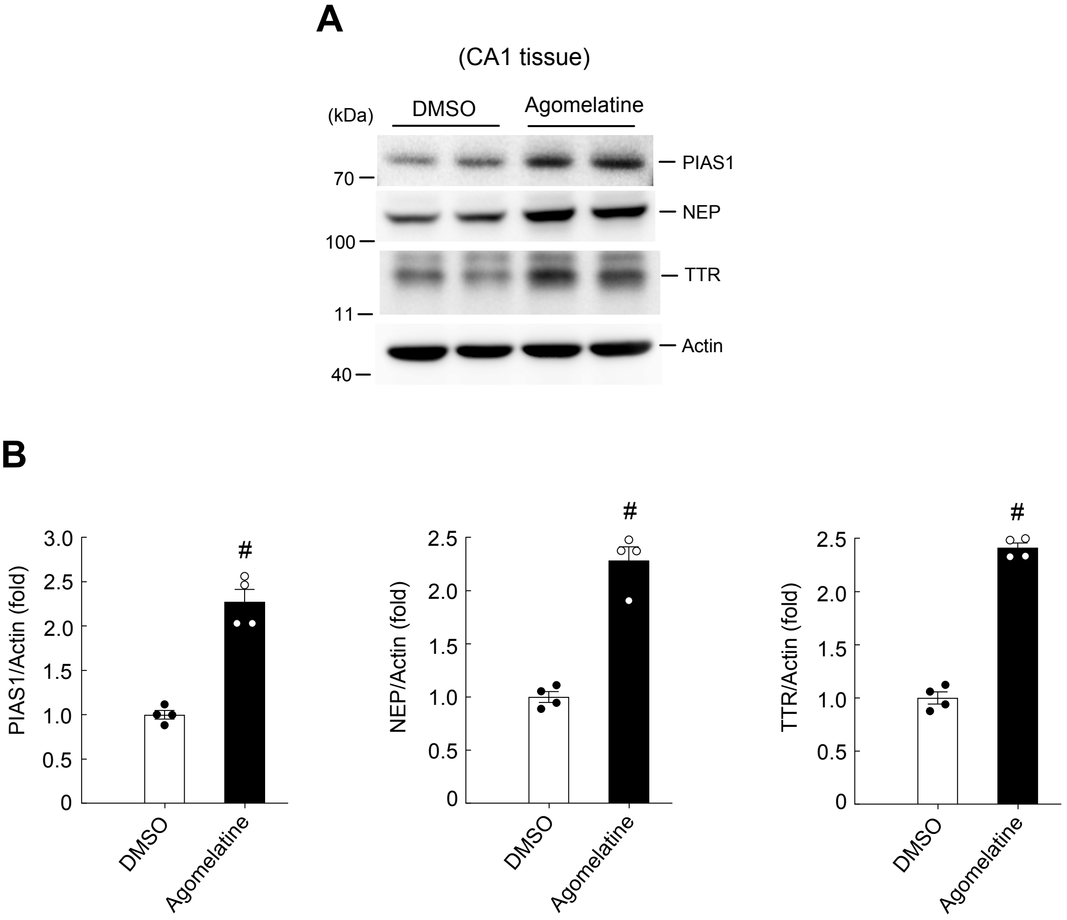

Supplementary Figure 4

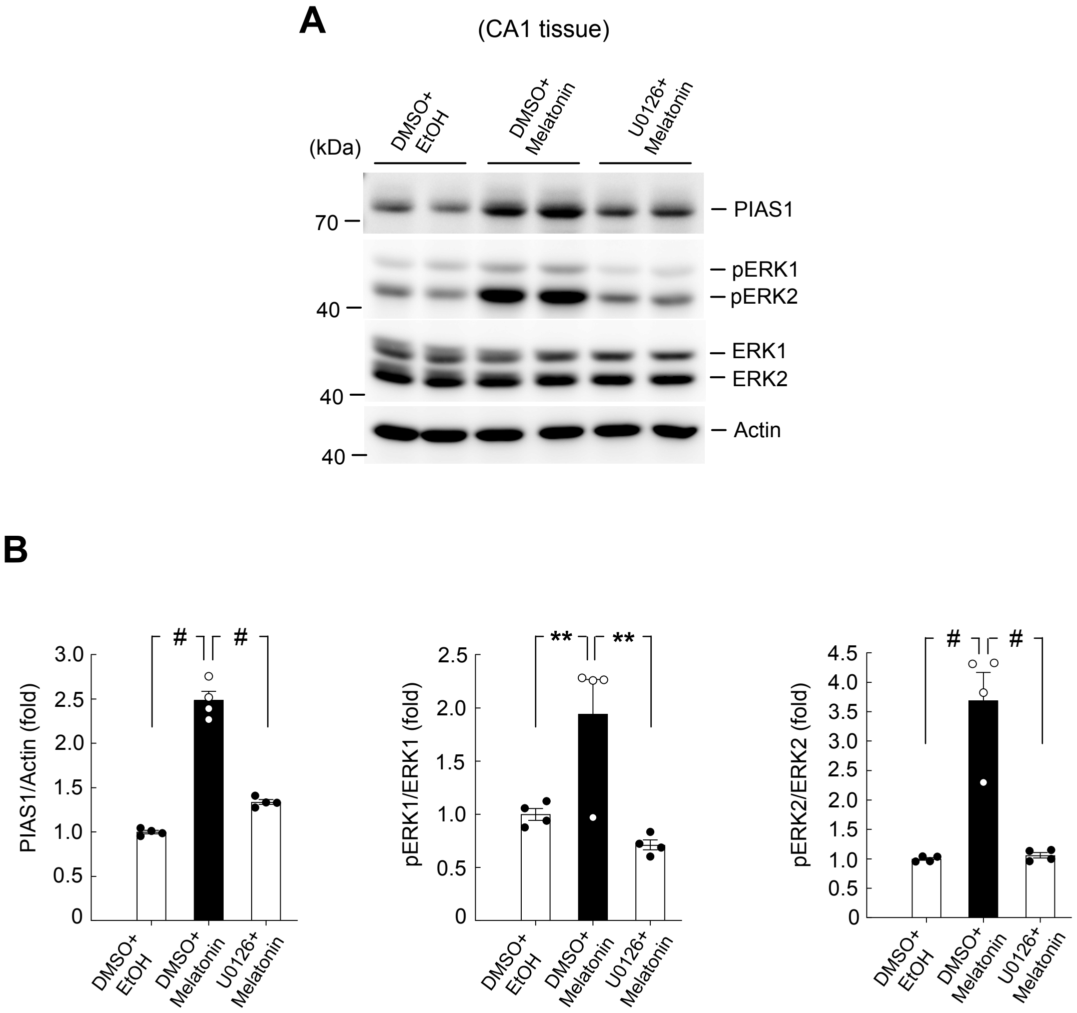

Supplementary Figure 5

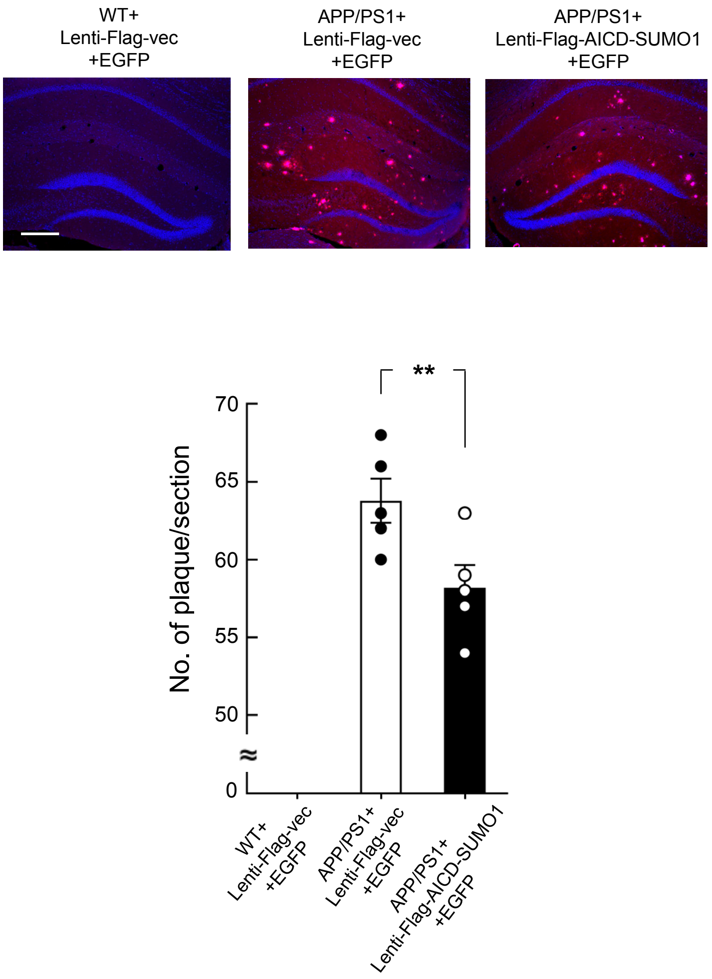

Supplementary Figure 6

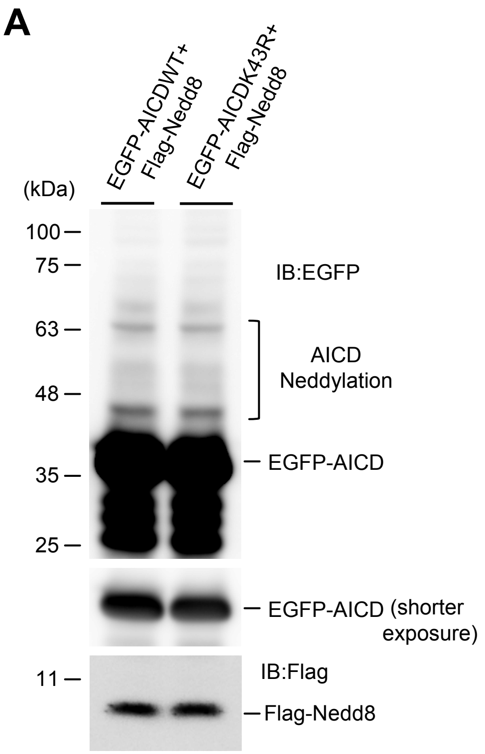

Supplementary Figure 7 All original blots

Figure 1

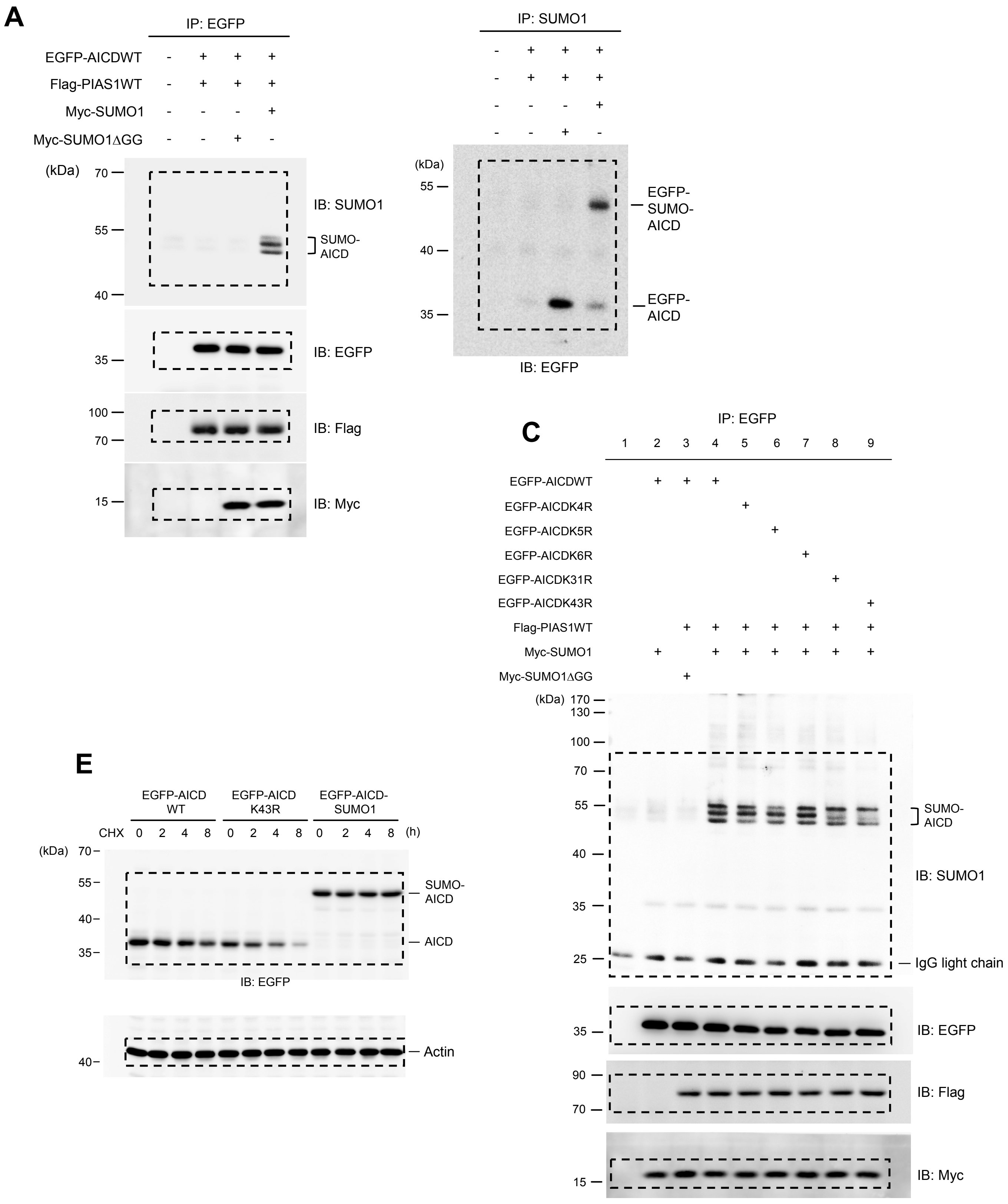

Figure 2

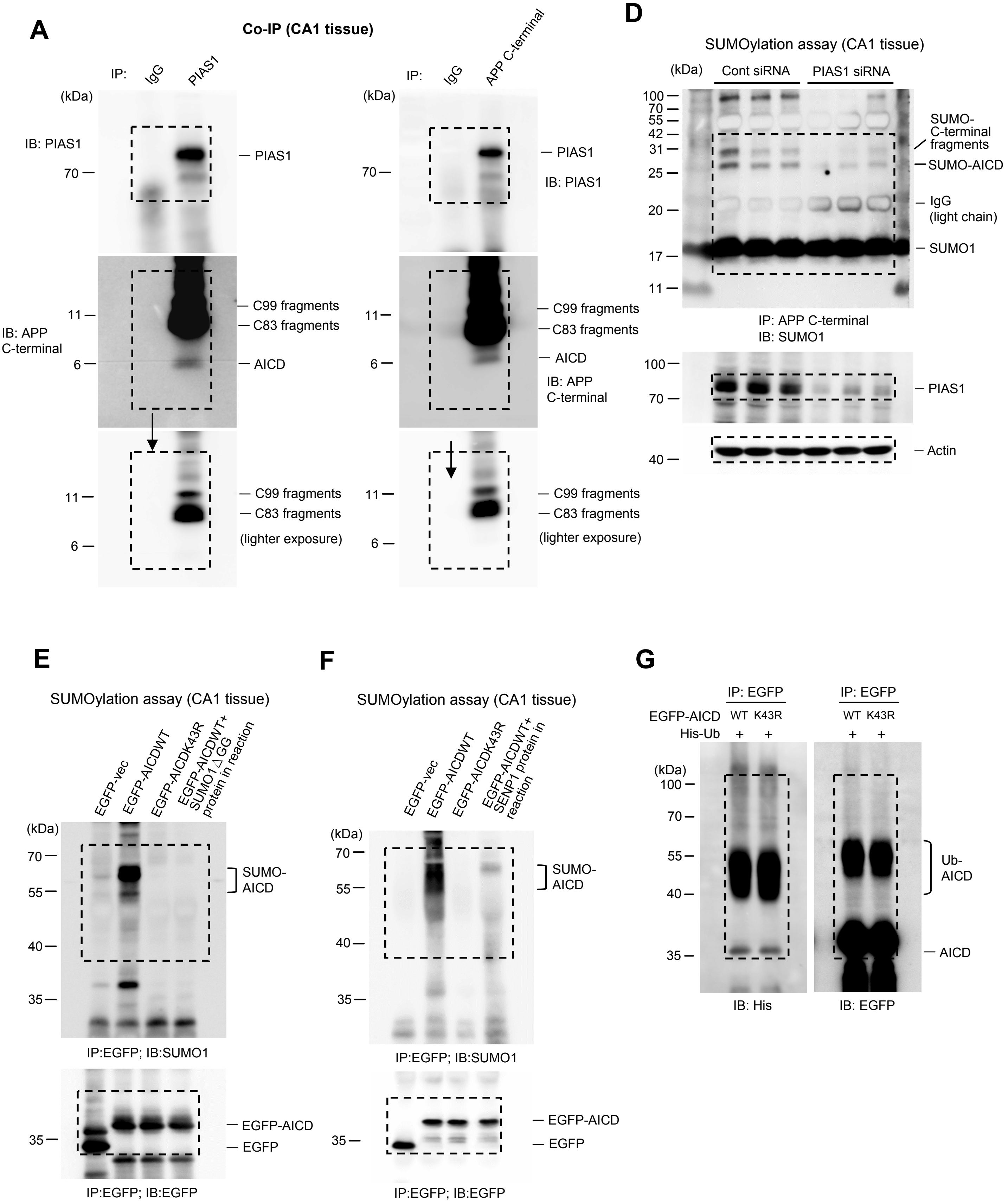
A
Co-IP (CA1 tissue)
IP: IgG PIAS1
(kDa)
IB: PIAS1
70
PIAS1
IB: APP C-terminal
11
6
C99 fragments
C83 fragments
AICD
11
6
C99 fragments
C83 fragments
(lighter exposure)
IP: IgG APP C-terminal
(kDa)
70
PIAS1
IB: PIAS1
11
6
C99 fragments
C83 fragments
AICD
IB: APP C-terminal
11
6
C99 fragments
C83 fragments
(lighter exposure)
D
SUMOylation assay (CA1 tissue)
(kDa)
Cont siRNA
PIAS1 siRNA
100
70
55
42
31
25
20
17
11
SUMO-C-terminal fragments
SUMO-AICD
IgG (light chain)
SUMO1
IP: APP C-terminal
IB: SUMO1
100
70
PIAS1
40
Actin
E
SUMOylation assay (CA1 tissue)
EGFP-vec
EGFP-AICDWT
EGFP-AICDK43R
EGFP-AICDWT+ SUMO1△GG protein in reaction
(kDa)
70
55
40
35
SUMO-AICD
IP:EGFP; IB:SUMO1
35
EGFP-AICD
EGFP
IP:EGFP; IB:EGFP
F
SUMOylation assay (CA1 tissue)
EGFP-vec
EGFP-AICDWT
EGFP-AICDK43R
EGFP-AICDWT+ SENP1 protein in reaction
(kDa)
70
55
40
35
SUMO-AICD
IP:EGFP; IB:SUMO1
35
EGFP-AICD
EGFP
IP:EGFP; IB:EGFP
G
IP: EGFP
IP: EGFP
EGFP-AICD WT K43R WT K43R
His-Ub + + + +
(kDa)
100
70
55
40
35
Ub-AICD
AICD
IB: His
IB: EGFP

Figure 3

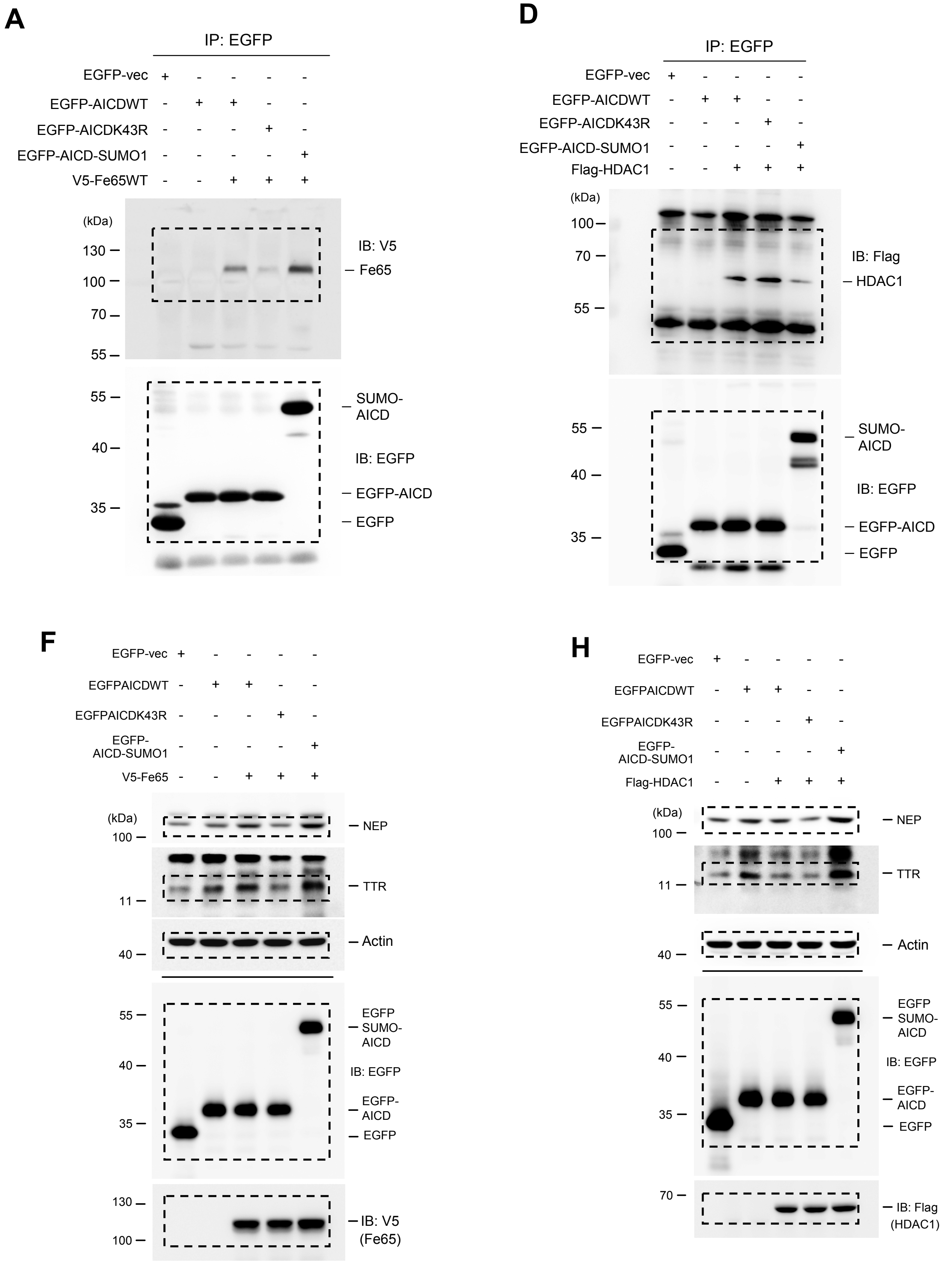
A
IP: EGFP
EGFP-vec
EGFP-AICDWT
EGFP-AICDK43R
EGFP-AICD-SUMO1
V5-Fe65WT
(kDa)
130
100
70
55
IB: V5
Fe65
55
40
35
SUMO-AICD
IB: EGFP
EGFP-AICD
EGFP
D
IP: EGFP
EGFP-vec
EGFP-AICDWT
EGFP-AICDK43R
EGFP-AICD-SUMO1
Flag-HDAC1
(kDa)
100
70
55
IB: Flag
HDAC1
55
40
35
SUMO-AICD
IB: EGFP
EGFP-AICD
EGFP
F
EGFP-vec
EGFPAICDWT
EGFPAICDK43R
EGFP-AICD-SUMO1
V5-Fe65
(kDa)
100
NEP
11
TTR
40
Actin
55
40
35
EGFP SUMO-AICD
IB: EGFP
EGFP-AICD
EGFP
130
100
IB: V5 (Fe65)
H
EGFP-vec
EGFPAICDWT
EGFPAICDK43R
EGFP-AICD-SUMO1
Flag-HDAC1
(kDa)
100
NEP
11
TTR
40
Actin
55
40
35
EGFP SUMO-AICD
IB: EGFP
EGFP-AICD
EGFP
70
IB: Flag (HDAC1)

Figure 4

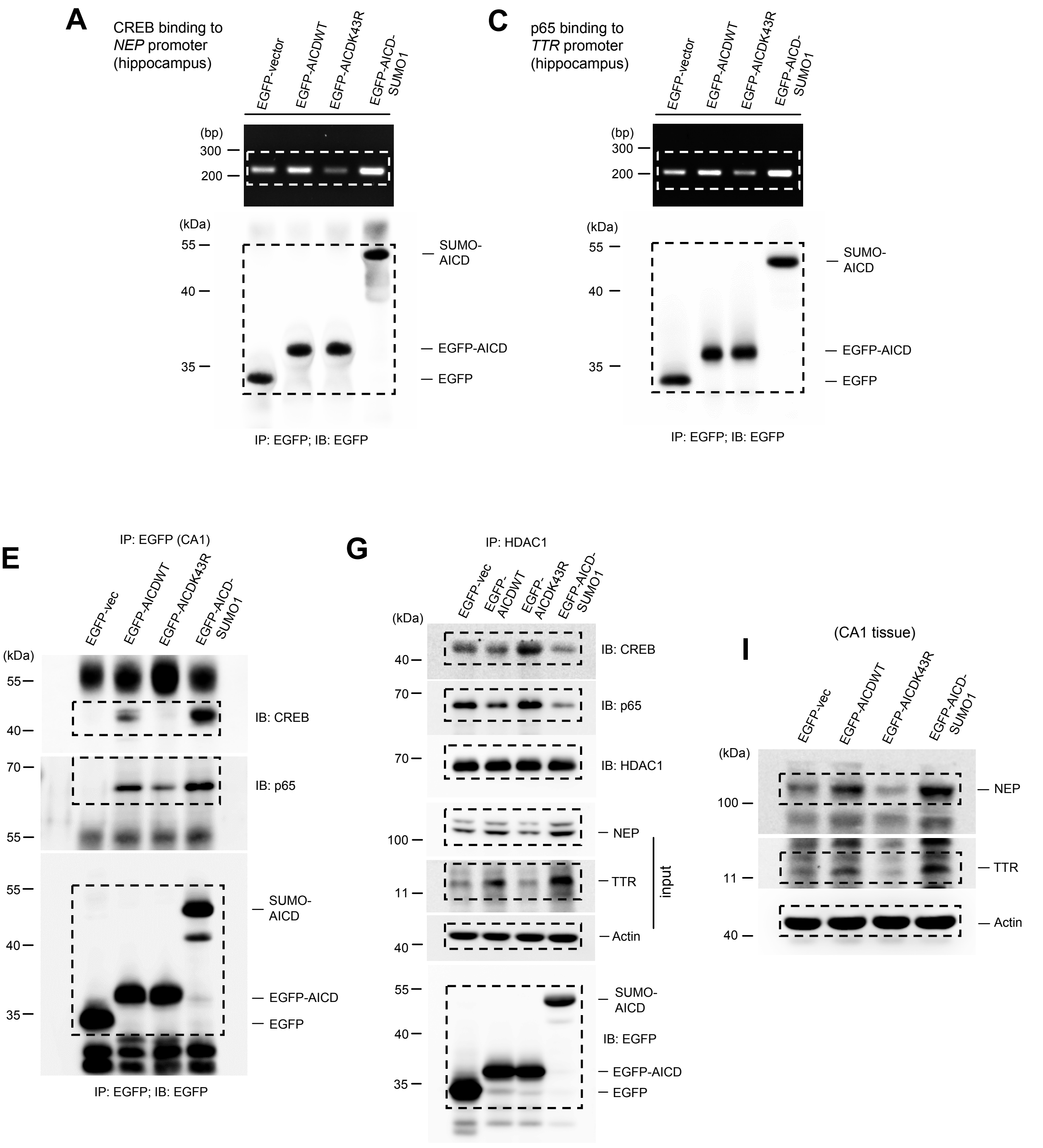
A
CREB binding to
NEP promoter
(hippocampus)
EGFP-vector
EGFP-AICDWT
EGFP-AICDK43R
EGFP-AICD-SUMO1
(bp)
300
200
(kDa)
55
40
35
SUMO-AICD
EGFP-AICD
EGFP
IP: EGFP; IB: EGFP
C
p65 binding to
TTR promoter
(hippocampus)
EGFP-vector
EGFP-AICDWT
EGFP-AICDK43R
EGFP-AICD-SUMO1
(bp)
300
200
(kDa)
55
40
35
SUMO-AICD
EGFP-AICD
EGFP
IP: EGFP; IB: EGFP
E
IP: EGFP (CA1)
EGFP-vec
EGFP-AICDWT
EGFP-AICDK43R
EGFP-AICD-SUMO1
(kDa)
55
40
70
55
55
40
35
IB: CREB
IB: p65
SUMO-AICD
EGFP-AICD
EGFP
IP: EGFP; IB: EGFP
G
IP: HDAC1
EGFP-vec
EGFP-AICDWT
EGFP-AICDK43R
EGFP-AICD-SUMO1
(kDa)
40
70
70
100
11
40
55
40
35
IB: CREB
IB: p65
IB: HDAC1
NEP
TTR
Actin
input
SUMO-AICD
IB: EGFP
EGFP-AICD
EGFP
I
(CA1 tissue)
EGFP-vec
EGFP-AICDWT
EGFP-AICDK43R
EGFP-AICD-SUMO1
(kDa)
100
11
40
NEP
TTR
Actin

Figure 5

A

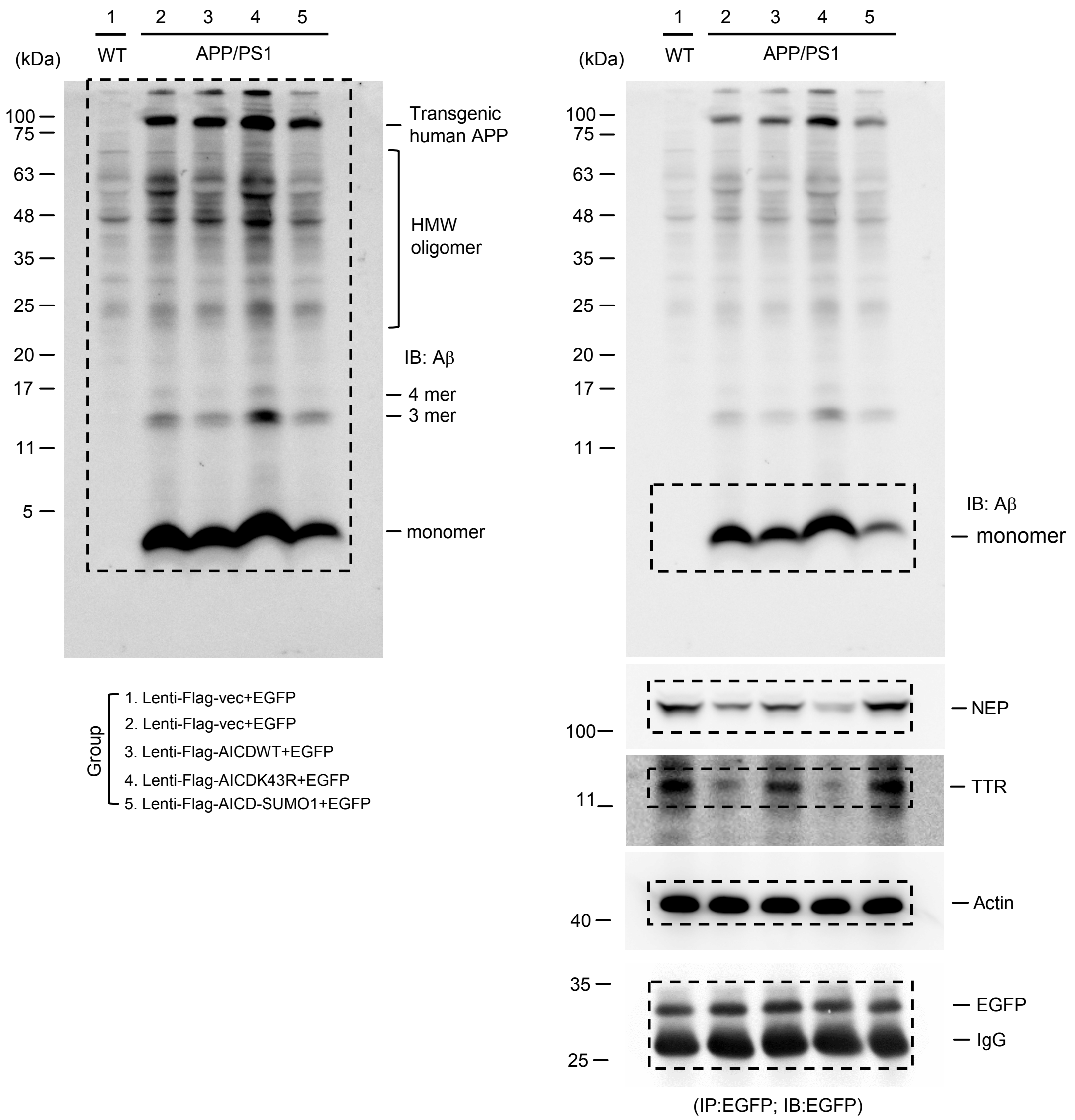
1
2
3
4
5
WT
APP/PS1
(kDa)
100
75
63
48
35
25
20
17
11
5
Transgenic human APP
HMW oligomer
IB: Aβ
4 mer
3 mer
monomer
Group
1. Lenti-Flag-vec+EGFP
2. Lenti-Flag-vec+EGFP
3. Lenti-Flag-AICDWT+EGFP
4. Lenti-Flag-AICDK43R+EGFP
5. Lenti-Flag-AICD-SUMO1+EGFP
NEP
TTR
Actin
40
EGFP
IgG
(IP:EGFP; IB:EGFP)

Figure 6

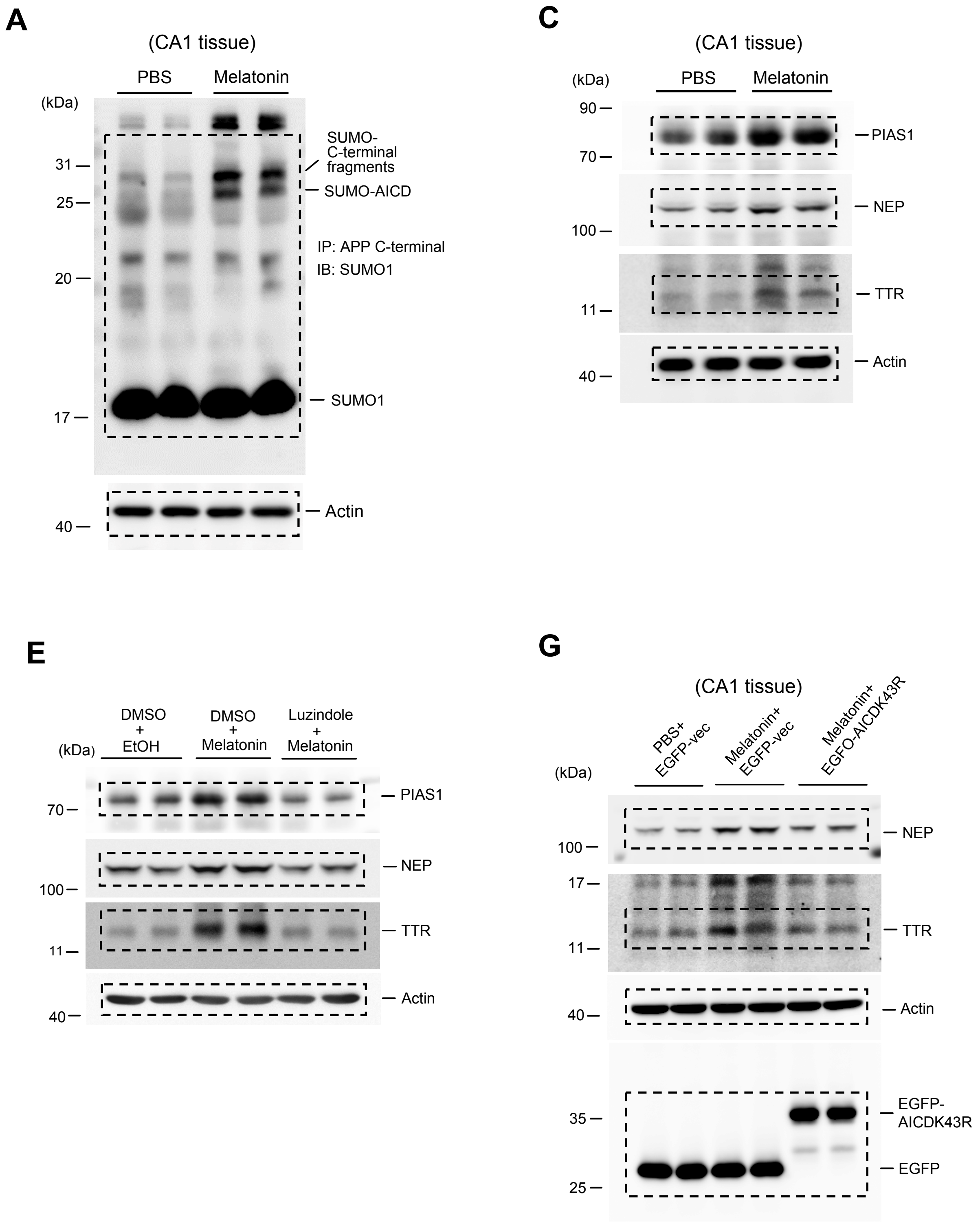
A
(CA1 tissue)
PBS
Melatonin
(kDa)
31
25
20
17
40
SUMO-
C-terminal
fragments
SUMO-AICD
IP: APP C-terminal
IB: SUMO1
SUMO1
Actin
C
(CA1 tissue)
(kDa)
PBS
Melatonin
90
70
100
11
40
PIAS1
NEP
TTR
Actin
E
DMSO
+
EtOH
DMSO
+
Melatonin
Luzindole
+
Melatonin
(kDa)
70
100
11
40
PIAS1
NEP
TTR
Actin
G
(CA1 tissue)
PBS+
EGFP-vec
Melatonin+
EGFP-vec
Melatonin+
EGFO-AICDK43R
(kDa)
100
17
11
40
35
25
NEP
TTR
Actin
EGFP-
AICDK43R
EGFP

Figure 7

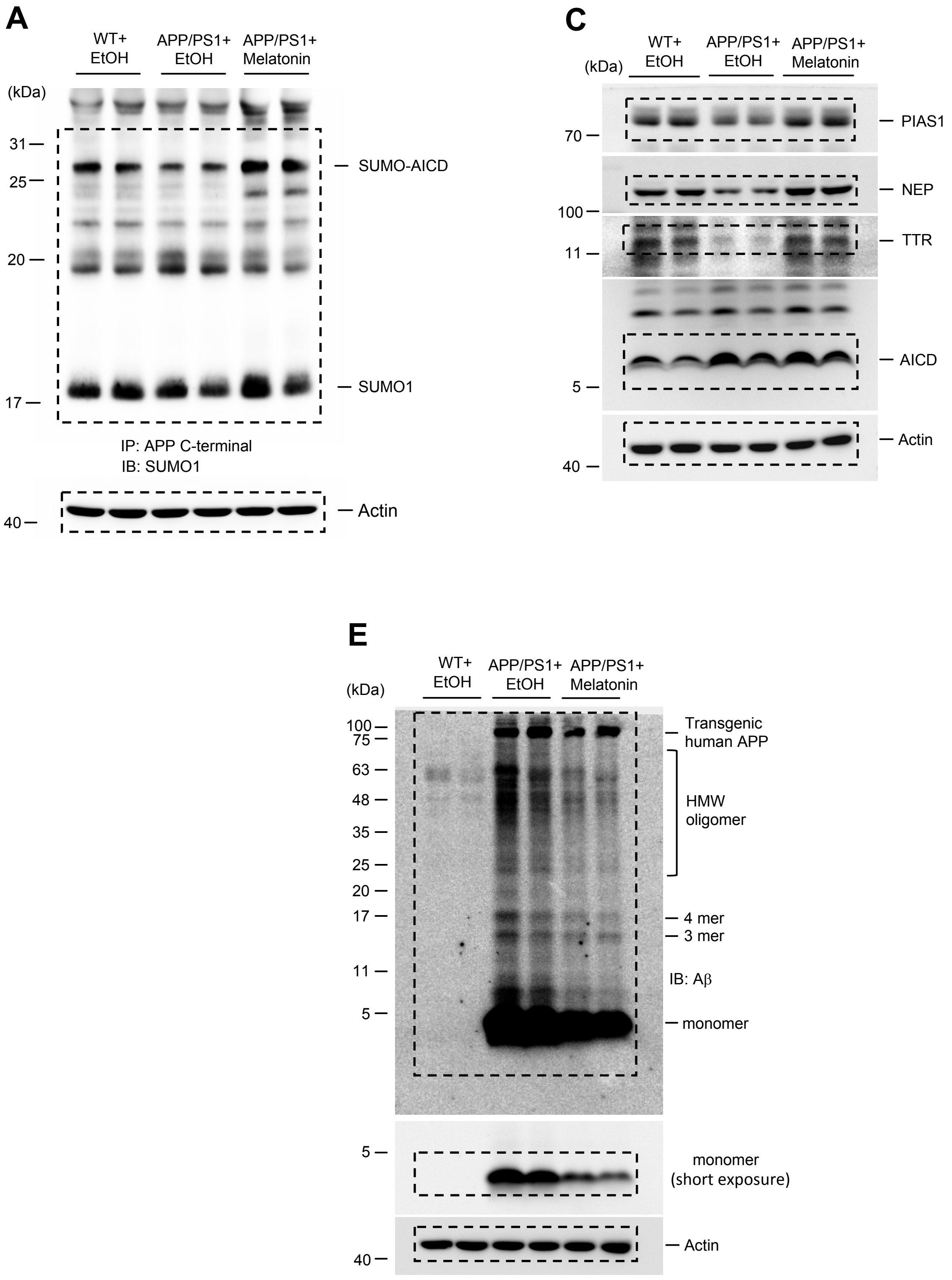
A
WT+ EtOH
APP/PS1+ EtOH
APP/PS1+ Melatonin
(kDa)
31
25
20
17
SUMO-AICD
SUMO1
IP: APP C-terminal
IB: SUMO1
40
Actin
C
WT+ EtOH
APP/PS1+ EtOH
APP/PS1+ Melatonin
(kDa)
70
PIAS1
100
NEP
11
TTR
5
AICD
40
Actin
E
WT+ EtOH
APP/PS1+ EtOH
APP/PS1+ Melatonin
(kDa)
100
75
63
48
35
25
20
17
11
5
Transgenic human APP
HMW oligomer
4 mer
3 mer
IB: Aβ
monomer
5
monomer (short exposure)
40
Actin

Supplementary Figure Legends

Figure S1 Identification of candidate SUMO sites on AICD. EGFP-tagged AICDWT plasmid or individual lysine mutant plasmids, Flag-PIAS1 plasmid, Myc-SUMO1 (or SUMO1ΔGG) plasmid were co-transfected to HEK293T cells and the cell lysate was directly immunoblotted with anti-AICD antibody. Experiments are in four repeats and results are quantified ($F_{8,27} = 55.37$, $P < 0.001$). Data are expressed as individual values and mean±SEM. ** $P < 0.01$ and $^{\#}$ $P < 0.001$.

Figure S2 Lentiviral-AICD, AICD sumo-mutant and AICD-SUMO1 fusion vector transductions do not affect swim speed of water maze learning in APP/PS1 mice. Different lenti-Flag-AICD and EGFP co-expression vector was transducted to the hippocampus of each APP/PS1 mouse (8-9 months old) and they were subjected to water maze learning two weeks later. Probe trial was conducted the next day at the end of acquisition learning. Their swim speed for the probe trial test was shown (n=7). ($F_{4,30} = 1.2$, $P > 0.05$). Data are expressed as individual values and mean±SEM.

Figure S3 Agomelatine increases PIAS1, NEP and TTR expression in rats. (A) Rats were divided to two groups (n=4) and received DMSO (37.5%) or agomelatine (3.5 μg), melatonin receptor agonist, injection to their CA1 area. Animals were

sacrificed 1 h after agomelatine injection and their CA1 tissue was subjected to western blot determination of PIAS1, NEP and TTR expression. **(B)** The quantified result of (A) ($t_{1,6}$ = 8.6 for PIAS1, $t_{1,6}$ = 9.34 for NEP and $t_{1,6}$ = 19.34 for TTR, all P < 0.001). Data are expressed as individual values and mean±SEM. $^{\#}$ P < 0.001.

Figure S4 MAPK/ERK inhibition blocks the effect of melatonin on PIAS1 expression. (A) Rats were divided to three groups (n=4) and received DMSO (40%)+EtOH (20%), DMSO+melatonin (7 μg) or U0126 (1 μg)+melatonin (7 μg) injections to their CA1 area. The two injections were separated by 30 min apart. Animals were sacrificed 1 h after melatonin (or EtOH) injection and their CA1 tissue was subjected to western blot determination of PIAS1 expression, pERK1/2 and ERK1/2 levels. **(B)** The quantified results of (A) ($F_{2,9}$ = 152.87 for PIAS1, P < 0.001; $F_{2,9}$ = 11.2 for pERK1/ERK1, P < 0.01 and $F_{2,9}$ = 30.49 for pERK2/ERK2, P < 0.001). Data are expressed as individual values and mean±SEM. ****** P < 0.01 and $^{\#}$ P < 0.001.

Figure S5 Lentiviral AICD-SUMO1 transduction decreases the amount of amyloid plaques in aged APP/PS1 mice. APP/PS1 mice of 16 months old received different lenti-Flag-AICD and EGFP co-expression vector transductions to their hippocampus. Amyloid plaque deposits (red) were examined by Proteostat dye

staining two weeks later and were quantified (n=3, 1-2 two tissue slices for each animal) ($F_{2,12}$ = 895.82, $P < 0.001$). DAPI staining is shown in blue color. Scale bar is 200 μm. Data are expressed as individual values and mean±SEM. ** $P < 0.01$.

Figure S6 Mutation of AICD at Lys-43 does not affect AICD neddylation. (A) EGFP-AICDWT or EGFP-AICDK43R plasmid was co-transfected with Flag-Nedd8 plasmid to HEK293T cells and cell lysates were immunoblotted with anti-EGFP antibody and anti-Flag antibody 48 h later. AICD neddylation from both groups is shown. The Flag-Nedd8 expression level is also shown. **(B)** Quantified result of AICD neddylation ($t_{1,4}$ = 0.62, $P > 0.05$). Results are from three independent experiments. Data are expressed as individual values and mean±SEM.

Supplementary Methods

Drugs

Melatonin was purchased from Sigma (Catalog No. M5250, St. Louis, MO). It was dissolved in 100% alcohol and further diluted with PBS to a final concentration of 10 μg/μl in 20% alcohol. The PBS solution contains 20% alcohol and was injected to animals to serve as the control group. Melatonin was prepared immediately before infusion. Agomelatine was purchased from Sigma (Catalog No. A1362) and dissolved in 100% DMSO then diluted with PBS to a final concentration of 5 μg/μl in 37.5% DMSO. Luzindole and U0126 were also purchased from Sigma (Catalog No. L2407 and Catalog No. 662005). Both drugs were dissolved in 100% DMSO and further diluted with PBS to a final concentration of 1.5 μg/μl in 40% DMSO. A volume of 0.7 μl was injected to each side of the CA1 area in rats and 0.25 μl was injected to each side of the CA1 area in mice. For intraperitoneal melatonin injection to mice, the concentration used was 10 μg/μl and 0.03 ml was injected daily.

Plasmid DNA Construction

For construction of the Flag-tagged *AICD* plasmid, full-length *AICD50* was cloned by amplifying the human *APP* cDNA (purchased from Addgene, Catalog No. 69924) with primers 5’-ATCGGGATCCGTGATGCTGAAGAAGAAAC-3’ (forward) and

5’-ATCGGATATCGTTCTGCATCTGCTCAAAG-3’ (reversed). The PCR product was sub-cloned between the *BamHI* and *EcoRV* sites of the mammalian expression vector pCMVTag2B. For construction of the Flag-tagged *PIAS1* plasmid, full-length *pias1* was cloned by amplifying the rat hippocampal *pias1* cDNA with primers 5′-ATCGGGATCCCATGGCGGACAGTGCGGAAC-3′ and 5′-ATCGGAATTCTCAGTCCAACGAGATAATG-3′. The PCR product was sub-cloned between the *BamHI* and *EcoRI* sites of the mammalian expression vector pCMV-Tag2A. For construction of the Myc-tagged *SUMO1* plasmid, full-length *sumo1* was cloned by amplifying the mouse hippocampal *sumo1* cDNA with primers 5’-GCAACCCGGGTGTCTGACCAGGAGGCAAAACCTTC-3’ (forward) and 5’-GCAAGGTACCCTAAACCGTCGAGTGACCCCCCGT-3’ (reverse). The PCR product was cloned into the mammalian expression vector pCMV-Myc. For construction of the Flag-tagged *AICD-SUMO1* fusion plasmid, the previously cloned Myc-tagged *SUMO1* plasmid was used as a template and the *sumo1* sequence was amplified with primers 5′-ATCGGTCGACATGTCTGACCAGGAGGCAA-3′ (forward) and 5′-ATCGGGGCCCCTAAACCGTCGAGTGACCC-3′ (reverse). The *SUMO1* PCR product was sub-cloned between the *SalI* and *ApaI* sites downstream of the AICD sequence from the previously cloned Flag-tagged *AICD* plasmid. A linker sequence was present on the backbone between *AICD* and *SUMO1* in order for the

AICD-SUMO1 fusion protein to be folded in the brain and the linker sequence was DIKLIDT.[23] For construction of the EGFP-tagged *AICD* plasmid and EGFP-tagged *AICD-SUMO1* fusion plasmid, the previously cloned Flag-tagged *AICD* plasmid and Flag-tagged *AICD-SUMO1*fusion plasmid were used as templates, respectively, and the *AICD* and *AICD-SUMO1* sequences were amplified with the same forward primers 5'-ATCGCGGTCCGGTGATGCTGAAGAAGAAAC-3'. The reverse primer for *AICD* was 5'-ATCGCGGACCGTTAGTTCTGCATCTGCTC-3' and that for *AICD-SUMO1* was 5'-ATCGCGGACCGCTAAACCGTCGAGTGACC-3'. The PCR product was sub-cloned into the pEGFP-C1 expression vector with *RsrII* site. Various Flag-tagged and EGFP-tagged *AICD* mutant plasmids were generated using the QuickChange Site-Directed Mutagenesis Kit (Stratagene, La Jolla, CA). For construction of the V5-tagged *Fe65* plasmid, full-length *Fe65* was cloned by amplifying the rat hippocampal *Fe65* cDNA (GeneBank: AF_333983.1) with primers 5'-ATCGGATATCATGTCTGTTCCATCATCC-3' and 5'-ATCGCCGCGGTGGGGTCTGGGATCCTAG-3'. The PCR product was sub-cloned between the *EcoRV* and *SacII* sites of the mammalian expression vector pcDNA3.1-V5-His. The *SUMO1*ΔGG mutant plasmid was generated by using site-directed mutagenesis. For construction of the His-tagged U*biquitin* plasmid, full-length *ubiquitin* was cloned by amplifying the human *ubiquitin* cDNA with

primers 5'-ATCGCCATGGATGCAGATCTTCGTGAAGAC-3' (forward) and 5'-ATCGGGATCCTTAGACACCCCCCCTCAAGC-3' (reverse). The PCR product was sub-cloned between the *NcoI* and *BamHI* sites of the expression vector CMV-3 x His-tag vector.

Small Interference RNA (siRNA) Transfection to the Hippocampus

The sequence for PIAS1 siRNA sense strand is 5'-UCCGGAUCAUUCUAGAGCUtt-3' and that for PIAS1 siRNA antisense strand is 5'-AGCUCUAGAAUGAUCCGGAtt-3'. The sequence for CREB siRNA sense strand is: 5'-GCACUUAAGGACCUUUACUtt-3' and that for CREB siRNA antisense strand is: 5'-AGUAAAGGUCCUUAAGUGCtt-3'. The Silencer Negative Control number 1 siRNA was used as the control. They were all synthesized from Ambion, Thermo Fisher Scientific (Waltham, MA).

Lentiviral Vector Construction and Preparation

For construction of Flag-AICD, Flag-AICDK43R, Flag-AICD-SUMO1 lentivitral vectors, full-length Flag-AICD, Flag-AICDK43R and Flag-AICD-SUMO fusion plasmids were sub-cloned into the lentiviral vector pLenti-Tri-cistronic (ABM, Richmond, BC, Canada) by amplifying different Flag-AICD non-viral constructs with

different primes. The forward primer is: 5’-ATCGCTCGAGGCCACCATGGATTACAAG-3’. The reverse primer for Flag-AICD and Flag-AICDK43R is 5’-ATCGCCTAGGTTAGTTCTGCATCTGCTC-3’. The reverse primer for Flag-AICD-SUMO1 is: 5’-ATCGCCTAGGCTAAACCGTCGAGTGACC-3’. These PCR products were sub-cloned between *XhoI* and *AvrII* sites of the lentiviral vector. The EGFP sequence was cloned into the pLenti-vector, pLenti-Flag-AICD, pLenti-Flag-AICDK43R and pLenti-Flag-AICD-SUMO1 vectors to obtain a cistronic co-expressing vector. The primers used for EGFP are: 5’-ATCGAGTACTGCCACCATGGTGAGCAAGGGCGAG-3’ (forward) and 5’-ATCGGGTACCCTTGTACAGCTCGTCCATGCC-3’ (reverse). The PCR product was sub-cloned between the *ScaI* and *KpnI* sites of the lentiviral vector.

For lentivirus packaging, HEK293LTV (Cell Biolabs, San Diego, CA) were transfected with 1.5 μg of psPAX2 (Addgene plasmid #12260), 0.5 μg of pMD2.G (Addgene plasmid #12259), and 2 μg of pLenti-EGFP, Flag-AICD-EGFP, Flag-AICDK43R-EGFP, Flag-AICD-SUMO1-EGFP plasmid using 10 μl of Lipofectamine 2000 (Invitrogen) in 6-well cell culture dish. Lentiviral particles were collected using the speedy lentivirus purification solution (ABM) according to the manufacturer's protocols. Cell culture medium containing lentiviral particles was

harvested for two to three times at 12 h interval until 36 h after transfection, and it was kept at 4 °C for the collecting period. The collected culture medium was further clarified by centrifugation at 2,500 x g for 10 min and filtrated through a 0.45 µm syringe filter. The speedy lentivirus purification solution (ABM) was added into filtrated supernatant (1:9, v/v) containing lentiviral particles and mixed thoroughly by inversion. The lentiviral supernatant was centrifuged at 5,000 x g at 4 °C for 10 min. Supernatant was then discarded and the viral pellet was re-suspended in ice cold PBS. After titration, the viral stock was stored at -80 °C in aliquots. The lentivirus titer was determined by lentivirus qPCR Titer Kit (ABM) according to the manufacturer's protocols (ABM). The final concentration of the lentiviral vector used for injection to the brain is 5 x 10^8 IU/ml.

Chromatin Immunoprecipitation (ChIP) Assay

ChIP assay was performed according to the protocol of Millipore ChIP assay kit (Catalog No. 17-10085). For plasmid DNA transfection, 0.7 µl plasmid DNA complex (1.5 µg/µl) was injected to the mouse CA1 area bilaterally 48 h before sacrifice. The hippocampal tissue containing the CA1 area were washed using ice-cold PBS and fixed with 1% formaldehyde by adding formaldehyde to the ice-cold PBS for 10 min. After adding glycine to quench the un-reacted formaldehyde, tissues were

homogenized and re-suspended in cell lysis buffer plus protease inhibitor cocktail II, then changed to nuclear lysis buffer plus protease inhibitor cocktail II for sonication. The chromatin was immunoprecipitated with 2 μl rabbit anti-CREB antibody (Cell Signaling; Catalog No. 9197) or 2 μl rabbit anti-p65 antibody (Cell Signaling; Catalog No. 8242). DNA purified from the immunoprecipitated samples was subjected to PCR reaction. The forward primer for the *neprilysin* promoter is: 5'-GACATGTATTGTCGCAGT-3' (nucleotide -2911 to -2893) and the reverse primer is: 5'-CCACAAACTTCAACTTGG-3' (nucleotide -2730 to -2713). The forward primer for the *transthyretin* promoter is: 5'- GAAATATTCTTAACTGGTC-3' (nucleotide -212 to -230) and reverse primer is: 5'-GTCAATAAACAAAAAACGA-3' (nucleotide -403 to -421). The PCR product for the *neprilysin* promoter is 199 bps in length and for the *transthyretin* promoter is 210 bps in length. The PCR products were separated by 2% agarose gel electrophoresis.

Immunoprecipitation (IP) and Western Blot

For IP of PIAS1, HDAC1, APP C-terminal fragment and EGFP, the clarified lysate (0.5 mg) was immunoprecipitated with 2 μl of anti-PIAS1 antibody (Epitomics; Catalog No. 2474-1; Burlingame, CA), 2 μl of anti-HDAC1 antibody (Cell Signaling; Catalog No. 5356S; Danvers, MA), 2 μl of anti-APP C-terminal fragment antibody

(Biolegend; Catalog No. 802801) or 2 μl of anti-GFP antibody (Sigma-Aldrich; Catalog No. 11814460001; Darmtadt, Germany) at 4 °C for overnight. Two microliters of rabbit or mouse IgG were used for the control group. The protein A or G magnetic beads (30 μl, 50% slurry, GE Healthcare, Barrington, IL) were added to the IP reaction product to catch the immune complex at 4 °C for 3 h. The immune complex on beads were washed three times with washing buffer containing 20 mM HEPES (pH 7.4), 150 mM NaCl, 1 mM EDTA, 1% IGEPAL CA-630, 1 mM DTT, 50 mM β-glycerophosphate, 50 mM NaF, 10 mg/ml PMSF, 4 μg/ml aprotinin, 4 μg/ml leupeptin and 4 μg/ml pepstatin and were subjected to 8%, 10% SDS-PAGE and 13% Tris-Tricine gel followed by transferring onto the Nitrocellulose (NC) membrane (GE Healthcare). Western blot was conducted using the following antibodies: anti-PIAS1 (1:5000; Epitomics), anti-APP C-terminal fragment antibody (1:3000; Biolegend), anti-SUMO1 (1:3000; Cell Signaling; Catalog No. 4930), anti-Flag M2 (1:8000; Sigma-Aldrich; Catalog No. F1804), anti-GFP (1:8000; Sigma-Aldrich), anti-V5 (1:8000; AbD Serotec; Catalog No. MCA2895; Kidlington, UK), anti-His (1:5000; Millipore; Catalog No. OB05, Bedford, MA), anti-CREB (1:2000; Cell Signaling; Catalog No. 9197), anti-p65 (1:3000; Cell Signaling; Catalog No. 8242), anti-neprilysin (1:500; Santa Cruz Biotechnology; Catalog No. SC-46656, Dallas, TX), anti-transthyretin (1:500; Santa Cruz Biotechnology; Catalog No. SC-377517),

anti-β-amyloid (1:1000; Biolegend; Catalog No. 803001), anti-AICD (1:2000; Biolegend; Catalog No. 811901), anti-phospho-MAPK (ERK1/2) (1:5000; Cell Signaling; Catalog No. 4376), anti-MAPK (ERK1/2) (1:5000; Cell Signaling; Catalog No. 4695) and anti-actin (1:200000; Milipore; Catalog No. MAB1501) antibodies. Aβ oligomerization was conducted and measured as that described previously. The secondary antibody used was HRP-conjugated goat-anti rabbit IgG antibody or HRP-conjugated goat-anti mouse IgG antibody (1:8000, Catalog No. 111-035-003 and 115-035-003, Jackson ImmunoResearch, West Grove, PA). The secondary antibody used for co-IP experiment was HRP-conjugated goat-anti rabbit IgG light chain (1:6000, Catalog No. NBP2-75935, Novus Biologicals, Centennial, CO) or HRP-conjugated goat-anti mouse IgG light chain antibody (1:6000, Catalog No. AP200P, Sigma-Aldrich). Membrane was developed by reacting with chemiluminescence HRP substrate (Millipore) and was exposed to the LAS-3000 image system (Fujifilm, Tokyo, Japan) for visualization of protein bands. The protein bands were quantified by using the NIH Image J Software.

Immunohistochemistry

For immunohistochemical staining of PIAS1 and APP C-terminal fragment in CA1 area of the rat brain, rats were anesthetized with pentobarbital (100 mg/kg, i.p.) and

perfused with ice-cold phosphate-buffered saline followed by 4% paraformaldehyde. Brains were removed and post fixed in 30% sucrose/4% paraformaldehyde solution for 20-48 h. Brains were then frozen, cut into 30-μm sections on a cryostat and mounted on gelatin-coated slides. Brain sections were rinsed with PBS for 10 min and antigen was retrieved with 0.1 M citric acid/0.1 M sodium citrate buffer at 95 °C for 45 min followed by PBS wash for 10 min for three times. The sections were pre-incubated in a blocking solution containing 3% BSA and 0.5% Triton X-100 in PBS for 1 h. For visualization of endogenous PIAS1 and APP C-terminal fragment in hippocampal CA1 neurons, brain sections were incubated with rabbit anti-PIAS1 antibody (Epitomics; 1:200; Catalog No. 2474-1) and mouse anti-APP C-terminal fragment antibody (Biolegend; Catalog No. 802801) at 4 °C overnight. Brain sections were then washed with PBS for 10 min for three times and incubated with goat anti-mouse antibody conjugated with FITC (1:500; Jackson ImmunoResearch Laboratories; Catalog No. 115-095-003) and goat anti-rabbit antibody conjugated with Cy3 (1:500; Jackson ImmunoResearch Laboratories; Catalog No. 111-165-003) for 1 h and then washed with PBS for 10 min for three times. For immunofluorescence detection of the nucleus, tissue sections were added with 20 μl of the DAPI Fluoromount-G mounting medium (SouthernBiotech, Birmingham, AL).

For immunofluorescence detection of amyloid plaque in the CA1 area, mice were anesthetized with pentobarbital (100 mg/kg, i.p.) followed by the same procedures as described above. For visualization of endogenous amyloid plaque in APP/PS1 mice, brain sections containing the CA1 area were incubated with ProteoStat Amyloid Plaque Detection Kit (Enzo Life Sciences; Catalog No. ENZ-51035) for 30 min and washed with PBS for 10 min for three times at room temperature. This detection kit was used because we have previously demonstrated that ProteoStat dye staining well co-localizes with anti-amyloid-beta staining in the hippocampus.[22] The brain sections were then mounted with 20 μl DAPI Fluoromount-G mounting medium (SouthernBiotech). Photomicrographs were taken using a Zeiss LSM700 confocal microscope (Carl Zeiss, Oberkochen, Germany). The number of plaques showing ProteoStat dye staining was counted by using the NIH Image J Software.

Immunofluorescence

Cultured Neuro2A cells transfected with EGFP-vector, EGFP-AICD WT, EGFP-AICD K43R, EGFP-AICD SUMO1 and V5-Fe65 plasmids on glass coverslips were washed with PBS for 5 min and fixed with 4% paraformaldehyde for 10 min at room temperature followed by PBS wash for 10 min for three times. The cells were

pre-incubated in a blocking solution containing 3% BSA and 0.1% Triton X-100 in PBS for 1 h at room temperature. Primary mouse anti-V5 antibody (1:200; AbD Serotec; Catalog No. MCA2895) was added with 3% BSA and 0.1% Triton X-100 in PBS at 4°C overnight. Cultured Neuro2A cells were then washed with PBS for 10 min for three times and incubated with goat anti-mouse antibody conjugated with Cy3 (1:500; Jackson ImmunoResearch Laboratories; Catalog No. 115-165-003) for 1 h and then washed with PBS for 10 min for three times. For immunofluorescence detection of the nucleus, Neuro2A cells on glass coverslips were added with 10 μl of the DAPI Fluoromount-G mounting medium (SouthernBiotech ; Catalog No. 0100-20, Birmingham, AL). Cultured HEK293T cells transfected with EGFP-AICDWT plasmid on glass coverslips were washed with PBS for 5 min and fixed with 4% paraformaldehyde for 10 min at room temperature followed by PBS wash for 10 min for three times. The cells were pre-incubated in a blocking solution containing 3% BSA and 0.1% Triton X-100 in PBS for 1 h at room temperature. Primary rabbit anti-PIAS1 antibody (Epitomics; 1:200; Catalog No. 2474-1) was added with 3% BSA and 0.1% Triton X-100 in PBS at 4°C overnight. HKE293T cells on glass coverslips were then washed with PBS for 10 min for three times and incubated with goat anti-rabbit antibody conjugated with Cy3 (1:500; Jackson ImmunoResearch Laboratories; Cat. No. 111-165-003) for 1 h and then washed with PBS for 10 min for

three times. The HEK293T cells on glass coverslips were then mounted with 10 μl DAPI Fluoromount-G mounting medium (SouthernBiotech). Photomicrographs were taken using a Zeiss LSM700 confocal microscope (Carl Zeiss).

Water Maze Learning

The water maze used was a plastic, circular pool, 1.2 m in diameter and 25 cm in height that was filled with water (25 ± 2 °C) to a depth of 16 cm. A circular platform of 8 cm in diameter was placed at a specific location away from the edge of the pool. The top of the platform was submerged 0.6 cm below the water surface. Water was made cloudy by adding milk powder. Distinctive, visual cues were set on the wall. For spatial learning, animals were subjected to three trials a day with one given early in the morning, one given in the early afternoon and the other one given in the late afternoon. The training procedure lased for 5 days and a total of 15 trials were given. For these trials, animals were placed at different starting positions spaced equally around the perimeter of the pool in a random order. Animals were given 60 sec to find the platform. If an animal could not find the platform within 60 sec, it was guided to the platform and was allowed to stay on the platform for 20 sec. The time that each animal took to reach the platform was recorded as the escape latency. A probe trial of 60 sec was given on day 6 to test their memory retention. Animals were placed in the

pool with the platform removed and the time they spent in each quadrant (target quadrant, left quadrant, opposite quadrant and right quadrant) as well as the total distance travelled in the target quadrant were recorded.
